# Phosphoproteomic analysis reveals major default phosphorylation sites outside long intrinsically disordered regions of *Arabidopsis* plasma membrane proteins

**DOI:** 10.1186/1477-5956-10-62

**Published:** 2012-10-30

**Authors:** Claude Nespoulous, Valérie Rofidal, Nicolas Sommerer, Sonia Hem, Michel Rossignol

**Affiliations:** 1 UR1199 Laboratoire de Protéomique Fonctionnelle, INRA, 34060, Montpellier cedex, France; 2 UMR SPO, INRA, 34060, Montpellier cedex, France

**Keywords:** *Arabidopsis*, Plasma membrane, Phosphoproteome, Intrinsically disordered regions

## Abstract

**Background:**

Genome-wide statistics established that long intrinsically disordered regions (over 30 residues) are predicted in a large part of proteins in all eukaryotes, with a higher ratio in trans-membrane proteins. At functional level, such unstructured and flexible regions were suggested for years to favour phosphorylation events. In plants, despite increasing evidence of the regulation of transport and signalling processes by phosphorylation events, only few data are available without specific information regarding plasma membrane proteins, especially at proteome scale.

**Results:**

Using a dedicated phosphoproteomic workflow, 75 novel and unambiguous phosphorylation sites were identified in *Arabidopsis* plasma membrane. Bioinformatics analysis showed that this new dataset concerned mostly integral proteins involved in key functions of the plasma membrane (such as transport and signal transduction, including protein phosphorylation). It thus expanded by 15% the directory of phosphosites previously characterized in signalling and transport proteins. Unexpectedly, 66% of phosphorylation sites were predicted to be located outside long intrinsically disordered regions. This result was further corroborated by analysis of publicly available data for the plasma membrane.

**Conclusions:**

The new phosphoproteomics data presented here, with published datasets and functional annotation, suggest a previously unexpected topology of phosphorylation in the plant plasma membrane proteins. The significance of these new insights into the so far overlooked properties of the plant plasma membrane phosphoproteome and the long disordered regions is discussed.

## Background

A large part of proteins in all eukaryotes, including plants, is predicted to contain intrinsically disordered regions (IDR), concerning long stretches of more than 30 residues, in a proportion depending on their subcellular localization [1]. Notably, by comparison to soluble proteins, trans-membrane proteins are estimated to be richer in disordered regions [2] located at their cytoplasmic side, especially in the case of plasma membrane (PM) integral proteins [3]. In addition, direct assessment of IDR in published crystal structures for integral membrane proteins from various genomes and various subcellular origins showed that more than half of them actually display IDR [4]. At functional level, protein phosphorylation was suggested to occur predominantly in IDR [5]. In addition, in humans, recent proteome-wide data mining of curated information on post-translational modifications (PTM) confirmed that the frequency of phosphorylation is higher in predicted IDR and showed that this situation is mostly pronounced in the PM, where the enrichment of phosphosites within IDR reaches a factor of 2.7 [1]. Thus, as a general role of IDR in the adoption of structures favouring regulatory interactions is increasingly accepted [6], it could be speculated that integral membrane proteins use disordered regions for signalling and regulation, through various events such as reversible protein phosphorylation.

In plants, puzzling information is presently available on phosphorylation and IDR [7]. Overall, one third of protein sequences in *Arabidopsis* and rice genomes are predicted to contribute to long IDR [1,8]. Actually, some soluble proteins were predicted with a high probability to have regulatory phosphosites within IDR. This concerns notably the dehydrin family of proteins [9] and the GRAS proteins whose disordered N-terminal domain constitutes the first functionally required unfoldome in the plant kingdom [10,11]. By contrast, despite increasing functional evidence of the regulation of transport and signalling processes by phosphorylation, no specific information is available regarding membrane proteins, including the PM,. In addition, several phosphorylation datasets have been generated from the *Arabidopsis* PM [12-18] and compiled into the PhosPhAt database (http://phosphat.mpimp-golm.mpg.de, [19]). But none of these analyses addressed the relationship between phosphorylation and IDR. Interestingly, studies in mice have shown that 86% of mouse brain phosphosites are located in predicted long IDR [20]. But presently in plants, only incomplete information is available and no effort was made to combine data about phosphoproteomics and disordered regions.

In this work, using a dedicated workflow, we identified novel phosphorylation sites in *Arabidopsis* PM vesicles. This set of phosphosites is shown to concern mainly integral PM proteins, mostly involved in transport and signal transduction Thus It allows to highlight original features regarding the location of phosphorylation sites in structured *vs* unstructured regions.

## Results and discussion

Tryptic peptides from the enriched PM fraction were analyzed using a workflow designed to identify phosphorylated peptides and screen for novel phosphorylation sites (Additional file 1: Figure S1). For this purpose, we used a combination of peptide fractionation by Strong Anion eXchange (SAX) chromatography and phosphopeptide enrichment by TiO_2_. This combination was previously shown to be able to identify specific subset of phosphorylation sites from PM transporters [16]. Tryptic peptides were then analyzed by LC-ESI MS/MS. Secondly, the resulting MS/MS data were queried against the *Arabidopsis* TAIR9 (http://www.arabidopsis.org/) database in the target-decoy mode in order to select peptides phosphorylated with 1% false discovery rate. The location of phosphorylated residues in these *bona fide* phosphopeptides was then assessed from their PTM score [21,22] and only unambiguous phosphorylation sites were selected. Finally, our dataset was compared with the PhosPhAt database and those sites that were not described previously were selected for further analysis. Overall, 411 phophorylated peptides were characterized, resulting in the identification of 298 phosphoproteins and 559 unique phosphorylation sites. From this data set, the workflow allowed the characterization of 75 novel and unambiguous phosphorylation sites in 66 phosphopeptides corresponding to 52 protein accessions (Table 1, Additional file 2).

**Table 1:** Main features of proteins showing novel phosphosites

	**Accessions**	**Phosphosites**	**TMD**	**PM prediction**
Total	52	75	40	47
Transport	19	29	19	17
Signalling	18	22	14	18
Miscellaneous	12	21	4	9
Not assigned	3	3	3	3

Features were computed from the resources described in main text. TMD: number of proteins with at least one trans-membrane domains (Aramemnon database); PM: number of proteins with a PM location (SUBA database). Proteins were classified in functional categories according to TAIR annotations and MapMan ontology.

The distribution of modifications in terms of nature of the phosphorylated residues pSer/pThr/pTyr was found to be 75%/21%/4%, respectively. In addition, at the phosphorylation level, beside a majority of mono-phosphorylated species, multi-phosphorylated peptides accounted for one quarter of the total, of which 25% carried more than 2 modifications. The small size of the present phosphoproteome prevents definitive conclusions to be derived. However, the number of multi-phosphorylated peptides differed to some extent from previous *Arabidopsis* studies (often below 10% [14,15]) while the proportion of phosphotyrosines ranges between 0% and 4% as reported in PM [13-15] as well as in whole cell [23] studies. As similar cell culture conditions were used in previous *Arabidospsis* PM studies, the new features observed in our work do not arise from differences linked to the biological material. They should be thus attributed to our specific workflow and should be investigated further.

### The phosphoproteome displays features expected for PM proteins

Plasma membrane vesicles were prepared from *Arabidopsis* cell suspension by differential centrifugation and phase partitioning between polyethylene glycol and dextran. Measurement of phosphohydrolase activities (Additional file 3: Figure S2) showed that the total Mg-dependent ATPase activity was over 95% sensitive to vanadate, a specific inhibitor of the plasma membrane H^+^-ATPase, and to a lesser extent to nitrate, with low azide-sensitive component. In addition, IDPase activity was below 5% of the ATPase activity. Collectively, this showed the prevalence of phosphohydrolase activity of the Mg-dependent and vanadate-sensitive H^+^-ATPase type. This indicated that the membrane fraction was enriched in PM, with limited contamination by endomembranes, in agreement with results obtained using similar procedures for cell cultures or other samples from *Arabidopsis*[14,24].

According to the SUB-cellular location database of A*rabidopsis* proteins (SUBA, http://suba.plantenergy.uwa.edu.au/) and to TAIR annotations, 90% of the newly identified phosphoproteins were known or predicted to have a PM location (Table 1, “PM prediction” column). In addition, nearly 80% of accessions were predicted to display at least one trans-;membrane domain (TMD) by the Aramemnon plant membrane protein data-base (http://aramemnon.botanik.uni-koeln.de/) (Table 1, “TMD” column).

Thus, both the above biochemical characterization of the membrane fraction and features of identified proteins indicate that the present subset of proteins corresponds mostly to genuine PM proteins, including a high proportion of integral proteins.

In order to get further information about the function of these proteins, we used both the *Arabidopsis* MapMan ontology (http://mapman.gabipd.org/web/guest/home) and TAIR annotations. The phosphoproteome identifies typical major PM functions. Indeed, two main categories emerged accounting for 72% of the dataset. The first category included protein transporters and the second proteins involved in signalling processes or protein phosphorylation. Each one of them consisted of more than one third of the total proteins (Table 1). The subset of transporters included both ion and small molecules transporters (*e.g.* phosphate transporters and auxin carriers) (Table 2). All of them were estimated to have a PM location and possessed between 6 and 15 TMD, at the exception of a magnesium transporter. This latter was predicted to display only 2 TMD and for which no previous information about a PM location was available. A large part of signalling proteins corresponded to kinases from the Receptor-Like Kinases (RLK) super-family. This family included notably members from the Leucine-Rich Repeat RLK (LRR-RLK) sub-family, most of them lacking yet a known role in a characterized process, with the exception of two alleles of brassinosteroid receptor BRI1. Proteins involved in protein phosphorylation covered various types of kinases, like Calcium-dependent Protein Kinases, together with one protein tyrosine phosphatase. Information about PM location was available for all proteins in these categories, and the presence of at least one TMD was predicted for 80% of them (Table 1). The privileged identification of these functional classes is in agreement with published proteomics and phosphoproteomics studies about the *Arabidopsis* PM [12,15,24].

**Table 2:** Identified phosphorylated proteins, peptides and novel sites

**Protein**				**Peptide**				**Site**	
**Accession**	**Protein name**	**PM SUBA**	**TMD**	**Mascot score**	**PTM score**	**Location**	**Sequence**	**Location**	**IDR**
*Transporter*									
AT1G23080.1	Auxin efflux carrier family protein	PM	10	71	114	[177–201]	VE[S]DVV[S]LDGHDFLETDAQIGDDGK	S179	out
								S183	out
AT1G47670.1	transmembrane amino acid transporter family protein	PM	11	58	163	[18–30]	V[S][T]PEIL[T]PSGQR	S19	in
								T20	in
								T25	in
AT1G48370.1	YELLOW STRIPE like 8	PM	15	58	80	[50–63]	EEQEE[S]VEGIFESR	S55	in
AT1G76430.1	phosphate transporter 1;9		12	49	67	[506–529]	SLEENEDEIVSt(s)AG(s)[S]PANELLR	S522	out
AT2G01420.1	Auxin efflux carrier family protein	PM	10	71	73	[177–201]	VE[S]DVV[S]LDGHDFLETDAEIGNDGK	S179	out
								S183	out
AT2G01980.1	sodium proton exchanger, putative (NHX7) (SOS1)	PM	11	66	90	[1114–1133]	QNTMVE[S][S]DEEDEDEGIVVR	S1120	out
								S1121	out
AT2G28070.1	ABC-2 type transporter family protein	PM	6	54	66	[41–60]	QPISFED[S]PEWED[T]PDVDLR	S48	out
								T54	out
AT2G28120.1	Major facilitator superfamily protein	PM	11	46	102	[556–569]	E[S]PESESELVPDSR	S557	out
AT2G32830.1	phosphate transporter 1;5	PM	12	48	107	[518–538]	EDEEQSGGD[T]VVEMTVANSGR	T527	in
AT3G55320.1	putative subfamily B ABC-type transporter (AtMDR14)	PM	12	46	92	[767–785]	(s)NG(s)EPE[S]PVSPLLTSDPK	S774	in
AT4G23700.1	cation/H+ exchanger 17	PM	12	106	379	[806–820]	NVTTEESLVEDSE[S]P	S819	out
				96	244	[806–820]	NVTTEESLVED[S]E[S]P	S817	out
AT4G24120.1	YELLOW STRIPE like 1	PM	14	98	148	[12–38]	EGEEEEDNNQLSLQEEEPD[T]EEEMSGR	T31	in
AT4G29900.1	autoinhibited Ca(2+)-ATPase 10	PM	9	84	132	[15–37]	DVEAGTS[S]FTEYEDSPFDIASTK	S22	out
AT5G01240.1	like AUXIN RESISTANT 1	PM	10	66	88	[6–22]	QAEESIVV[S]GEDEVAGR	S14	in
				125	202	[24–44]	VED[S]AAEEDIDGNGGNGFSMK	S27	in
AT5G24030.1	SLAC1 homologue 3	PM	9	83	125	[599–610]	NV[S]SENIENYLK	S601	in
AT5G43350.1	phosphate transporter 1;1	PM	12	72	158	[260–270]	VLQ[T]DIELEER	T263	out
				111	70	[509–524]	SLEELSGEAEV[S]HDEK	S520	out
				50	47	[509–524]	[S]LEELSGEAEV[S]HDEK	S509	out
AT5G45380.1	sodium symporters;urea transmembrane transporters	PM	15	86	191	[552–571]	VVEA[Y]ASGDEDVDVPAEELR	Y556	out
AT5G64410.1	oligopeptide transporter 4	PM	15	81	136	[2–19]	ATADEF[S]DEDTSPIEEVR	S8	out
AT5G64560.1	magnesium transporter 9		2	54	53	[130–151]	EIAGAQNDGD[T]GDEDESPFEFR	T140	out
*Signalling*									
AT1G05150.1	Calcium-binding tetratricopeptide family protein	PM		65	65	[171–188]	ADNNNNNVDAFSDAGW[S]R	S187	in
AT1G11330.1	S-locus lectin protein kinase family protein	PM	2	66	57	[556–572]	[S]GQGLEELMNEVVVISK	S556	out
AT1G53440.1	Leucine-rich repeat transmembrane protein kinase	PM	1	56	199	[1025–1035]	LLDDL[T]DVEIE	T1030	in
AT1G55610.1	BRI1 like	PM	1	90	153	[1139–1153]	AD[T]EEDESLDEFSLK	T1141	out
				46	118	[1139–1153]	AD[T]EEDE[S]LDEFSLK	S1146	out
AT1G71860.1	protein tyrosine phosphatase 1	PM		47	106	[19–30]	FDLSSAD[S]PPSK	S26	out
AT3G13380.1	BRI1-like 3	PM	1	87	199	[1134–1151]	ELVQVDTEND[S]LDEFLLK	S1144	out
AT3G13530.1	mitogen-activated protein kinase kinase kinase 7	PM		55	109	[481–510]	VSEGKPNEASTSMPTSNVNQGD[S]PVADGGK	S503	in
AT3G24660.1	Transmembrane kinase-like	PM	2	54	109	[329–350]	K(s)(s)IE[S]EDDLEEGDEEDEIGEK	S334	in
AT3G25070.1	RPM1 interacting protein 4	PM		59	30	[37–61]	IMNPNDPE[Y]NSDSQSQAPPHPPSSR	Y45	in
AT3G28450.1	Leucine-rich repeat protein kinase family protein	PM	2	69	128	[263–276]	[S]GLTEVGVSGLAQR	S263	out
AT3G51740.1	inflorescence meristem receptor-like kinase 2	PM	1	65	155	[755–768]	EEW[T]NEVFDLELMR	T758	out
AT4G23190.1	cysteine-rich RLK (RECEPTOR-like protein kinase) 11	PM	1	61	140	[324–341]	[T]ESE[S]DI[S]TTDSLVYDFK	T324	out
								S328	out
								S331	out
AT4G24630.1	DHHC-type zinc finger family protein	PM	4	58	133	[329–344]	VEDDLDIGDDLMNL[S]R	S343	out
AT4G36180.1	Leucine-rich receptor-like protein kinase family protein	PM	1	74	236	[1119–1136]	VGPDVPSSADPTSQP[S]PA	S1134	out
AT5G05160.1	Leucine-rich repeat protein kinase family protein	PM	1	59	167	[564–577]	EEW[T]AEVFDVELLK	T567	out
AT5G19450.1	calcium-dependent protein kinase 19	PM	1	52	56	[23–41]	SNPFYSEA[Y]TTNGSGTGFK	Y31	in
AT5G58300.1	Leucine-rich repeat protein kinase family protein	PM	1	46	48	[359–366]	A[S]AEVLGK	S360	out
				71	119	[577–590]	EEW[T]SEVFDIELMR	T580	out
AT5G56890.1	Protein kinase superfamily protein	PM	1	77	122	[1041–1055]	YPLLPNYD[S]EPDTER	S1049	in
*Miscellaneous*									
AT1G10340.2	Ankyrin repeat family protein	PM	4	72	31	[331–358]	FGTETSQELD[S]ENNVEQHEGSQEVEVIR	S341	out
AT1G68720.1	tRNA arginine adenosine deaminase	PM		72	119	[1091–1101]	D[S]FEEWEEAYK	S1092	out
AT2G41705.1	camphor resistance CrcB family protein	PM	9	60	111	[31–54]	SLPHLIDNDVD[S]ESVSEAGDIGDR	S42	out
				65	98	[31–54]	SLPHLIDNDVD(s)E[S]V(s)EAGDIGDR	S44	out
				57	101	[31–54]	SLPHLIDNDVD[S]E[S]V[S]EAGDIGDR	S46	out
				60	145	[31–54]	[S]LPHLIDNDVD[S]E[S]V[S]EAGDIGDR	S31	out
				81	112	[68–83]	L[S]ADDFIEQGTHDTSR	S69	in
				63	118	[103–116]	TLPEDITA[S]PLPTK	S111	out
				55	100	[117–128]	SLL[S]PEINNSGK	S120	out
AT3G09770.1	RING/U-box superfamily protein	PM		68	67	[290–317]	YELQEIYGIGN[T]VEGDDDSADDANDPGK	T301	out
AT3G27530.1	golgin candidate 6			74	271	[897–914]	LLEDIGDESEAQAE[S]EED	S911	out
AT3G28850.1	Glutaredoxin family protein	PM		48	111	[18–27]	GY[S]PPVDVQR	S20	out
				56	88	[167–178]	[S]FSFDVGPNGGK	S167	out
				51	18	[381–405]	VYYEYEDDDDDDDEGDDDE[S]VKEER	S400	in
AT4G18950.1	Integrin-linked protein kinase family			84	106	[23–46]	IPEPSVH[S]EEEVFEDGEEIDGGVR	S30	in
AT4G22670.1	HSP70-interacting protein 1			55	69	[57–76]	SFVVEE[S]DDDMDETEEVKPK	S63	in
AT5G07350.1	TUDOR-SN protein 1	PM		51	105	[965–984]	IGIWQYGDIE[S]DDEDTGPAR	S975	out
AT5G44030.1	cellulose synthase A4	PM	8	68	48	[81–100]	IAGDEENNGPDD[S]DDELNIK	S93	in
AT5G49720.1	glycosyl hydrolase 9A1	PM	1	55	152	[5–24]	DPWGGPLEINTAD[S]A[T]DDDR	S18	in
								T20	in
AT5G62390.1	BCL-2-associated athanogene 7	PM		87	321	[429–446]	EIAEGVTQIVQMLE[T]EEE	T443	out
*Unknown*									
AT1G45688.1	unknown protein	PM	1	64	190	[5–19]	TDSEVTSLAAS[S]PAR	S16	out
AT3G27390.1	unknown protein	PM	5	55	219	[536–553]	DNN[S]AKDESITEPPAPVK	S539	out
AT5G64090.1	protein of unknown function	PM	1	50	131	[316–325]	[S]LEIEEDFDR	S316	out

PM SUBA, PM protein location according to the SUBA database; TMD, number of predicted trans-membrane domains according to the Aramemnon database; Mascot score, individual peptide Mascot; PTM score, post-translational modification score calculated using PhosCalc; in/out, inside/outside long IDR according to IUPforest-L predictor. Only peptides containing at least one novel and unambiguous phosphorylated residue are listed (square/normal brackets, unambiguous/ambiguous location according to the PTM score; see Materials and Methods).

The remaining 28% of the proteins consisted of 3 proteins of unknown function and 12 proteins belonging to various functional classes (Table 2). For these proteins, PM location was assumed at slightly lower rate (80%) and positive prediction of TMD concerned only nearly one half of them. Therefore, although this subset contained well-known PM proteins like the cellulose synthase A4, it is likely that part of these proteins could originate from other membrane systems, for example the golgin candidate 6, and/or have become adsorbed to PM vesicles during cell fractionation. In addition, these proteins accounted for a relatively small part of data by comparison to previous phosphoproteomics studies [12,15].

Collectively, the biochemical characterization of the membrane fraction, the function of the proteins identified and their features argue for a high content in true PM proteins in the present phosphoproteome, with prevalence for those involved in the exchange of solutes and information. In this view, the proposed strategy appears to generate information that complements available PM phosphoproteomics data. It thus enlarges by 15% the repertoire of experimentally determined phosphorylation sites in transporters and signalling proteins referenced at the PM in the PhosPhAt database (see below). Simultaneously, this dataset appears to be suitable to assess the localisation of phosphosites specifically in IDR from such proteins.

### Phosphorylation is predicted to occur by default outside of long IDR

**Figure 1:**
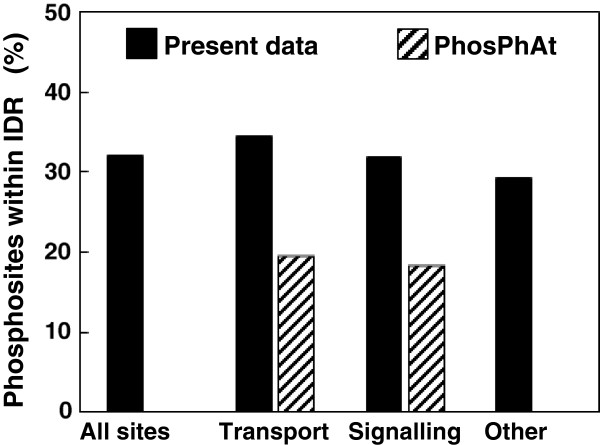
Location of phosphorylation sites inside long Intrinsically Disordered Regions (IDR), as predicted by IUPforest-L.

A number of algorithms were developed during the past decade for the prediction of IDR [25,26]. Regarding *Arabidopsis*, both pioneer estimations and recent analysis converge to a consensus of one third of proteins with a least one IDR of more than 30 residues [1,27]. In order to assess the extent of which the 75 novel phosphosites identified here could be located in such regions, we used the recent IUPforest-L predictor [28] for its high accuracy and efficiency (http://dmg.cs.rmit.edu.au/IUPforest/Eukaryota-L.php). Globally, from individual data from Table 2, the proportion of phosphorylation sites predicted to be located within long IDR amounted to 30% of the total number of phosphosites (Figure 1, black bars). This proportion held for proteins involved in transport or signalling, but was also true for proteins from other functional bins. In addition, at the phosphopeptide level, multi-phosphorylated peptides were found at a slightly lower rate (nearly 20%) within IDR than mono-phosphorylated ones. Thus, the present dataset of novel *Arabidopsis* PM phosphoproteins suggested a default prevalence of their phosphorylation sites within ordered regions, in comparison with current conclusions from combined analyses of long IDR predictions and experimental phosphosites identification in other organisms [5,20].

The origin of this unexpected finding is not clear and several causes can be envisaged. Firstly, the relatively small size of our dataset and the focus on novel sites might have introduced some bias. Accordingly, in order to average potential specific features of purified peptides, complementary information was searched by screening previously published data for other PM phosphorylated sites obtained from various methodological approaches. For this purpose, the PhosPhAt database was searched for PM proteins that are classified in the bins 30 (signalling) and 34 (transport) of the *Arabidopsis* MapMan ontology. The location of the resulting set of phosphosites, including those identified as not novel in our study (187 and 160 in signalling and transport proteins, respectively) was then checked as above, using the IUPforest-L predictor (Additional file 4). Figure 1 (hatched bars) shows that less than 20% of sites were estimated to be located within long IDR, similarly for signalling proteins and transporters. Further combining this phosphoproteomics information with data from this work resulted in a set of 398 unambiguous phosphosites belonging to signalling and transport proteins, and showing similar distribution within IDR (20% and 22%, respectively).

**Figure 2:**
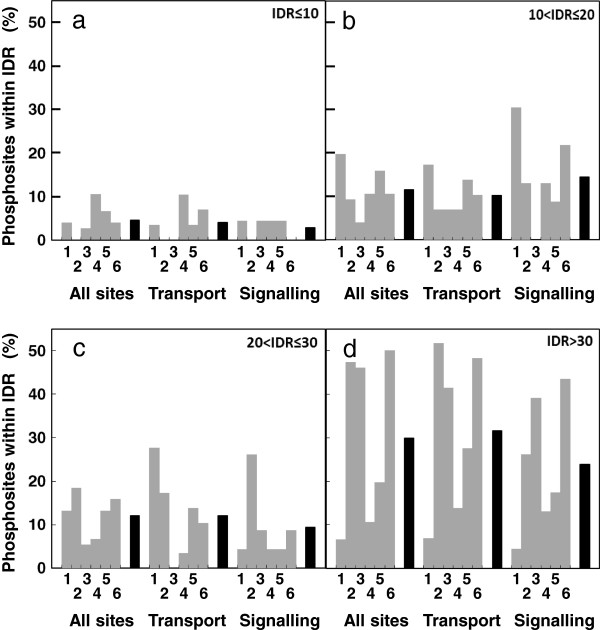
**Comparative prediction of phosphorylation sites inside long or short IDR.** Six predictors, running on the MeDor meta-server were compared: DisEMBL (1), DorA (2), FoldIndex (3), GlogPlot2 (4), IUPred (5) and RONN (6). The mean global accuracy (± SD) on all the sites for the IUPred, DisEMBL and RONN predictors were 68 ± 11%, 67 ± 12% and 68 ± 8% on a per residue basis respectively. Due to their small size, no prediction accuracy is given for the subsets “Transport” and “Signalling”. The accuracy for the 3 other predictors were not provided by the server.

Beside the nature of the dataset, another bias could arise from the prediction of IDR itself. Actually, it has been shown that predictors perform better with long IDR whom the boundary is currently established over 30 residues, than with shorter regions [29]. Large-scale statistics describing the relationship between IDR and phosphorylation rely indeed on IDR over a size of 30 residues, at the whole genome level as well as at the membrane level [1]. As the IUPforest-L predictor focuses on such IDR and ignore shorter disordered regions, we compared the results obtained for all proteins from our dataset of PM proteins with those from a consensus of 6 other predictors These predictors (DisEMBL, DorA, FoldIndex, GlobPlot2, RONN and IUPred) have complementary performances, not restricted to long IDR [30] and run simultaneously through the meta-server MeDor [31]. Figure 2 (black bars, panel d) shows that, on average, 30% of all phosphosites presented here could be proposed to be located in long IDR. However, another 28% of all phosphosites might be located in shorter regions (from 4 to 30 residues) also predicted as disordered (Figure 2, black bars, panels a, b and c). An equal proportion of about 10% was found for both intermediate sizes (11 to 20 and 21 to 30 residues) when only 5% of all phosphosites were predicted to be located in very short IDR (10 residues or less). In addition, this distribution held true for transport and signalling proteins. This situation was very predictor-dependent (Figure 2, grey bars), according to their own performance, with a proportion divergence around 40% for long IDRs and 15% for the 3 ranges described for short IDR for the total dataset. Moreover, just over 50% of phosphorylation sites were predicted to be located in regions, despite their size, simultaneously by at least 4 predictors as disordered (15% and 20% of short and long IDR, respectively, when took independently) and less than 8% by all the 6 predictors, (data not shown). Thus, together with results obtained above using IUPforest-L, this comparison might argue for a relatively limited distribution of our phosphosites set within long IDR (*ca* 30%). Simultaneously, it pointed out on a possible role of short IDRs, although the low accuracy prediction [29] did not allow raising reliable conclusion. However, a potential contribution of short IDR, despite the low consensus of most of the predictions, might reach the proportion to over 50% of phosphosites of our dataset proposed as located in disordered regions regardless of the size.

Hence, both the extension of the present dataset to published data and the combination of several algorithms propose that a large part of phosphorylation events affecting the *Arabidopsis* PM proteins could occur by default outside long unordered regions of over 30 residues. As this notion is supported by a substantial number of phosphorylation sites (close to 400), the involvement of a sampling bias is unlikely. Similarly, as phosphorylation concerns cytosolic regions of PM proteins, the membranous nature of proteins is likely not responsible for the distribution observed. Thus, this supports the conclusion derived for the dataset obtained in this work and suggests that prevalent phosphorylation outside of long IDR might constitute a previously overlooked feature in *Arabidopsis* PM, specifically with transport and signalling proteins.

### Examples of known regulatory phosphosites in *Arabidopsis* are predicted to occur mainly outside of long IDR

Whereas the functional role of most PM phosphosites above is presently unknown, for some of them a key role was demonstrated in various types of regulations (not listed in Table 2 which is restricted to novel sites). For instance, the proton pumping ATPases of the AHA family, constitute the major primary transporters that energize a number of secondary active PM transporters. They are well-known to be regulated by different phosphorylation events, notably on the conserved penultimate Threonine, which enables binding of regulatory 14-3-3 proteins and results in ATPase activation [32]. But none of the 12 AHA isoforms is predicted to display long IDR by IUPforest-L. Similarly, the activity of the AMT1:1 ammonium transporter was demonstrated to be regulated by phosphorylation of the conserved T460 in its C-terminal part [33]. But again no IDR is predicted in none of the 5 isoforms of the AMT family. Except from activity regulation through phosphorylation for different transporters, new evidence recently showed that their trafficking is also controlled by specific phosphorylation events. This is the case for instance for the PIP2:1 isoform of aquaporins, where phosphorylation of S283 was necessary for correct targeting at the PM [34]. In this case also, none of the 8 isoforms of the PIP2-type of water channels is predicted to display IDR. However, more complex situations can be noticed for other transporters. In the case of phosphate transporters, the S514 residue of the PHT1;1 isoform is conserved in 7 out the 9 isoforms of the family and its phosphorylation was shown to prevent the PHT1:1 isoform from reaching its correct PM destination [35]. For this family, no long IDR is predicted in 6 isoforms, including PHT1:1. However the Serine residue homologue to S514 is predicted to be located within a long IDR in 2 out the 3 other isoforms. For signalling proteins, that constitute the other main class characterized here, less data is available to assess the relationship between regulatory phosphorylation events and disorder. A notable exception concerns the BRI1/BAK1 complex, involving LRR-RLK that trans-phosphorylate each other [36]. For this complex, the activation or inhibitory role of respectively 12 and 5 phosphosites was demonstrated in BRI1 and BAK1, several other sites being identified but not characterized functionally. However, no IDR is predicted for BRI1 or for BAK1.

Thus, with some exceptions, the examples described above seem to illustrate well the functional aspect of the results obtained from the predictive analysis of a larger set. Thus the location of phosphorylation events affecting crucial functional features (such as activity, membrane targeting or protein interaction) does not appear associated with long disordered regions for the regulation of most of these transport and signalling proteins at *Arabidopsis* PM. The specific characteristics of such proteins did not allow extrapolating this conclusion to other proteins from other functional categories and with other physicochemical properties.

## Conclusions

The phosphoproteomics data of plant plasma membrane proteins presented here, along with published datasets and available functional information, suggested a preferred topology of phosphorylation, at least regarding transport and signalling functions. This finding was unexpected according to the relationship of phosphorylation with long IDR usually reported, even after taking into account the questionable contribution of predicted short IDRs in our data set. Although the significance of this situation remains to be elucidated, two alternative or complementary hypotheses may be proposed. As about one half of *Arabidopsis* PM phosphosites would be located outside predicted disordered regions in proteins, it might be speculated that such observed phosphosites would have a more constitutive role rather than a regulatory role. The significance of such a role should be investigated in detail. Simultaneously, a part of regulatory phosphorylation events could be assumed to concern flexible, but of short length and more difficult to accurately predict, regions of proteins.

## Methods

### Samples

*Arabidopsis* (ecotype Col-0) suspension cells were grown in liquid Murashige and Skoog medium and ground in homogenisation buffer (100 mM Tris/HCl pH 8, 0.5 M sucrose, 10% glycerol (w/v), 0.6 % PVP (w/v), 10 mM EDTA, 10 mM EGTA, 10 mM ascorbic acid, 5 mM DTT, 1 mM PMSF, 1 μg/mL leupeptine) supplemented with, 1 mM sodium molybdate, 1 mM orthovanadate, 50 mM sodium fluoride, 10 mM sodium pyrophosphate and 10 mM glycerophosphate to prevent protein dephosphorylation (as described elsewhere [16]). A crude membrane fraction was obtained by differential centrifugation (10 000 *g*_max_, 80 000 *g*_max_) and PM vesicles were extracted by two-phases partitioning using 6.4 % polyethylene glycol and dextran as in [37]. Purified vesicles were treated with 0.01% Brij58 to promote inside-out sidedness [38] and proteins (500 μg) were digested using trypsin (1/50, w/w; 37°C overnight), to obtain peptides from the cytoplasmic side.

### Peptide purification

Strong Anion eXchange (SAX) microcolumns (packed in GELoader tips and equilibrated with 25 mM ammonium formate pH 7.5 and 30% acetonitrile) were used to fractionate PM peptides using increasing concentration of ammonium formate (6 steps from 25 mM to 1 M). Fractions were concentrated *in vacuo* to approximately 2 μL and diluted to 30 μL with 5% TFA in 80% acetonitrile prior to phosphopeptide selection. After loading on TiO_2_ microcolumns (prepared as in [39]) and washing with 1% TFA in 80% acetonitrile, phosphopeptides were eluted with 0.5% and 4.5% ammonium hydroxide.

### Mass spectrometry and data analysis

Peptides were analyzed on an ion-trap Esquire HCT-plus mass spectrometer (Bruker) coupled to a ChipCube HPLC (Agilent). The chip contained both the pre-column and the column filled with the same stationary phase (Zorbax 300SB-C18; Agilent). Samples were first loaded onto the 4 mm enrichment pre-column at a flow rate of 4 μL/min using solvent A (0.1% formic acid). After pre-concentration, peptides were separated on the column (75 μm diameter, 150 mm length) at a flow rate of 0.3 μL/min using a 30 min linear gradient from 3% to 45% solvent B (0.1% formic acid, 90% acetonitrile) and eluted into the mass spectrometer. Raw MS data were processed using DataAnalysis and BioTools softwares (Bruker) to centroid spectra before querying the *Arabidopsis* TAIR9 database (http://www.arabidopsis.org/; version pep_20090619) in the target-decoy mode using the Mascot search engine (Matrix Science; version 2.2.04). The following search parameters were used: up to one missed trypsin cleavage allowed, 1.2 Da mass tolerance for MS and 0.9 Da for MS/MS fragment ions; phosphorylation (ST) and (Y) as variable modifications. Under these conditions, for the dataset generated, a Mascot peptide score above 46 corresponded to 1% false discovery rate (FDR). For positive phosphopeptides, the probability based PTM score was calculated using the stand-alone software PhosCalc [22] including the algorithm developed by Olsen *et al.*[21], in order to assign individual phosphorylation sites. When different locations were computed, only those whose the score was higher than the maximum score minus five, were taken as unambiguous [21] and all others were rejected.

## Abbreviations

IDR: Intrinsically disordered regions; PM: Plasma membrane; TMD: Trans-membrane domain.

## Competing interests

The authors declare that they have no competing interests.

## Authors’ contributions

CN performed both experimental steps upstream to mass spectrometry and bioinformatics analysis and wrote the paper. VR performed mass spectrometry experiments under the supervision of NS. SH analyzed phosphorylation data. MR designed the work and revised the manuscript. All authors read and approved the final manuscript.

## Supplementary Material

Additional file 1 **Figure S1.** Work-flow for the identification of novel phosphorylation sites in *Arabidopsis* plasma membrane. Click here for file

Additional file 2 Fragmentation data of novel and unambiguous phosphopeptides and sites. Click here for file

Additional file 3 **Figure S2.** Phosphohydrolase activities of the membrane fraction. Click here for file

Additional file 4 **Phosphorylated proteins, peptides and sites from the bins 30 and 34 of the PhosPhAt data base.** in/out, inside/outside long IDR according to IUPforest-L predictor. Only peptides containing at least one unambiguous phosphorylated residue are listed. Click here for file
