# Supplementary material for: Phosphoproteomic analysis reveals major default phosphorylation sites outside long intrinsically disordered regions of Arabidopsis plasma membrane proteins
Source: Proteome Sci. 2012 Oct 30;10:62. doi: 10.1186/1477-5956-10-62 (PMC3537754; doi:10.1186/1477-5956-10-62)
Supplement: Additional file 1 — Figure S1. Work-flow for the identification of novel phosphorylation sites in Arabidopsis plasma membrane. [file 1477-5956-10-62-S1.pdf]

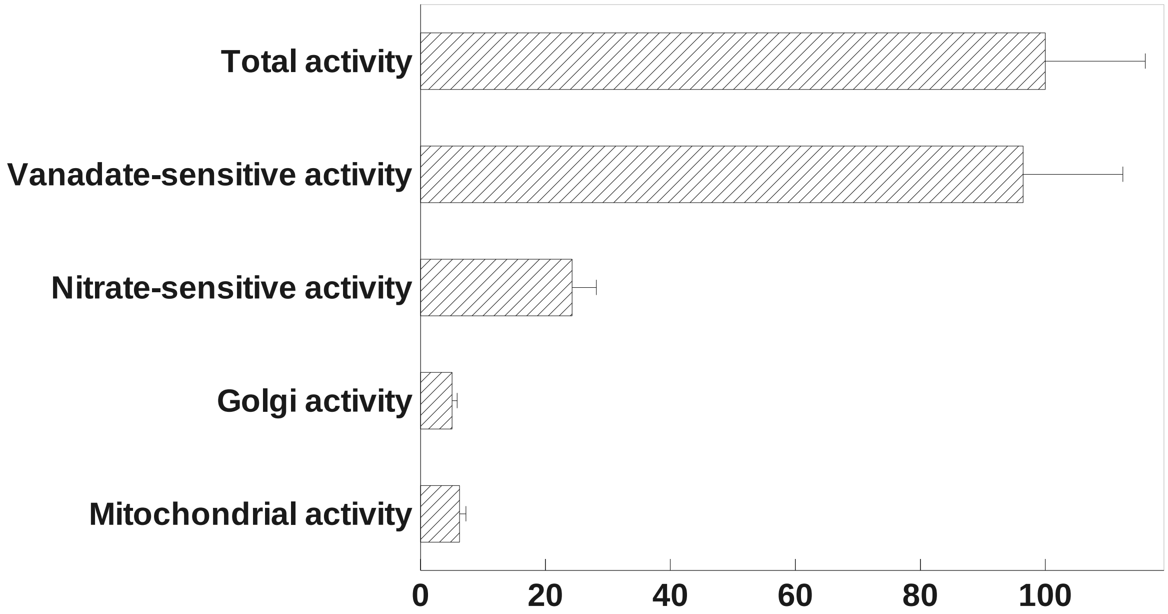

Supplementary Figure S1. Phosphohydrolase activities of the membrane fraction. Assays were performed at 38°C for 30 min in MES/Tris buffer (pH 6.5) containing 50 mM KCl, 3 mM $MgSO_4$, 3 mM ATP and 0.025 mg/mL lysolecithin. Total activity amounted to 5.97 µmol Pi liberated $min^{-1}.mg^{-1}$. The vanadate-sensitive activity (plasma membrane H^+-ATPase activity was measured in the presence of 100 µM ortho-vanadate. For the nitrate-sensitive activity (vacuolar membrane ATPase), KNO_3 was used in place of KCl. The IDPase activity (Golgi apparatus) was mesured by replacing ATP by IDP. The sensitivity to 100 µM NaN_3 was used to estimate the presence of mitochondrial membranes.
