# Supplementary material for: Phosphoproteomic analysis reveals major default phosphorylation sites outside long intrinsically disordered regions of Arabidopsis plasma membrane proteins
Source: Proteome Sci. 2012 Oct 30;10:62. doi: 10.1186/1477-5956-10-62 (PMC3537754; doi:10.1186/1477-5956-10-62)

Annotated spectra report

(pS): phosphoserine
(pT): phosphothreonine
(pY): phosphotyrosine
(s), (t), (y): ambiguous phosphorylation site

Transporters

VE(pS)DVV(pS)LDGHDFLETDAQIGDDGK

MS/MS Fragmentation of **VESDVVSLDGHDFLETDAQIGDDGK**
Found in **AT1G23080.1**, | Symbols: PIN7, ATPIN7 | PIN7 (PIN-FORMED 7); auxin efflux transmembrane transporter/ auxin:hydrogen symporter/ transporter | chr1:8180768-8183406 REVERSE

Match to Query 556: 2820.908172 from(941.310000,3+) intensity(155215.0000)
Title: Cmpd 79, +MSn(941.9), 22.6 min

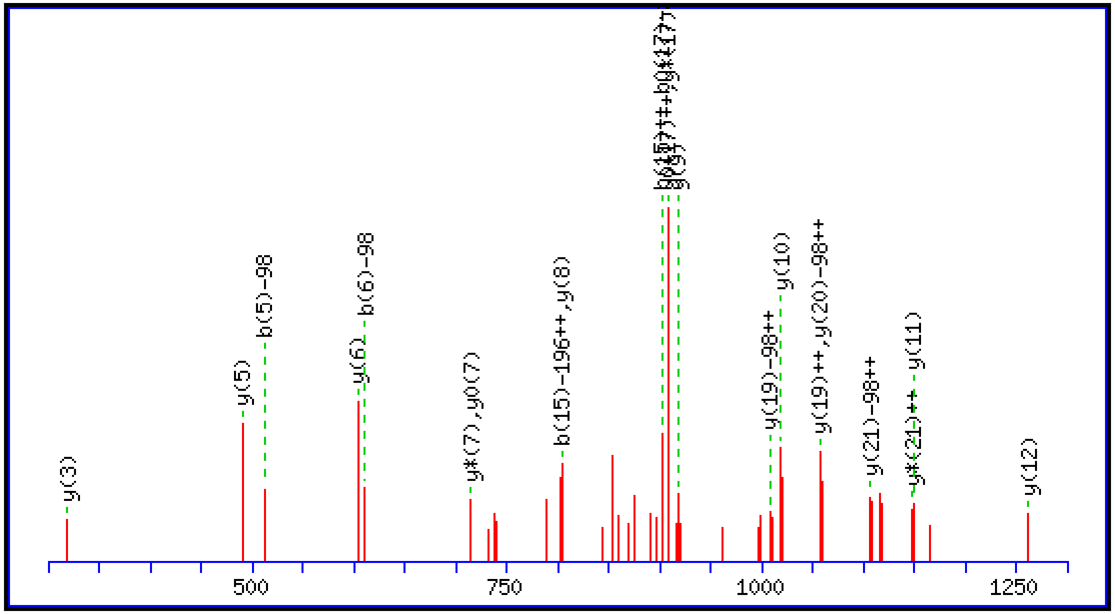

V(pS)(pT)PEIL(pT)PSGQR

MS/MS Fragmentation of **VSTPEILTPSGQR**

Found in **AT1G47670.1**, | Symbols: | amino acid transporter family protein | chr1:17536834-17539486 REVERSE

Match to Query 297: 1624.005448 from(813.010000,2+) intensity(253210.0000)

Title: Cmpd 31, +MSn(813.4), 13.8 min

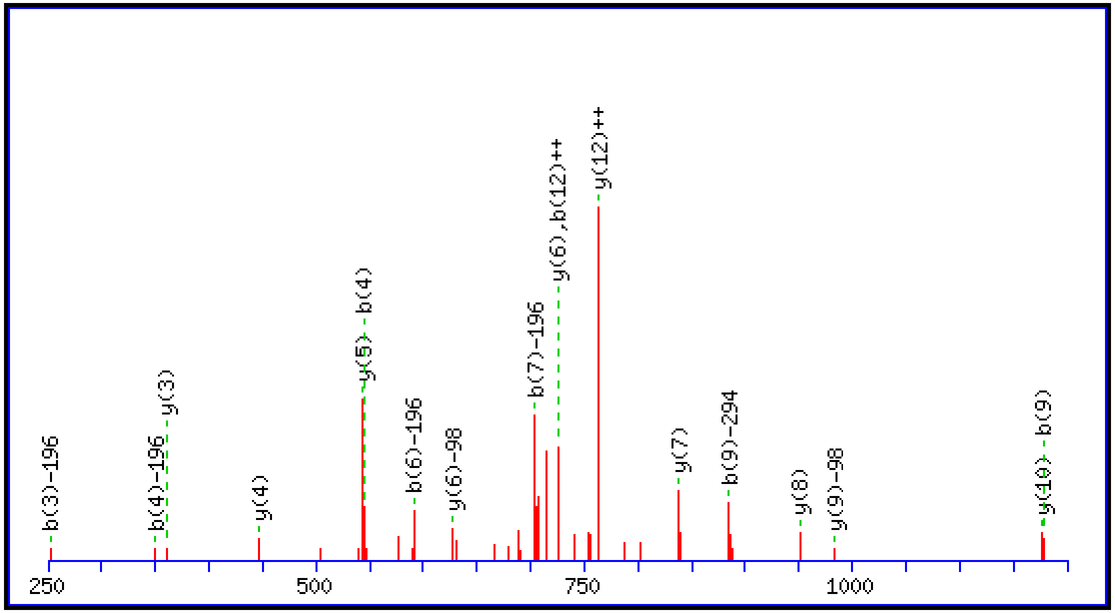

EEQEE(pS)VEGIFESR

MS/MS Fragmentation of **EEQEESVEGIFESR**
Found in **AT1G48370.1**, | Symbols: YSL8 | YSL8 (YELLOW STRIPE LIKE 8); oligopeptide transporter | chr1:17874560-17877256 FORWARD

Match to Query 402: 1747.005448 from(874.510000,2+) intensity(170740.0000)
Title: Cmpd 75, +MSn(875.0), 19.3 min

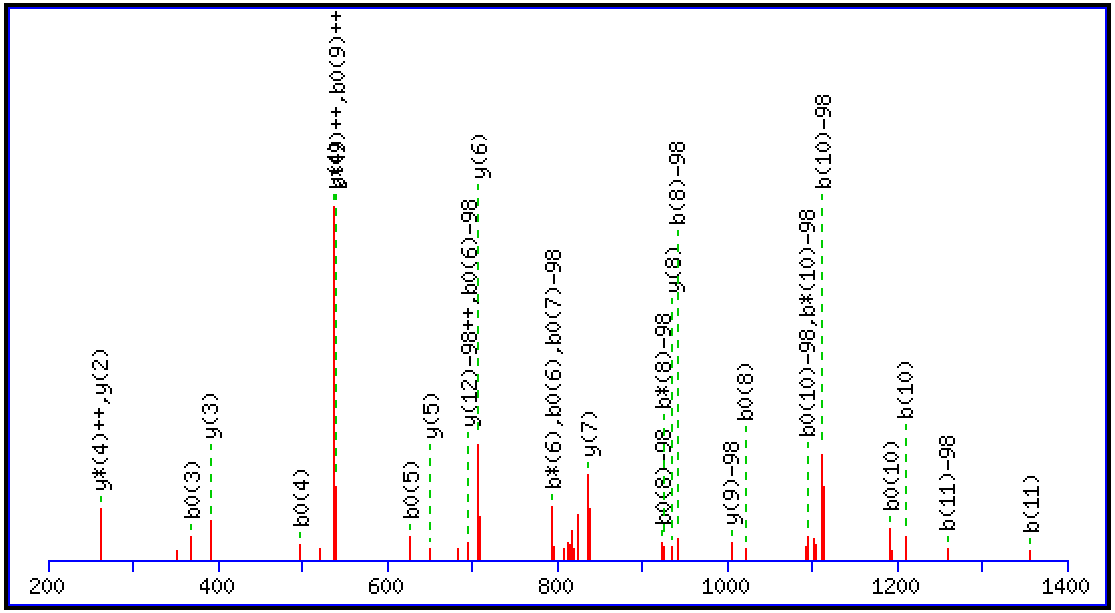

SLEENEDEIVST(s)AG(s)(pS)PANELLR

MS/MS Fragmentation of **SLEENEDEIVSTSAGSSPANELLR**
Found in **AT1G76430.1**, | Symbols: | phosphate transporter family protein |
chr1:28679115-28681874 REVERSE

Match to Query 458: 2706.668172 from(903.230000,3+) intensity(116762.0000)
Title: Cmpd 62, +MSn(903.7), 20.0 min

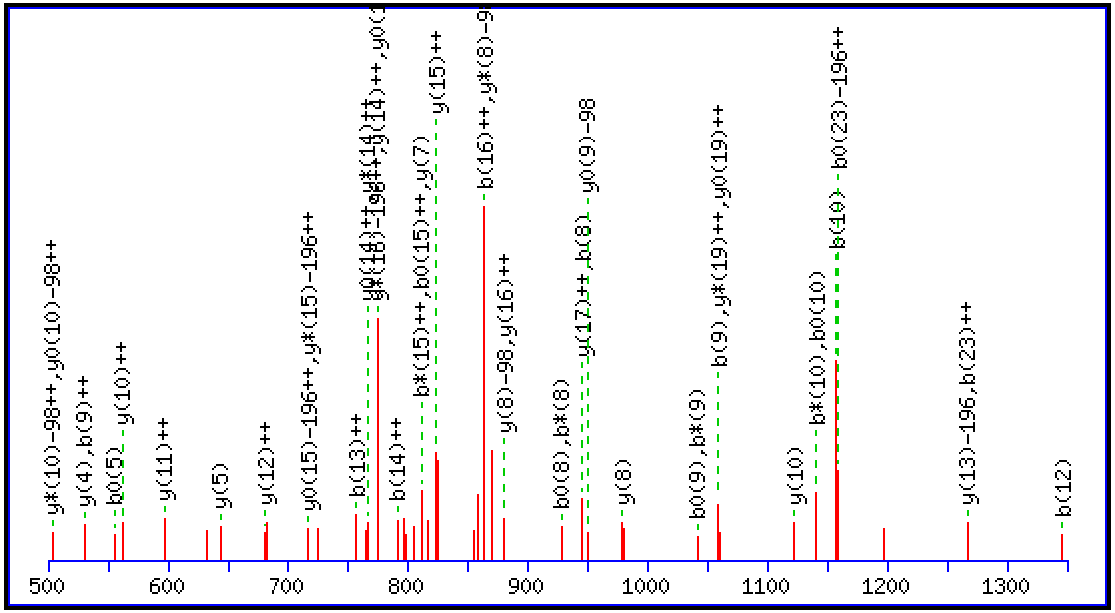

VE(pS)DVV(pS)LDGHDFLETDAEIGNDGK

MS/MS Fragmentation of **VESDVVSLDGHDFLETDAEIGNDGK**

Found in **AT2G01420.1**, | Symbols: PIN4, ATPIN4 | PIN4 (PIN-FORMED 4); auxin:hydrogen symporter/ transporter | chr2:180478-183199 REVERSE

Match to Query 319: 2820.908172 from(941.310000,3+) intensity(155215.0000)

Title: Cmpd 79, +MSn(941.9), 22.6 min

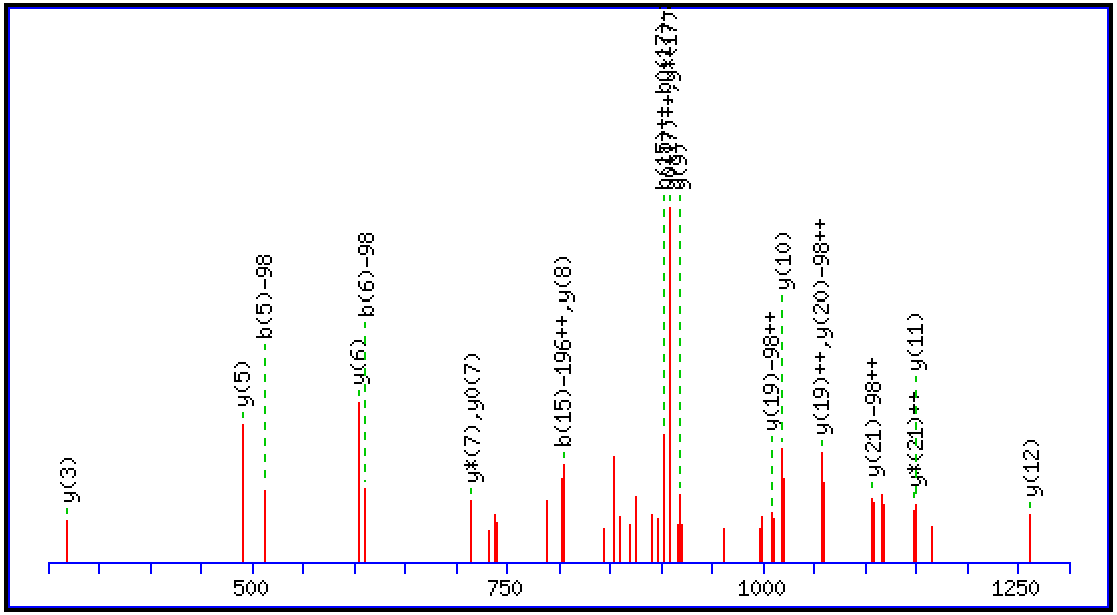

QNTMVE(pS)(pS)DEEDEDEGIVVR

MS/MS Fragmentation of **QNTMVESSDEEDEDEGIVVR**

Found in **AT2G01980.1**, | Symbols: SOS1, ATSOS1, ATNHX7 | SOS1 (SALT OVERLY SENSITIVE 1); sodium:hydrogen antiporter | chr2:457070-463145 FORWARD

Match to Query 303: 2441.288172 from(814.770000,3+) intensity(366367.0000)

Title: Cmpd 10, +MSn(815.2), 16.0 min

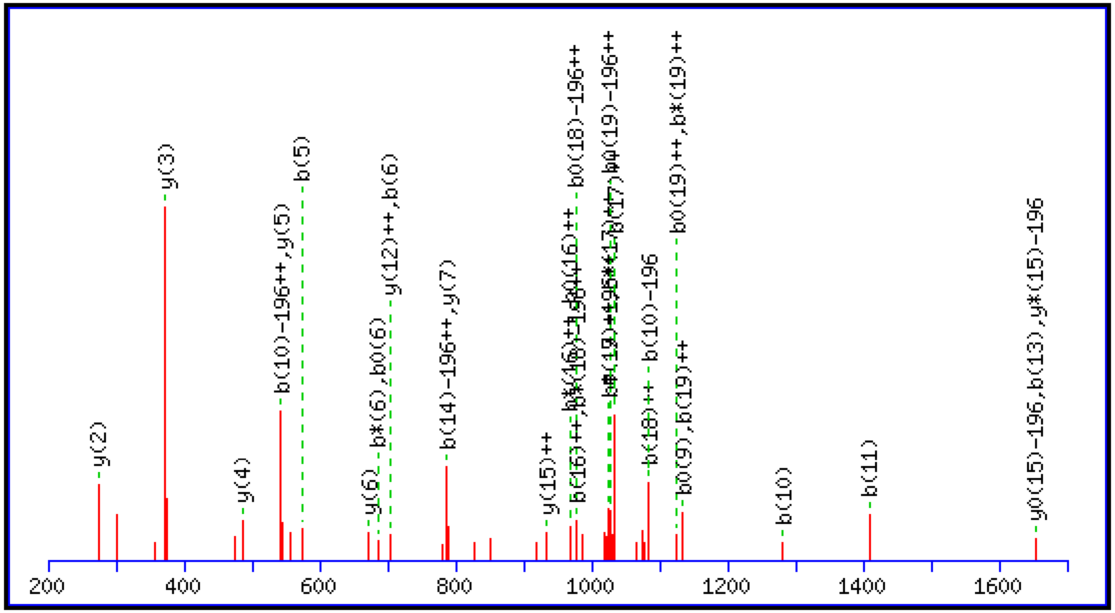

QPISFED(pS)PEWED(pT)PDVDLR

MS/MS Fragmentation of **QPISFEDSPEWEDTPDVDLR**

Found in **AT2G28070.1**, | Symbols: | ABC transporter family protein | chr2:11956432-11959782 FORWARD

Match to Query 355: 2534.528172 from(845.850000,3+) intensity(82891.0000)

Title: Cmpd 70, +MSn(846.2), 21.2 min

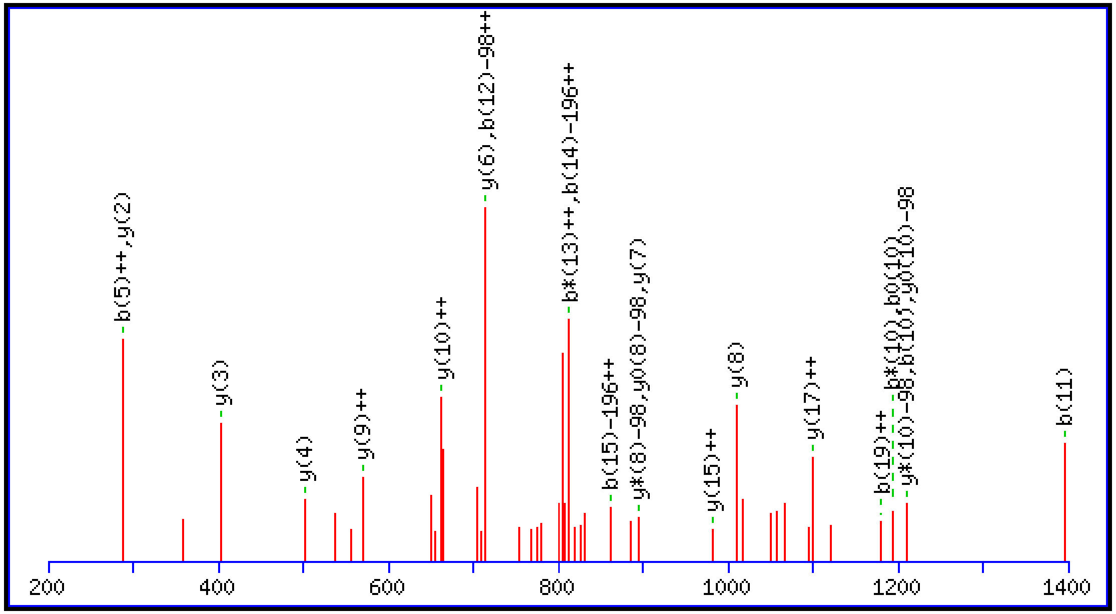

E(pS)PESESELVPDSR

MS/MS Fragmentation of **ESPESESELVPDSR**

Found in **AT2G28120.1**, | Symbols: | nodulin family protein | chr2:11985934-11987667 FORWARD

Match to Query 313: 1640.045448 from(821.030000,2+) intensity(1027849.0000)

Title: Cmpd 21, +MSn(821.4), 15.4 min

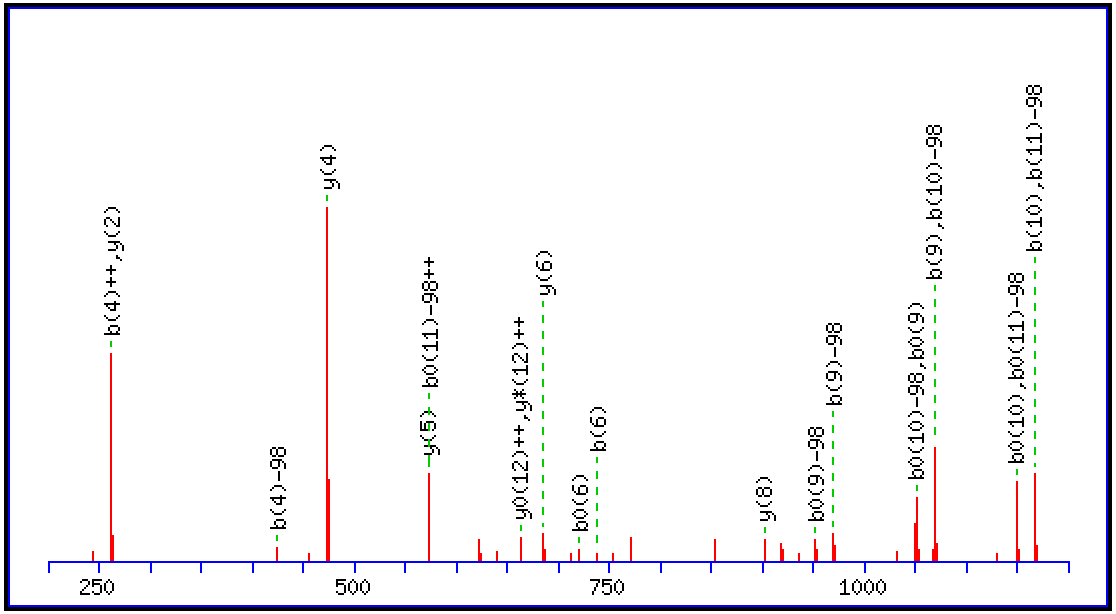

EDEEQSGGD(pT)VVEMTVANSGR

MS/MS Fragmentation of **EDEEQSGGDTVVEMTVANSGR**
Found in **AT2G32830.1**, | Symbols: PHT5 | PHT5; inorganic phosphate transmembrane transporter/ phosphate transmembrane transporter | chr2:13927754-13929657 REVERSE

Match to Query 242: 2289.698172 from(764.240000,3+) intensity(78590.0000)
Title: Cmpd 53, +MSn(764.6), 18.3 min

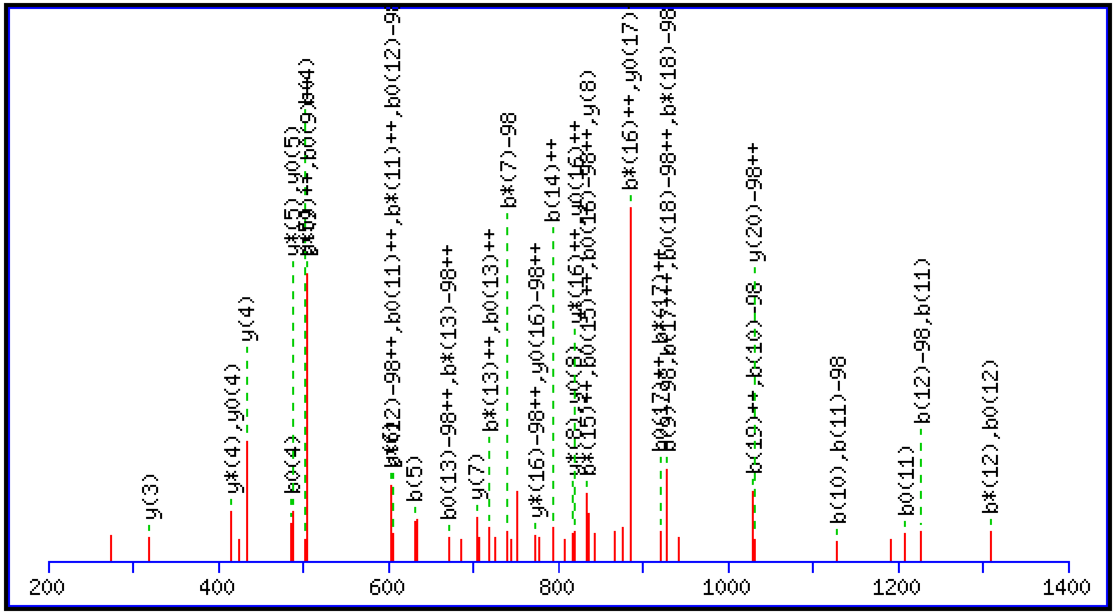

(s)NG(s)EPE(pS)PVSPLLTSDPK

MS/MS Fragmentation of **SNGSEPESPVSPLLTSDPK**

Found in **AT3G55320.1**, | Symbols: PGP20 | PGP20 (P-GLYCOPROTEIN 20); ATPase, coupled to transmembrane movement of substances | chr3:20507391-20513393 REVERSE

Match to Query 673: 2099.965448 from(1050.990000,2+) intensity(219513.0000)

Title: Cmpd 97, +MSn(1051.5), 17.3 min

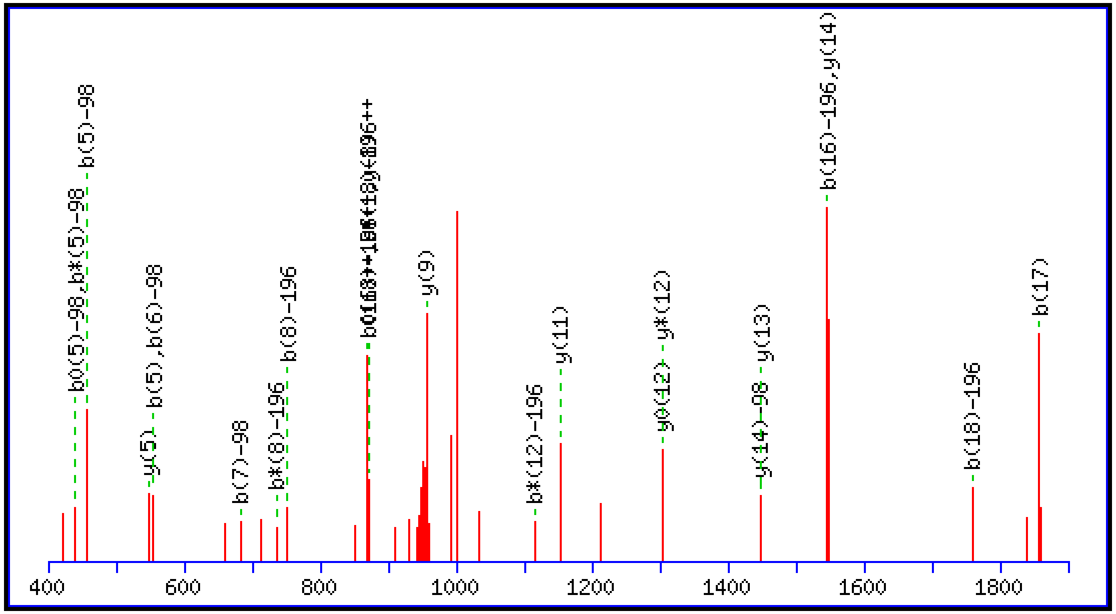

NVTTEESLVEDSE(pS)P

MS/MS Fragmentation of NVTTEESLVEDSESP
Found in AT4G23700.1, | Symbols: ATCHX17, CHX17 | ATCHX17 (CATION/H+ EXCHANGER 17); monovalent cation:proton antiporter/ sodium:hydrogen antiporter | chr4:12342534-12345616 REVERSE

Match to Query 377: 1715.185448 from(858.600000,2+) intensity(356474.0000)
Title: Cmpd 15, +MSn(858.8), 16.1 min

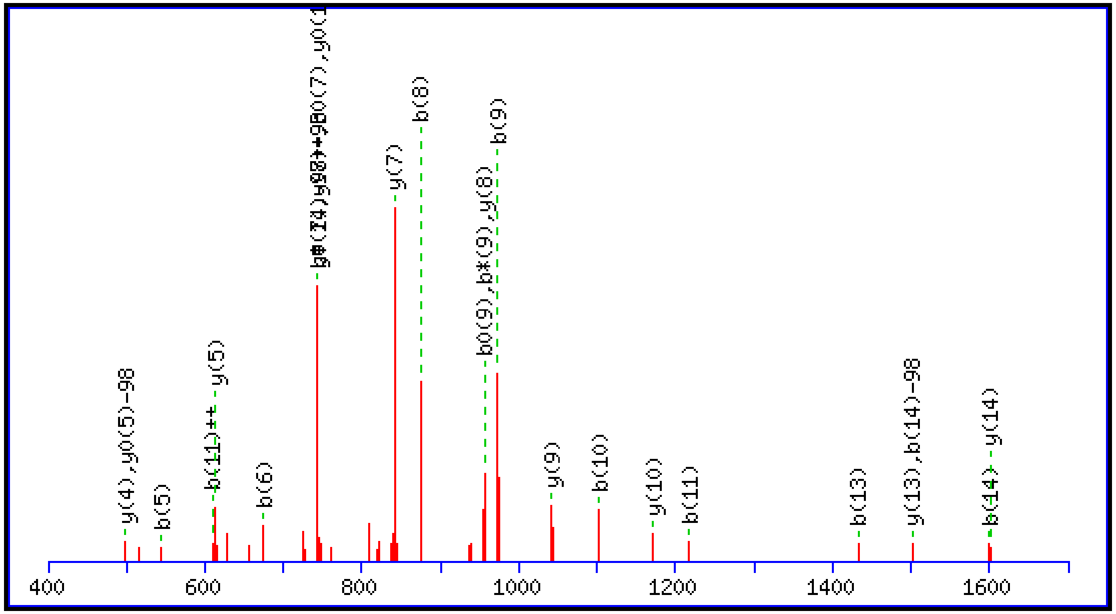

NVTTEESLVED(pS)E(pS)P

MS/MS Fragmentation of **NVTTEESLVEDSESP**
Found in **AT4G23700.1**, | Symbols: ATCHX17, CHX17 | ATCHX17 (CATION/H+ EXCHANGER 17); monovalent cation:proton antiporter/ sodium:hydrogen antiporter | chr4:12342534-12345616 REVERSE

Match to Query 446: 1795.045448 from(898.530000,2+) intensity(237238.0000)
Title: Cmpd 7, +MSn(898.9), 15.0 min

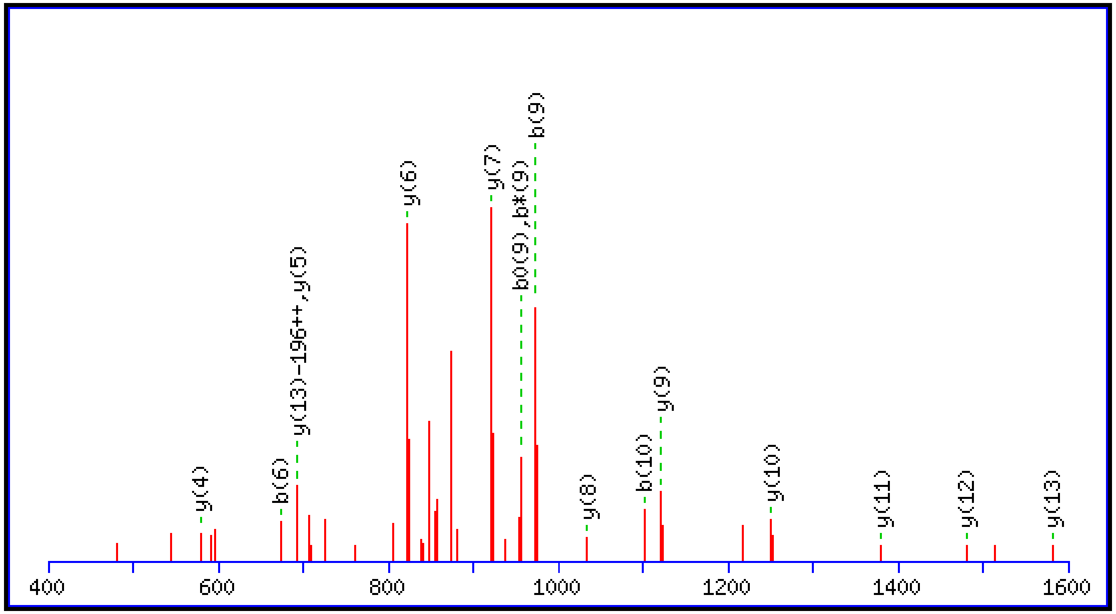

EGEEEEDNNQLSLQEEEPD(pT)EEEMSGR

MS/MS Fragmentation of **EGEEEEDNNQLSLQEEEPDTEEEMSGR**

Found in **AT4G24120.1**, | Symbols: YSL1, ATYSL1 | YSL1 (YELLOW STRIPE LIKE 1); oligopeptide transporter | chr4:12524581-12527023 FORWARD

Match to Query 690: 3232.688172 from(1078.570000,3+) intensity(1193502.0000)

Title: Cmpd 7, +MSn(1078.8), 16.6 min

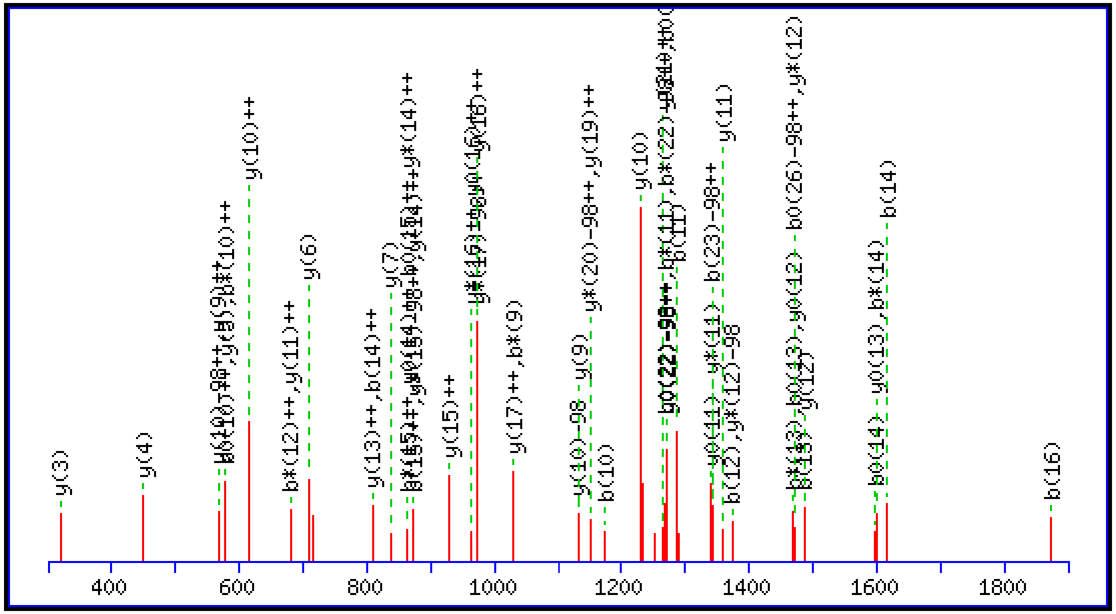

DVEAGTS(pS)FTEYEDSPFDIASTK

MS/MS Fragmentation of **DVEAGTSSFTEYEDSPFDIASTK**
Found in **AT4G29900.1**, | Symbols: ACA10, CIF1, ATACA10 | ACA10 (AUTOINHIBITED CA(2+)-ATPASE 10); calcium-transporting ATPase/ calmodulin bindin | chr4:14611225-14618775 REVERSE

Match to Query 744: 2575.105448 from(1288.560000,2+) intensity(103459.0000)
Title: Cmpd 92, +MSn(1289.0), 22.4 min

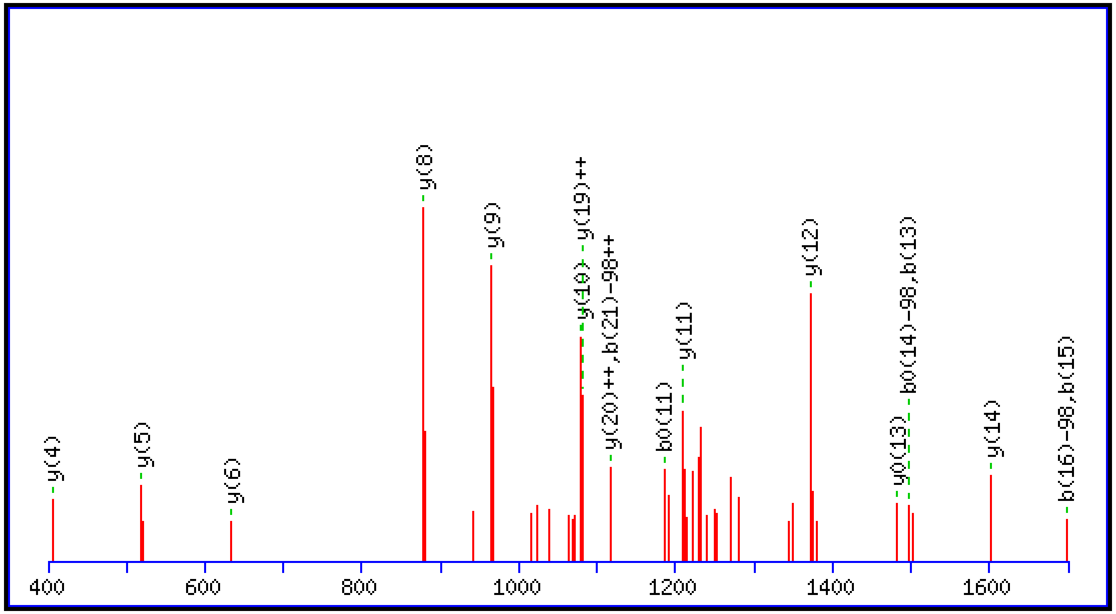

QAEESIVV(pS)GEDEVAGR

MS/MS Fragmentation of **QAEESIVVSGEDEVAGR**

Found in **AT5G01240.1**, | Symbols: | amino acid permease, putative | chr5:98228-101493 FORWARD

Match to Query 532: 1854.205448 from(928.110000,2+) intensity(420244.0000)

Title: Cmpd 42, +MSn(928.4), 16.7 min

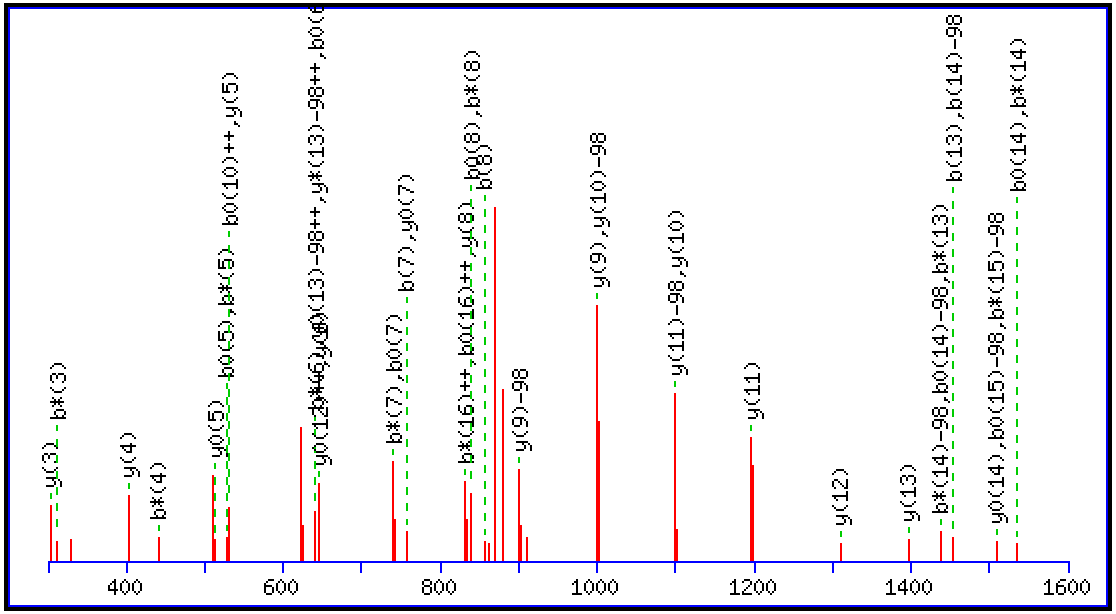

VED(pS)AAEEDIDGNGGNGFSMK

MS/MS Fragmentation of **VEDSAAEEDIDGNGGNGFSMK**

Found in **AT5G01240.1**, | Symbols: | amino acid permease, putative | chr5:98228-101493 FORWARD

Match to Query 720: 2221.965448 from(1111.990000,2+) intensity(223468.0000)

Title: Cmpd 47, +MSn(1112.4), 17.8 min

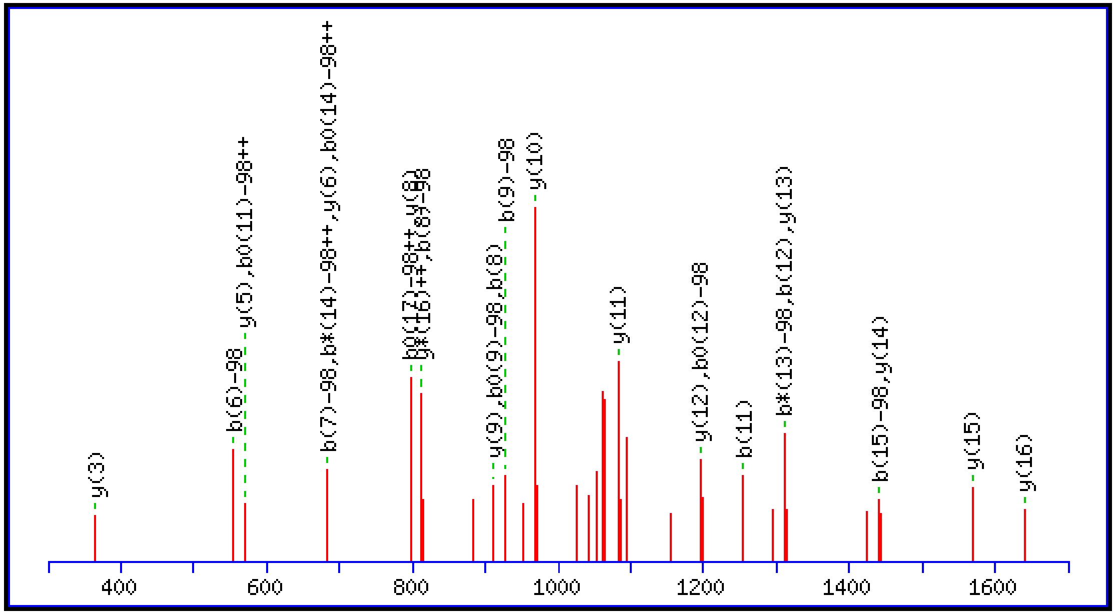

NV(pS)SENIENYLK

MS/MS Fragmentation of **NVSSENIENYLK**
Found in **AT5G24030.1**, | Symbols: SLAH3 | SLAH3 (SLAC1 HOMOLOGUE 3); transporter | chr5:8118618-8120993 REVERSE

Match to Query 217: 1489.005448 from(745.510000,2+) intensity(190434.0000)
Title: Cmpd 134, +MSn(745.9), 20.6 min

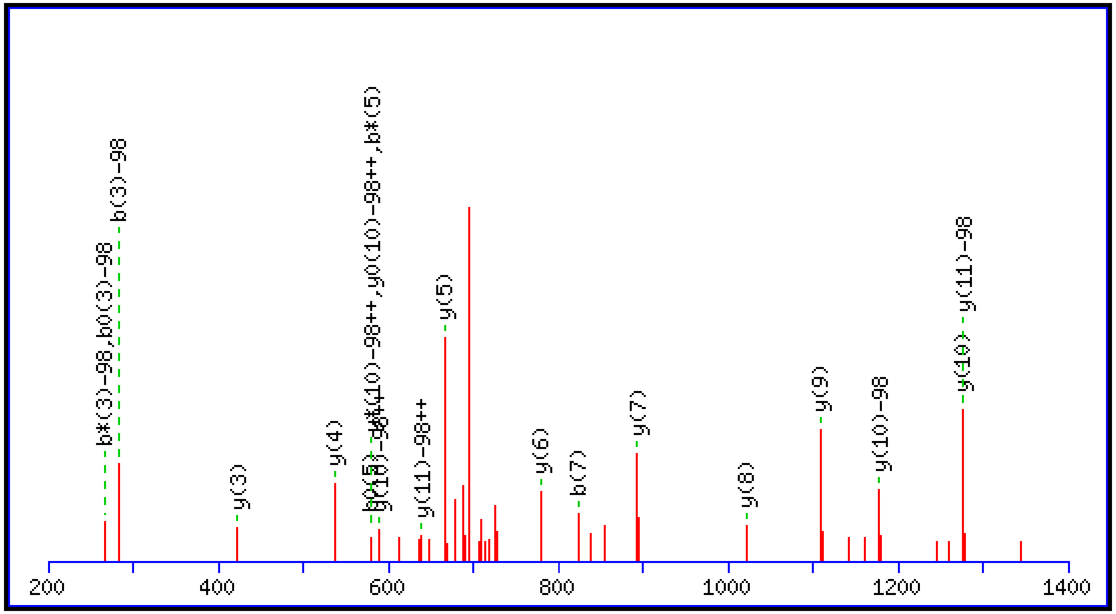

VLQ(pT)DIELEER

MS/MS Fragmentation of **VLQTDIELEER**
Found in **AT5G43350.1**, | Symbols: ATPT1, PHT1;1 | PHT1;1 (PHOSPHATE TRANSPORTER 1;1); carbohydrate transmembrane transporter/ inorganic phosphate transmembrane transporter/ phosphate transmembrane transporter/ sugar:hydrogen symporter chr5:17399918-17401643 REVERSE

Match to Query 176: 1424.025448 from(713.020000,2+) intensity(145132.0000)
Title: Cmpd 73, +MSn(713.4), 19.8 min

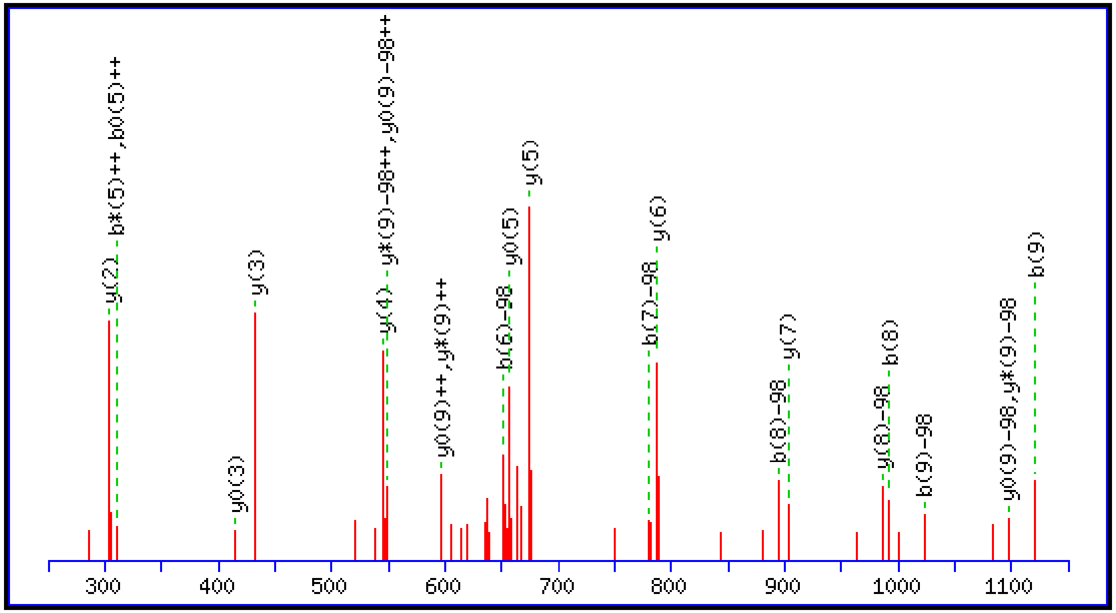

SLEELSGEAEV(pS)HDEK

MS/MS Fragmentation of **SLEELSGEAEVSHDEK**
Found in **AT5G43350.1**, | Symbols: ATPT1, PHT1;1 | PHT1;1 (PHOSPHATE TRANSPORTER 1;1); carbohydrate transmembrane transporter/ inorganic phosphate transmembrane transporter/ phosphate transmembrane transporter/ sugar:hydrogen symporter chr5:17399918-17401643 REVERSE

Match to Query 503: 1838.005448 from(920.010000,2+) intensity(145160.0000)
Title: Cmpd 47, +MSn(920.7), 21.3 min

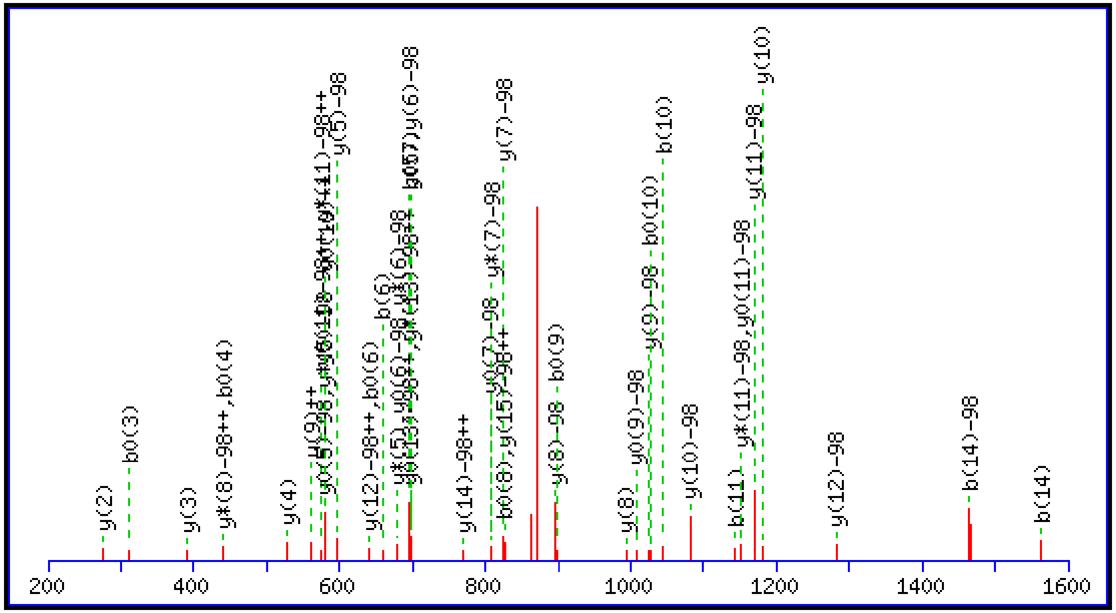

(pS)LEELSGEAEV(pS)HDEK

MS/MS Fragmentation of **SLEELSGEAEVSHDEK**
Found in **AT5G43350.1**, | Symbols: ATPT1, PHT1;1 | PHT1;1 (PHOSPHATE TRANSPORTER 1;1); carbohydrate transmembrane transporter/ inorganic phosphate transmembrane transporter/ phosphate transmembrane transporter/ sugar:hydrogen symporter chr5:17399918-17401643 REVERSE

Match to Query 599: 1918.025448 from(960.020000,2+) intensity(148421.0000)
Title: Cmpd 12, +MSn(960.3), 14.3 min

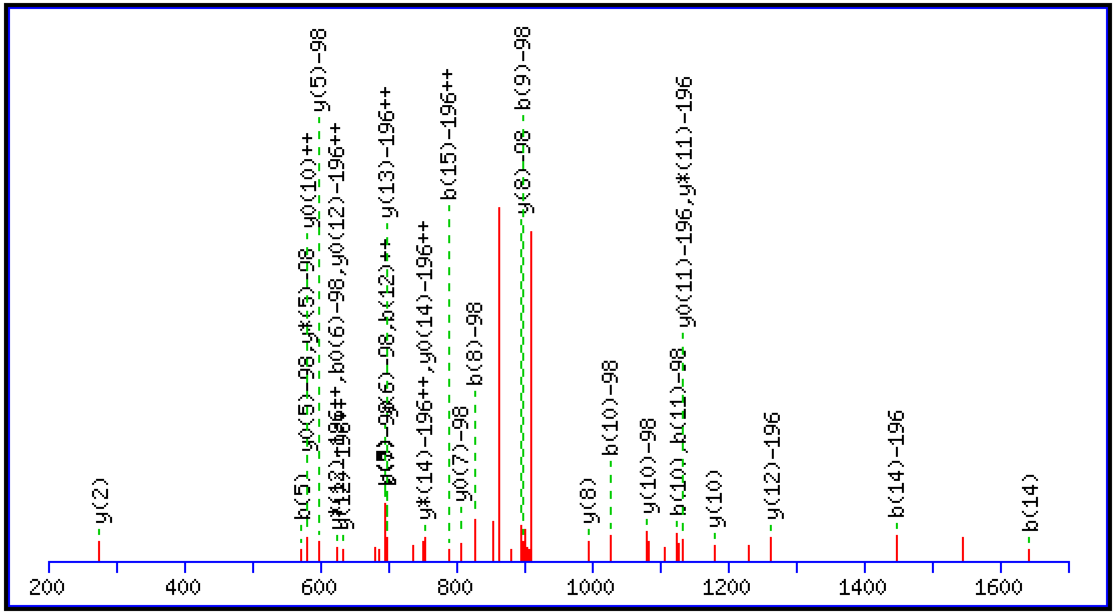

VVEA(pY)ASGDEDVDVPAEELR

MS/MS Fragmentation of **VVEAYASGDEDVDVPAEELR**

Found in **AT5G45380.1**, | Symbols: ATDUR3, DUR3 | sodium:solute symporter family protein | chr5:18391337-18395696 FORWARD

Match to Query 726: 2242.305448 from(1122.160000,2+) intensity(768325.0000)

Title: Cmpd 39, +MSn(1122.6), 20.2 min

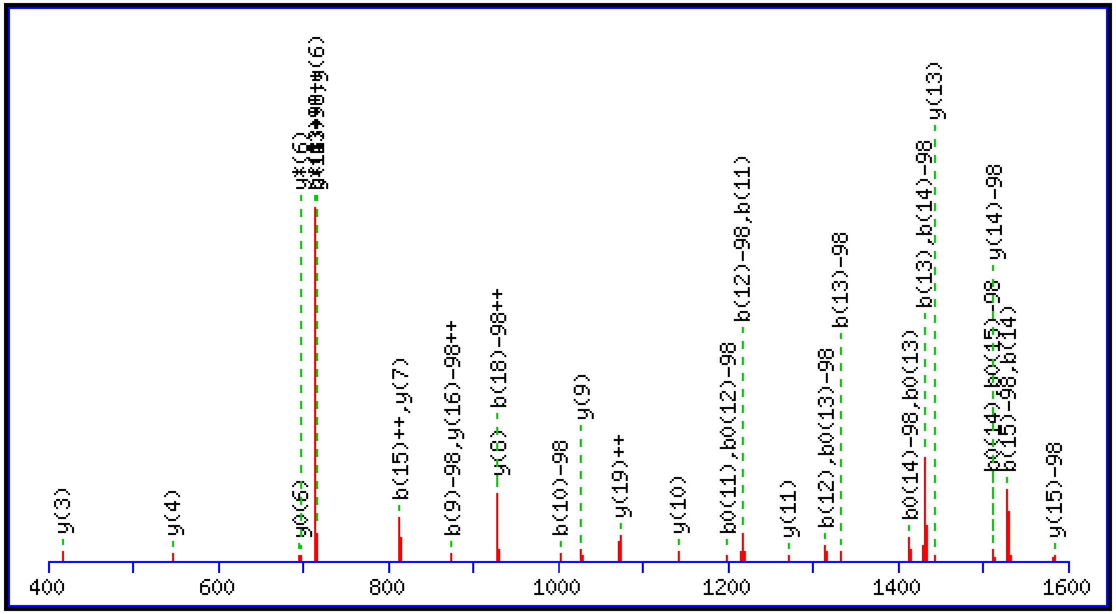

ATADEF(pS)DEDTSPIEEVR

MS/MS Fragmentation of ATADEFSDEDTSPIEEVR
Found in AT5G64410.1, | Symbols: ATOPT4, OPT4 | OPT4 (OLIGOPEPTIDE TRANSPORTER 4); oligopeptide transporter | chr5:25750921-25754974 REVERSE

Match to Query 670: 2090.125448 from(1046.070000,2+) intensity(239218.0000)
Title: Cmpd 33, +MSn(1046.5), 18.9 min

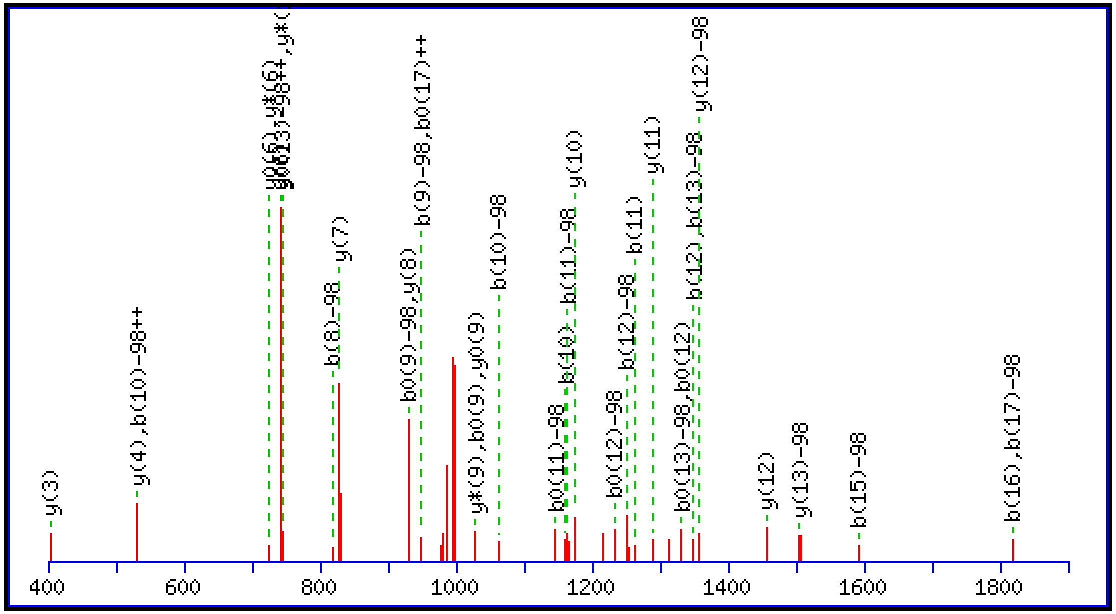

EIAGAQNDGD(pT)GDEDESPFEFR

MS/MS Fragmentation of **EIAGAQNDGDTGDEDESPFEFR**
Found in **AT5G64560.1**, | Symbols: | magnesium transporter CorA-like family protein (MRS2-2) | chr5:25807025-25809395 REVERSE

Match to Query 336: 2478.638172 from(827.220000,3+) intensity(91090.0000)
Title: Cmpd 85, +MSn(827.7), 20.1 min

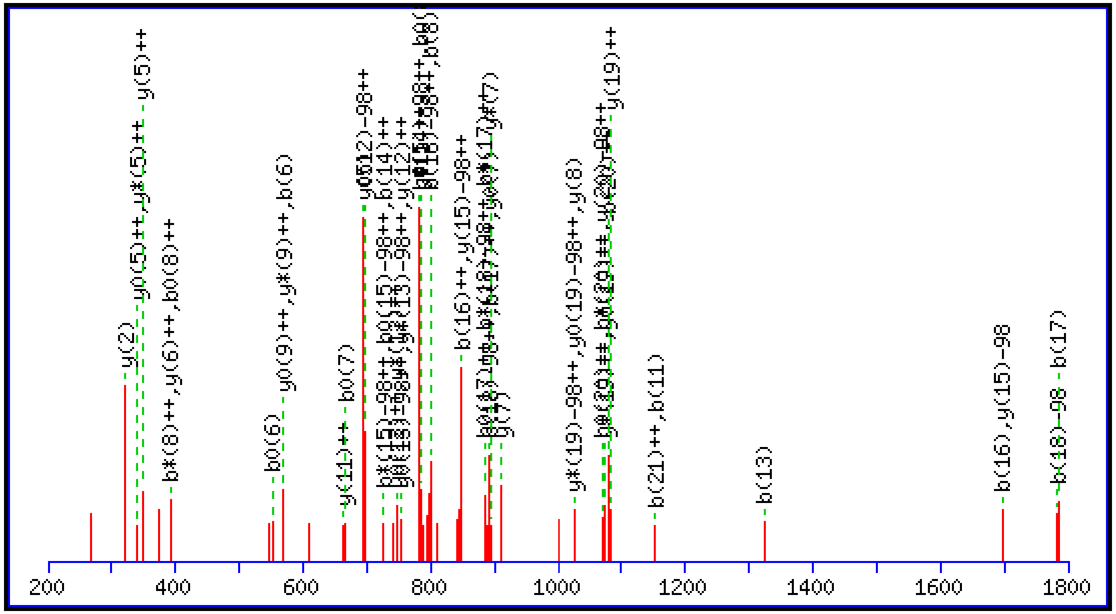

Signaling

ADNNNNNVDAFSDAGW(pS)R

MS/MS Fragmentation of **ADNNNNNVDAFSDAGWSR**
Found in **AT1G05150.1**, | Symbols: | calcium-binding EF hand family protein |
chr1:1484280-1486706 REVERSE

Match to Query 659: 2045.885448 from(1023.950000,2+) intensity(140365.0000)
Title: Cmpd 46, +MSn(1024.5), 19.7 min

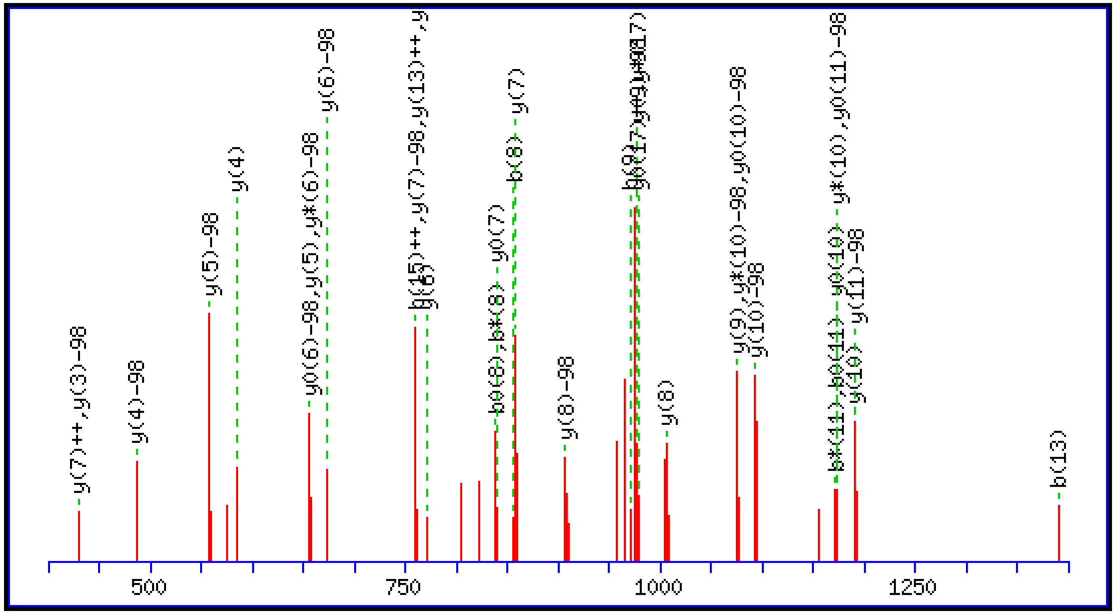

(pS)GQGLEELMNEVVVISK

MS/MS Fragmentation of SGQGLEELMNEVVVISK
Found in AT1G11330.1, | Symbols: | S-locus lectin protein kinase family protein | chr1:3810372-3813416 FORWARD

Match to Query 581: 1912.085448 from(957.050000,2+) intensity(3422920.0000)
Title: Cmpd 245, +MSn(957.4), 29.2 min

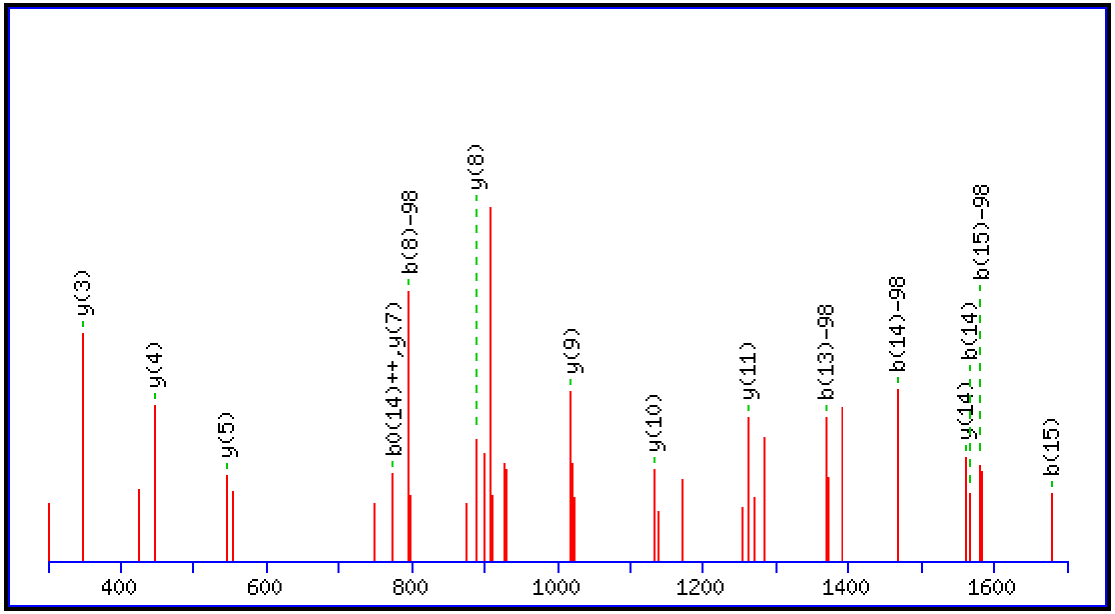

LLDDL(pT)DVEIE

MS/MS Fragmentation of **LLDDLTDVEIE**
Found in **AT1G53440.1**, | Symbols: | leucine-rich repeat family protein / protein kinase family protein | chr1:19945959-19951562 FORWARD

Match to Query 129: 1353.905448 from(677.960000,2+) intensity(161627.0000)
Title: Cmpd 109, +MSn(678.4), 22.4 min

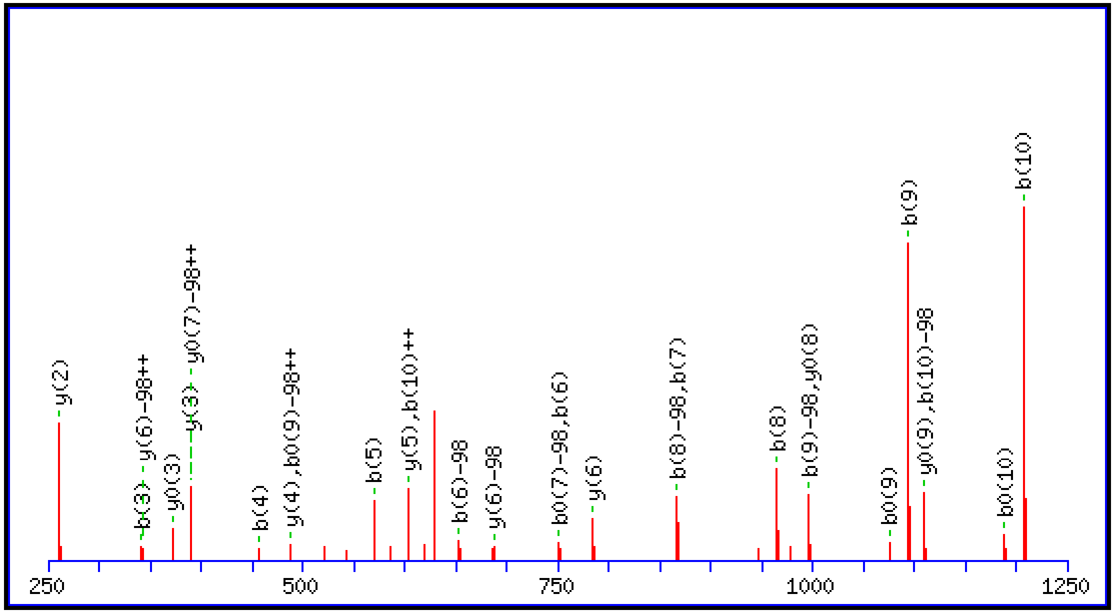

AD(pT)EEDESLDEFSLK

MS/MS Fragmentation of **ADTEEDESLDEFSLK**
Found in **AT1G55610.1**, | Symbols: BRL1 | BRL1 (BRI 1 LIKE); kinase |
chr1:20779874-20783374 REVERSE

Match to Query 459: 1807.145448 from(904.580000,2+) intensity(1082338.0000)
Title: Cmpd 58, +MSn(904.9), 21.5 min

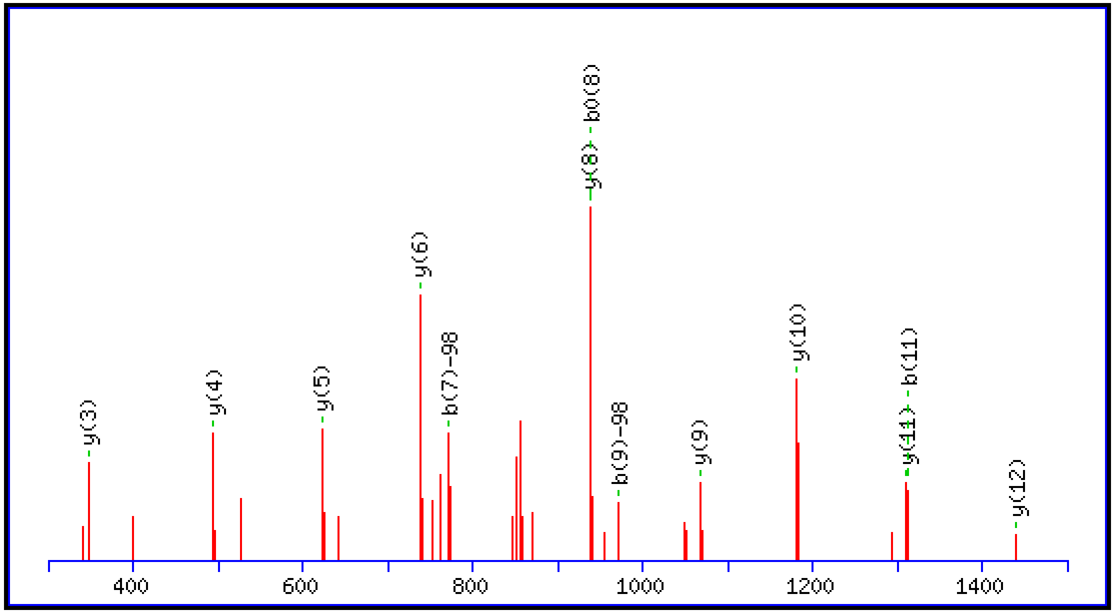

AD(pT)EEDE(pS)LDEFSLK

MS/MS Fragmentation of **ADTEEDESLDEFSLK**
Found in **AT1G55610.1**, | Symbols: BRL1 | BRL1 (BRI 1 LIKE); kinase |
chr1:20779874-20783374 REVERSE

Match to Query 562: 1887.005448 from(944.510000,2+) intensity(234221.0000)
Title: Cmpd 39, +MSn(944.9), 19.2 min

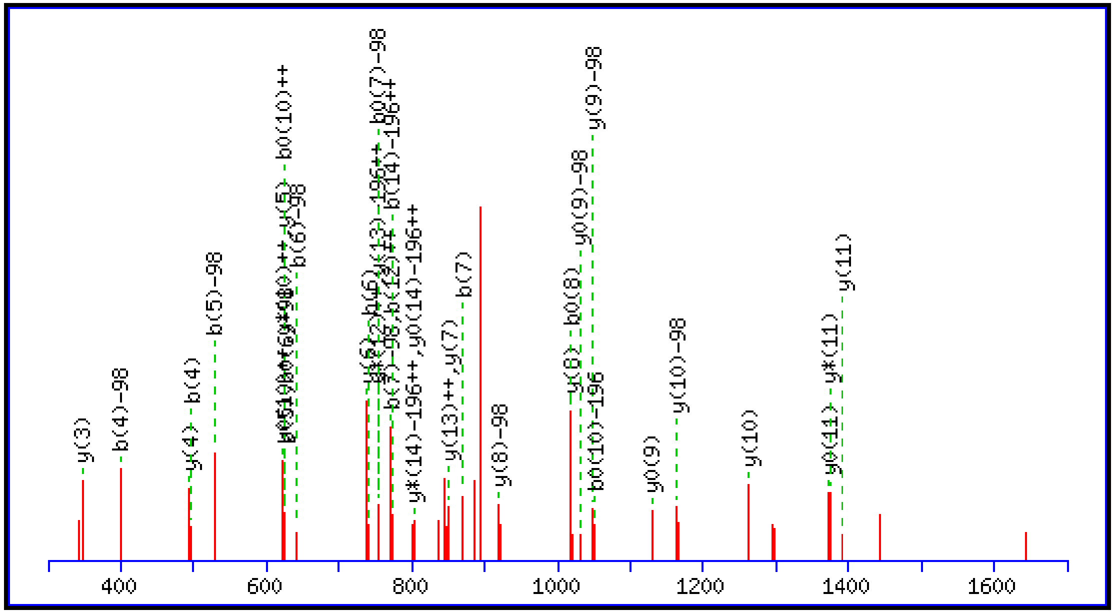

FDLSSAD(pS)PPSK

MS/MS Fragmentation of **FDLSSADSPPSK**
Found in **AT1G71860.1**, | Symbols: PTP1, ATPTP1 | PTP1 (PROTEIN TYROSINE PHOSPHATASE 1); protein tyrosine phosphatase | chr1:27026866-27028675 FORWARD

Match to Query 95: 1329.725448 from(665.870000,2+) intensity(129195.0000)
Title: Cmpd 55, +MSn(666.5), 15.4 min

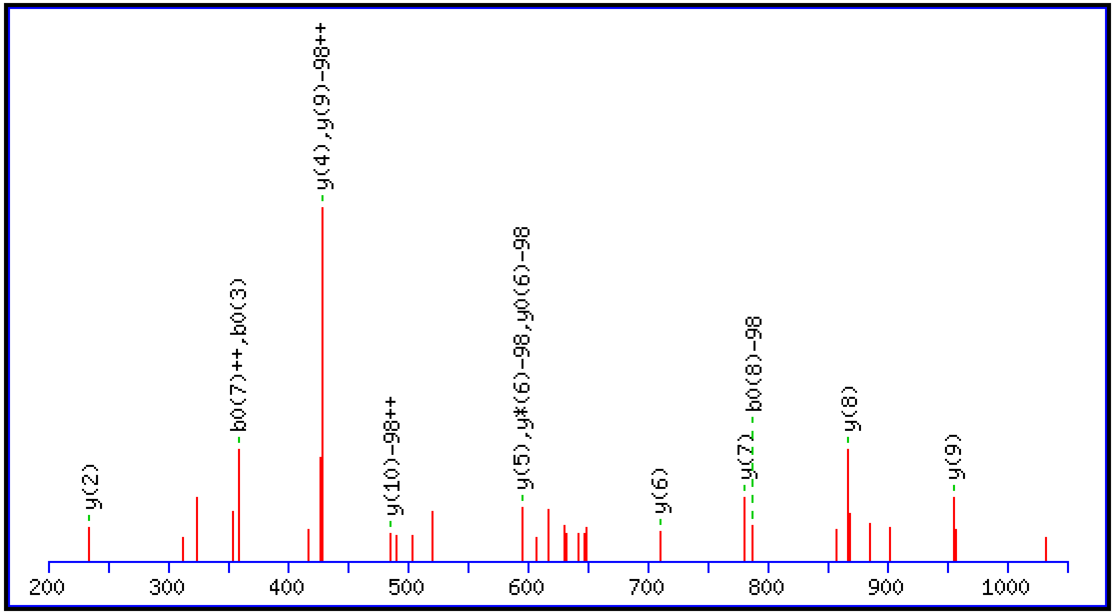

ELVQVDTEND(pS)LDEFLLK

MS/MS Fragmentation of **ELVQVDTENDSLDEFLLK**
Found in **AT3G13380.1**, | Symbols: BRL3 | BRL3 (BRI1-LIKE 3); ATP binding / protein binding / protein kinase/ protein serine/threonine kinase | chr3:4347240-4350734 FORWARD

Match to Query 708: 2186.265448 from(1094.140000,2+) intensity(186817.0000)
Title: Cmpd 136, +MSn(1094.6), 25.3 min

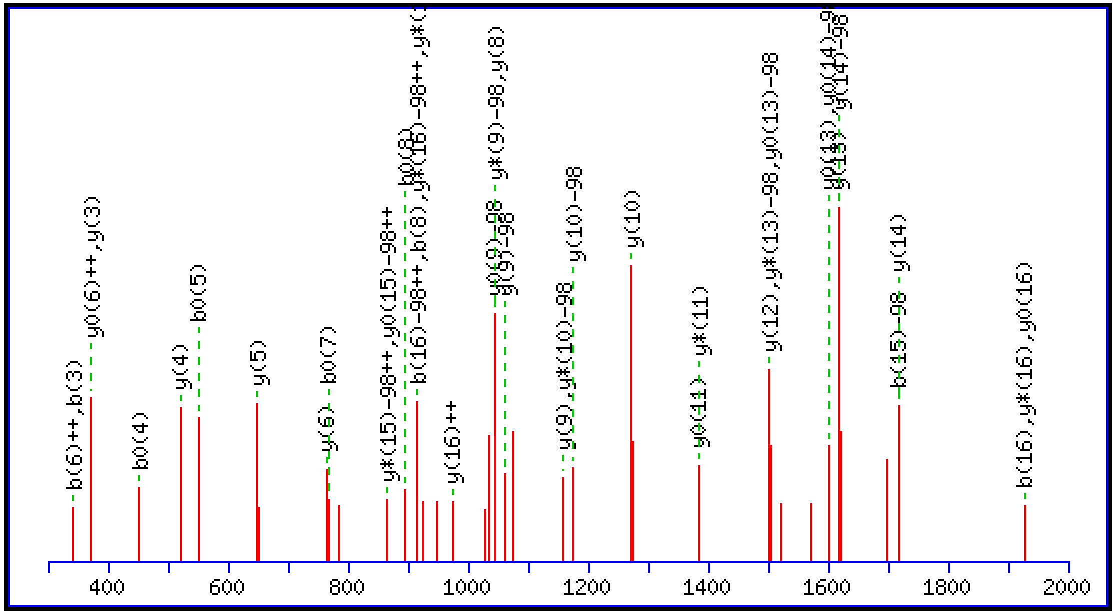

VSEGKPNEASTSMPTSNVNQGD(pS)PVADGGK

MS/MS Fragmentation of **VSEGKPNEASTSMPTSNVNQGDSPVADGGK**
Found in **AT3G13530.1**, | Symbols: MAPKKK7, MAP3KE1 | MAPKKK7; ATP binding / binding / kinase/ protein kinase/ protein serine/threonine kinase/ protein tyrosine kinase | chr3:4411934-4419320 REVERSE

Match to Query 651: 3040.898172 from(1014.640000,3+) intensity(194684.0000)
Title: Cmpd 26, +MSn(1015.0), 12.8 min

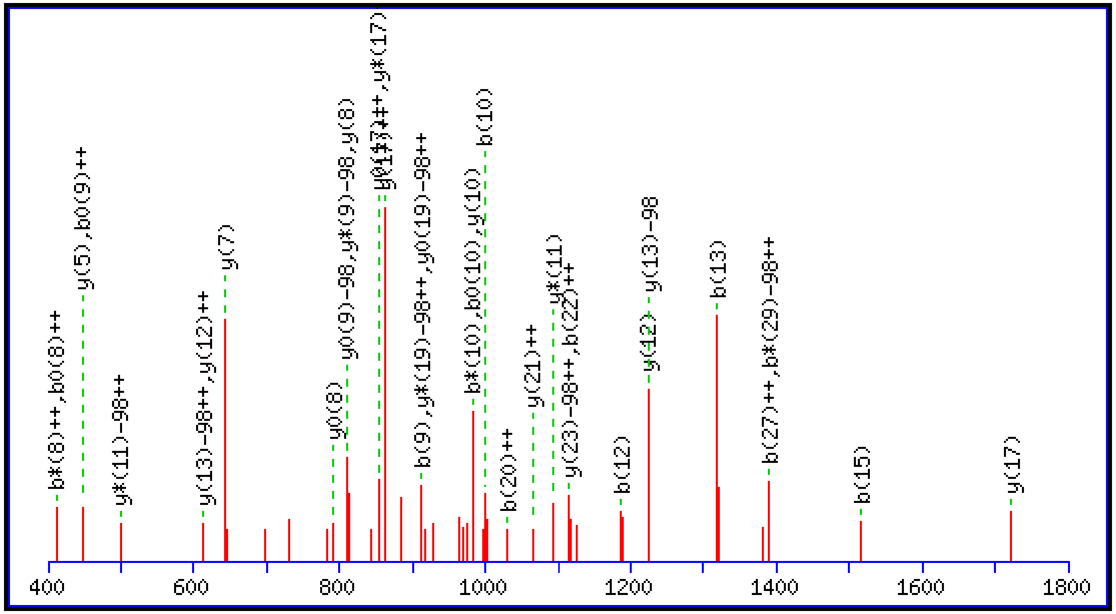

K(s)(s)IE(pS)EDDLEEGDEEDEIGEK

MS/MS Fragmentation of **KSSIESEDDLEEGDEEDEIGEK**

Found in **AT3G24660.1**, | Symbols: TMKL1 | TMKL1 (transmembrane kinase-like 1); ATP binding / kinase/ protein serine/threonine kinase | chr3:9003641-9005751 FORWARD

Match to Query 415: 2641.628172 from(881.550000,3+) intensity(97636.0000)

Title: Cmpd 12, +MSn(882.2), 15.1 min

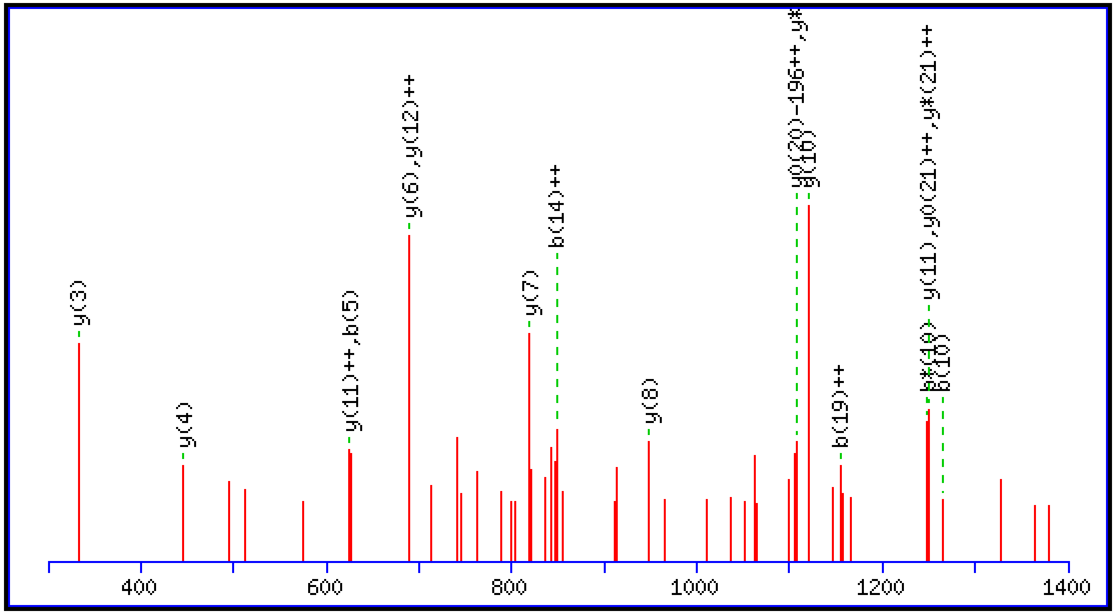

IMNPNDPE(pY)NSDSQSQAPPHPPSSR

MS/MS Fragmentation of **IMNPNDPEYNSDSQSQAPPHPPSSR**
Found in **AT3G25070.1**, | Symbols: RIN4 | RIN4 (RPM1 INTERACTING PROTEIN 4); protein binding | chr3:9132458-9133747 FORWARD

Match to Query 569: 2844.278172 from(949.100000,3+) intensity(132799.0000)
Title: Cmpd 39, +MSn(949.8), 14.4 min

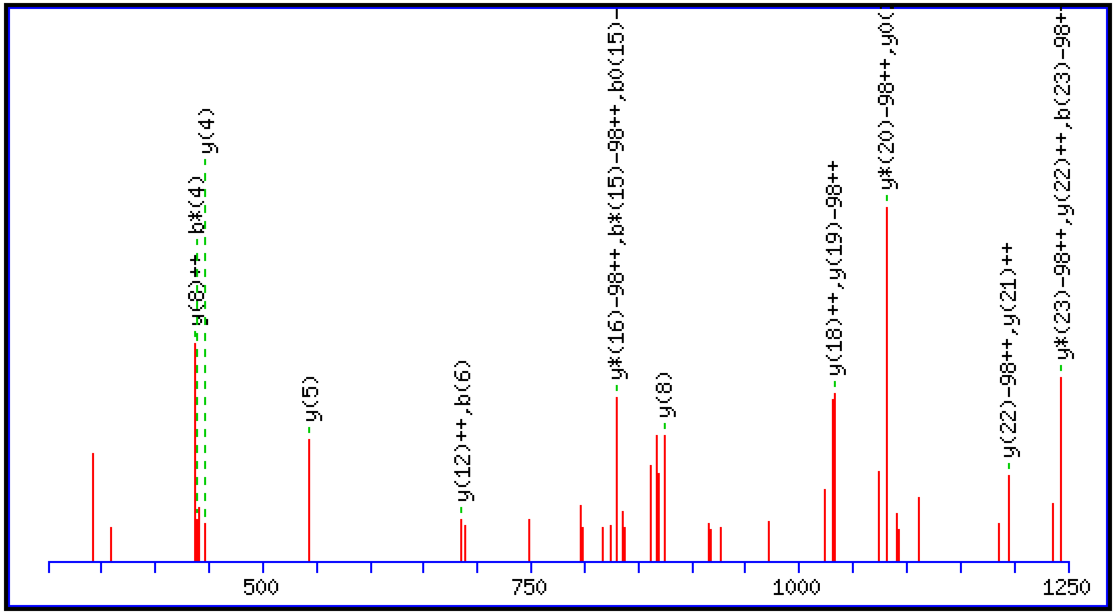

(pS)GLTEVGVSGLAQR

MS/MS Fragmentation of **SGLTEVGVSGLAQR**
Found in **AT3G28450.1**, | Symbols: | leucine-rich repeat transmembrane protein kinase, putative | chr3:10667359-10669176 FORWARD

Match to Query 182: 1453.045448 from(727.530000,2+) intensity(76670.0000)
Title: Cmpd 93, +MSn(727.9), 18.7 min

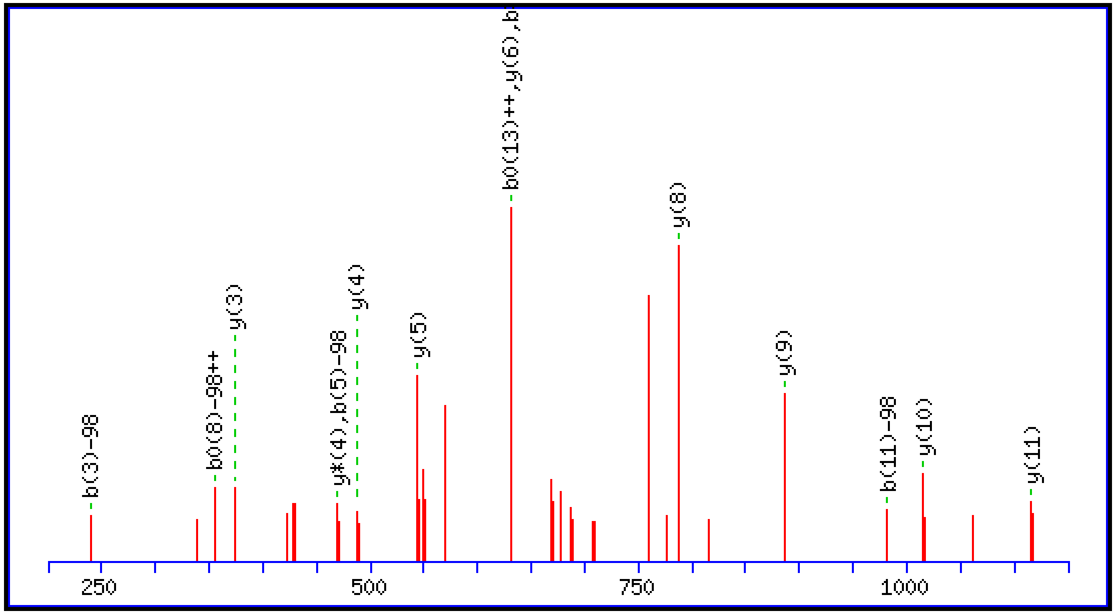

EEW(pT)NEVFDLELMR

MS/MS Fragmentation of **EEWTNEVFDLELMR**

Found in **AT3G51740.1**, | Symbols: IMK2 | IMK2 (INFLORESCENCE MERISTEM RECEPTOR-LIKE KINASE 2); ATP binding / kinase/ protein kinase/ protein serine/threonine kinase | chr3:19189248-19191842 FORWARD

Match to Query 568: 1890.265448 from(946.140000,2+) intensity(219075.0000)

Title: Cmpd 116, +MSn(946.4), 27.2 min

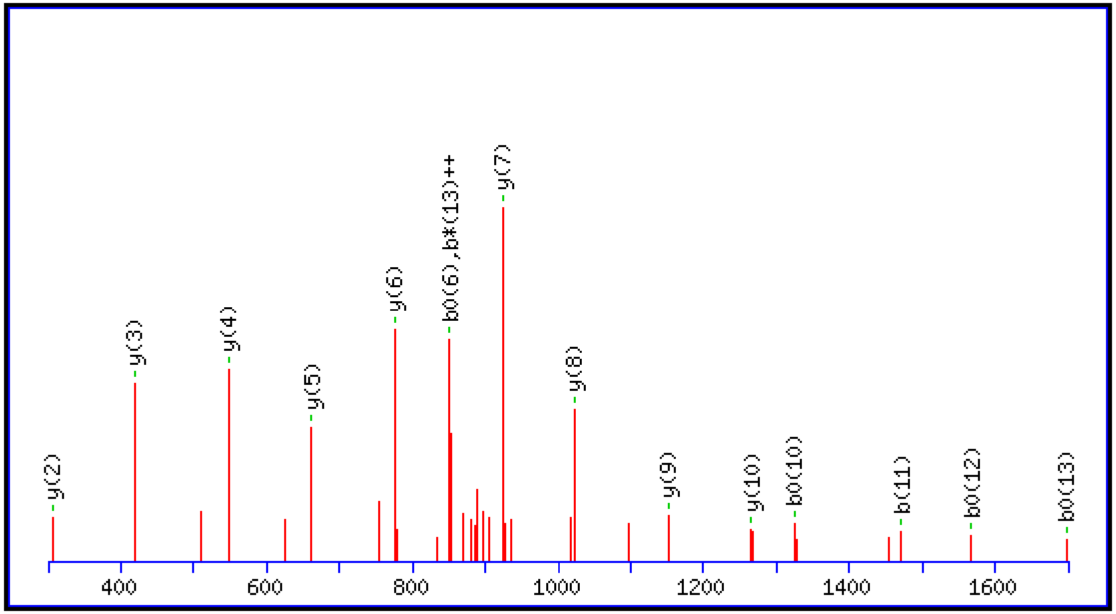

(pT)ESE(pS)DI(pS)TTDSLVYDFK

MS/MS Fragmentation of **TESESDISTTDSLVYDFK**

Found in **AT4G23190.1**, | Symbols: CRK11, AT-RLK3 | CRK11 (CYSTEINE-RICH RLK11); kinase/ protein kinase | chr4:12141197-12143710 REVERSE

Match to Query 732: 2276.005448 from(1139.010000,2+) intensity(114107.0000)

Title: Cmpd 62, +MSn(1139.6), 20.6 min

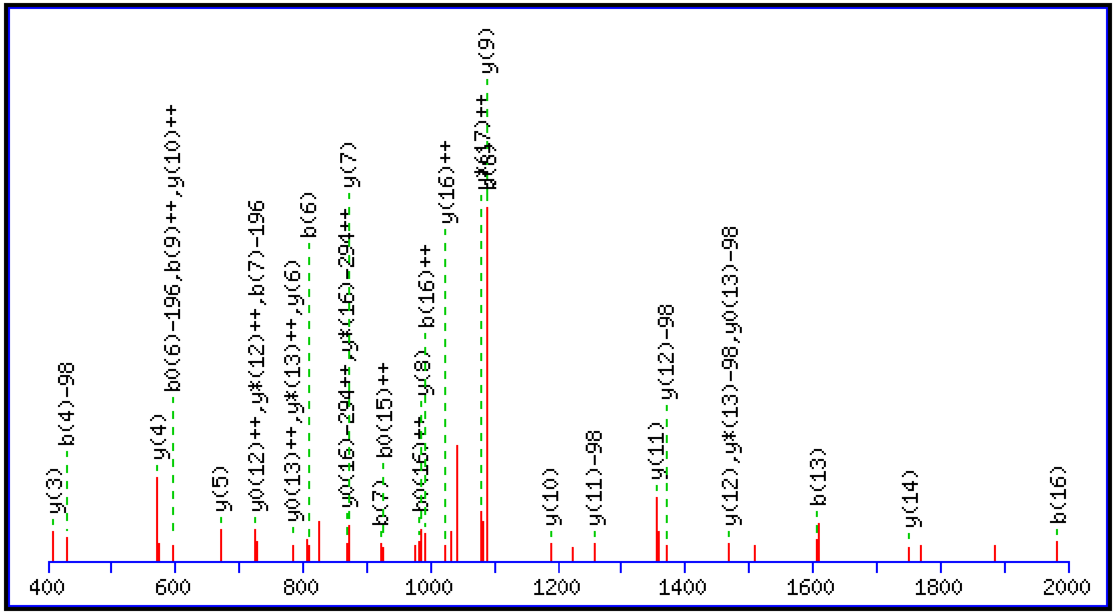

VEDDLDIGDDLMNL(pS)R

MS/MS Fragmentation of **VEDDLDIGDDLMNLSR**

Found in **AT4G24630.1**, | Symbols: | receptor/ zinc ion binding | chr4:12714919-12717111 FORWARD

Match to Query 571: 1899.165448 from(950.590000,2+) intensity(65862.0000)

Title: Cmpd 84, +MSn(950.9), 23.5 min

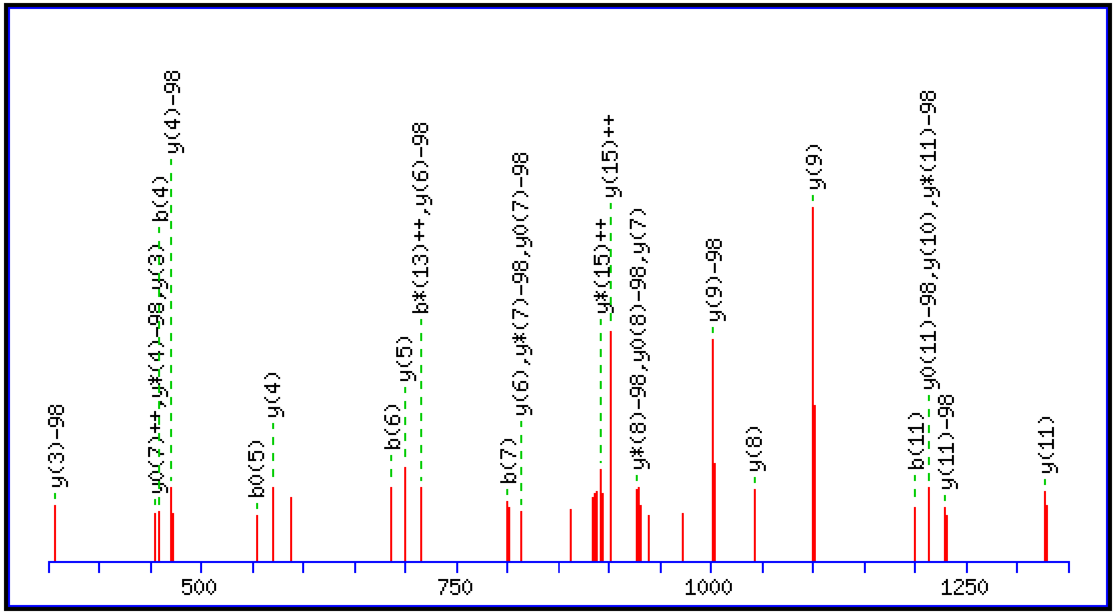

VGPDVPSSADPTSQP(pS)PA

MS/MS Fragmentation of **VGPDVPSSADPTSQPSPA**

Found in **AT4G36180.1**, | Symbols: | leucine-rich repeat family protein |

chr4:17120209-17123698 REVERSE

Match to Query 440: 1787.905448 from(894.960000,2+) intensity(229060.0000)

Title: Cmpd 37, +MSn(895.4), 14.1 min

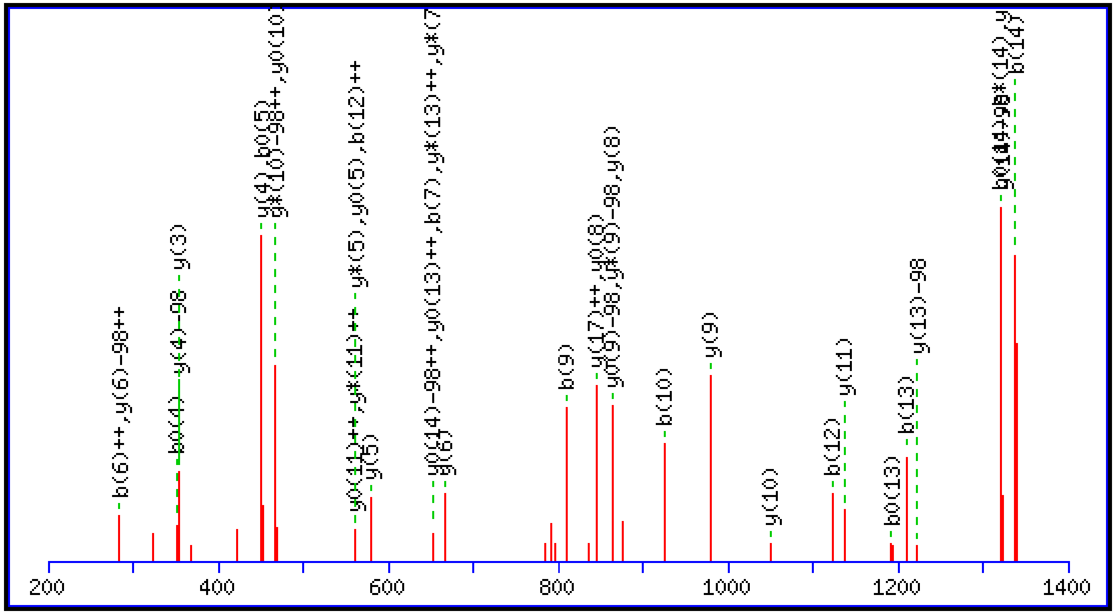

EEW(pT)AEVFDVELLK

MS/MS Fragmentation of **EEWTAEVFDVELLK**
Found in **AT5G05160.1**, | Symbols: | leucine-rich repeat transmembrane protein kinase, putative | chr5:1528000-1530017 FORWARD

Match to Query 439: 1787.165448 from(894.590000,2+) intensity(169418.0000)
Title: Cmpd 117, +MSn(895.0), 27.4 min

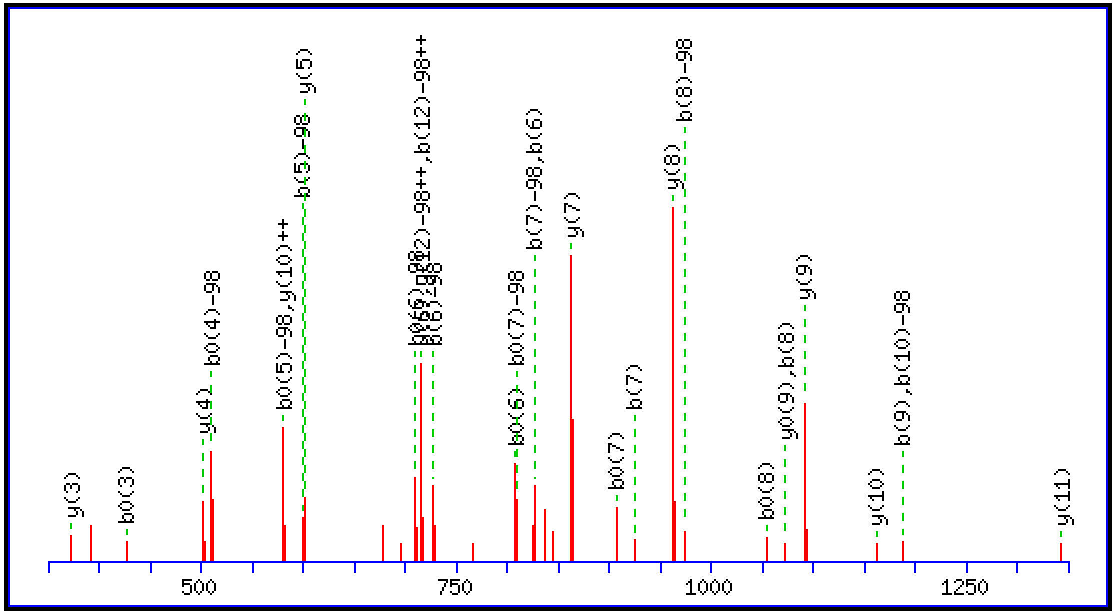

SNPFYSEA(pY)TTNGSGTGFK

MS/MS Fragmentation of **SNPFYSEAYTTNGSGTGFK**
Found in **AT5G19450.1**, | Symbols: CDPK19, CPK8 | CDPK19 (CALCIUM-DEPENDENT PROTEIN KINASE 19); ATP binding / calcium ion binding / calmodulin-dependent protein kinase/ kinase/ protein kinase/ protein serine/threonine kinase | chr5:6558672-6561471 REVERSE

Match to Query 675: 2107.125448 from(1054.570000,2+) intensity(94804.0000)
Title: Cmpd 87, +MSn(1055.3), 18.9 min

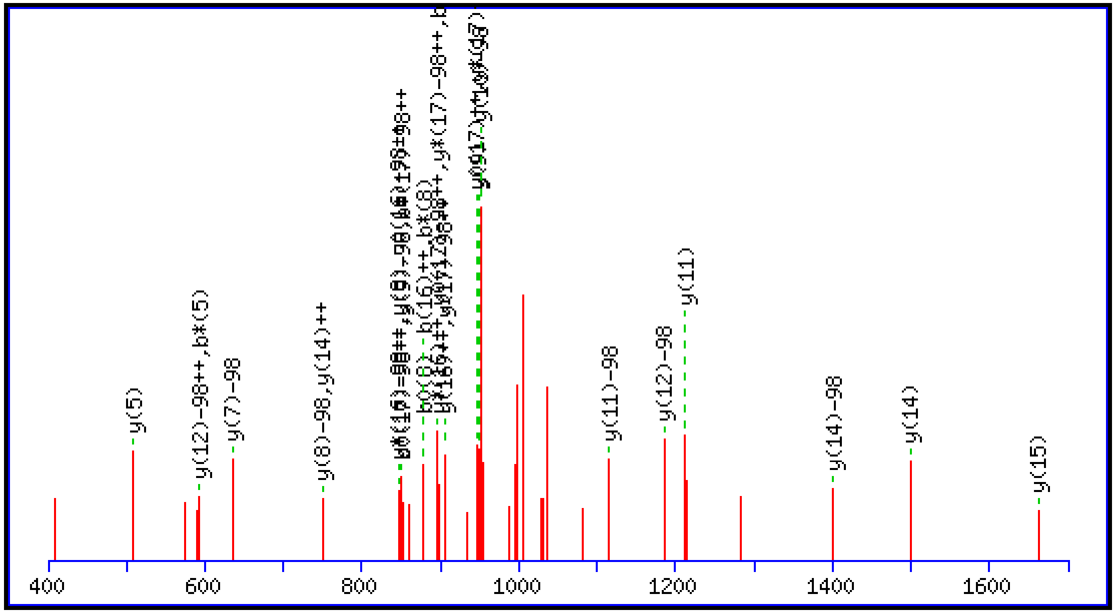

A(pS)AEVLGK

MS/MS Fragmentation of **ASAEVLGK**
Found in **AT5G58300.1**, | Symbols: | leucine-rich repeat transmembrane protein kinase, putative | chr5:23572821-23574871 FORWARD

Match to Query 25: 853.685448 from(427.850000,2+) intensity(947442.0000)
Title: Cmpd 6, +MSn(428.5), 11.7 min

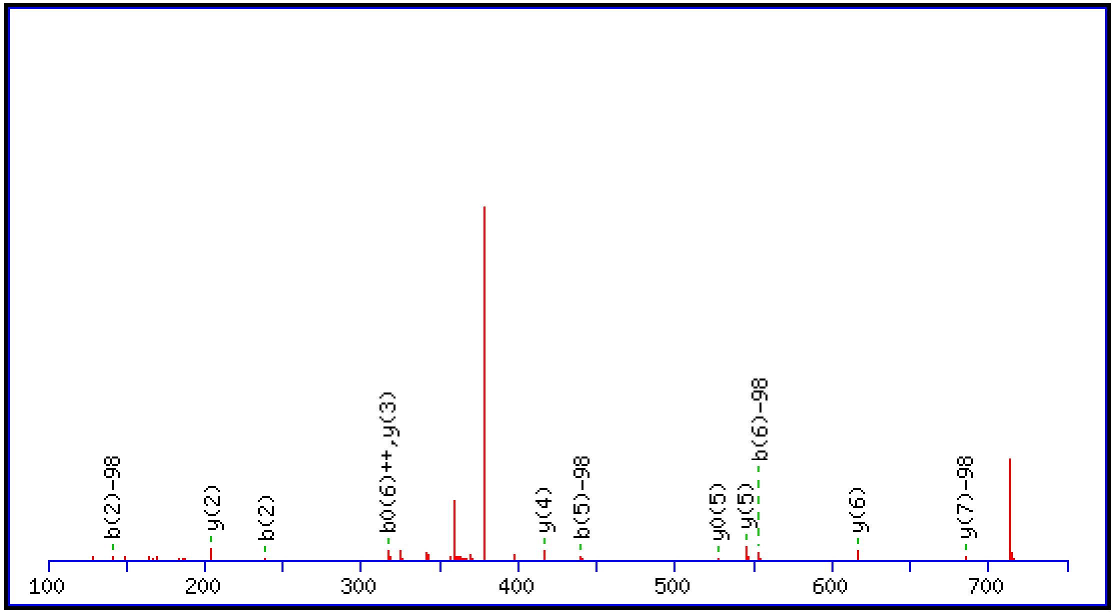

EEW(pT)SEVFDIELMR

MS/MS Fragmentation of **EEWTSEVFDIELMR**
Found in **AT5G58300.1**, | Symbols: | leucine-rich repeat transmembrane protein kinase, putative | chr5:23572821-23574871 FORWARD

Match to Query 536: 1863.205448 from(932.610000,2+) intensity(72929.0000)
Title: Cmpd 97, +MSn(933.1), 27.3 min

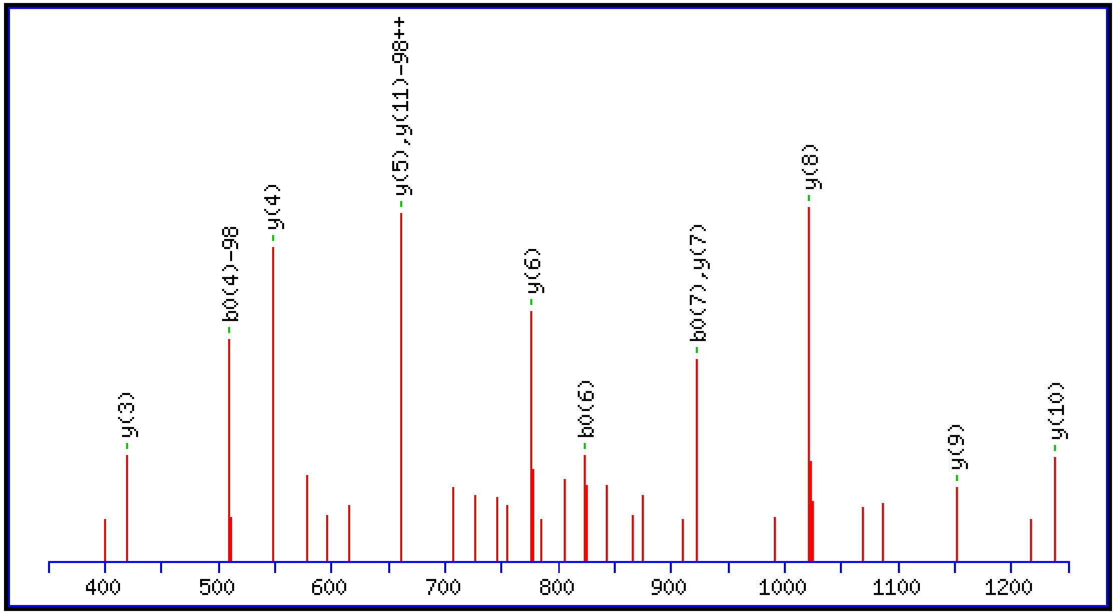

YPLLPNYD(pS)EPDTER

MS/MS Fragmentation of **YPLLPNYDSEPDTER**
Found in **AT5G56890.1**, | Symbols: | protein kinase family protein | chr5:23010801-23015559 REVERSE

Match to Query 566: 1888.165448 from(945.090000,2+) intensity(230498.0000)
Title: Cmpd 74, +MSn(945.6), 19.8 min

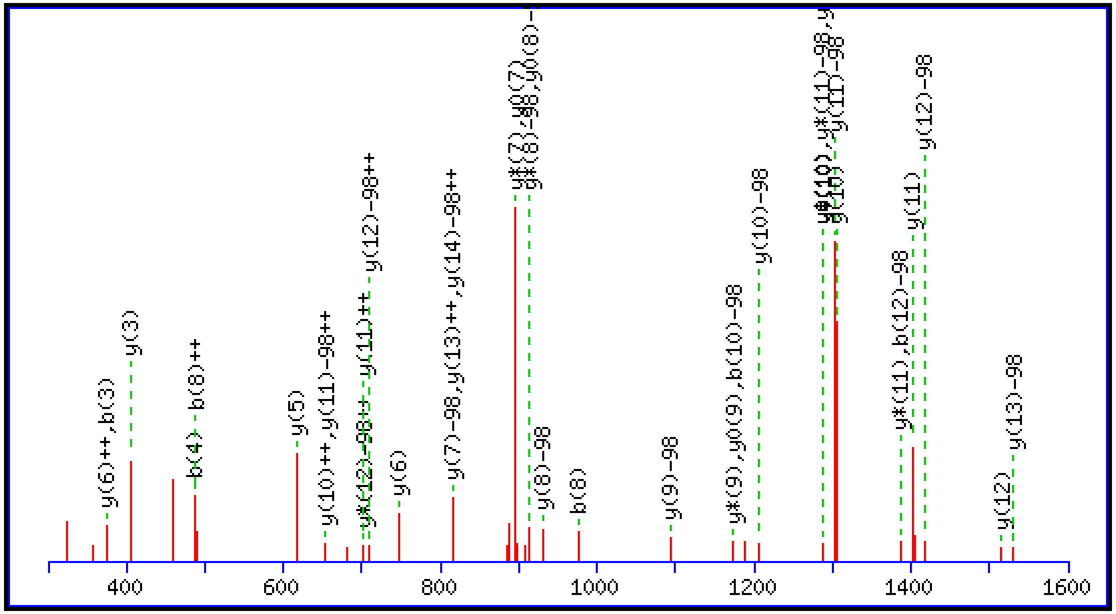

Miscellaneous

FGTETSQELD(pS)ENNVEQHEGSQEVEVIR

MS/MS Fragmentation of **FGTETSQELDSENNVEQHEGSQEVEVIR**

Found in **AT1G10340.2**, | Symbols: | ankyrin repeat family protein | chr1:3390475-3392481 REVERSE

Match to Query 706: 3270.548172 from(1091.190000,3+) intensity(221472.0000)

Title: Cmpd 57, +MSn(1091.3), 17.7 min

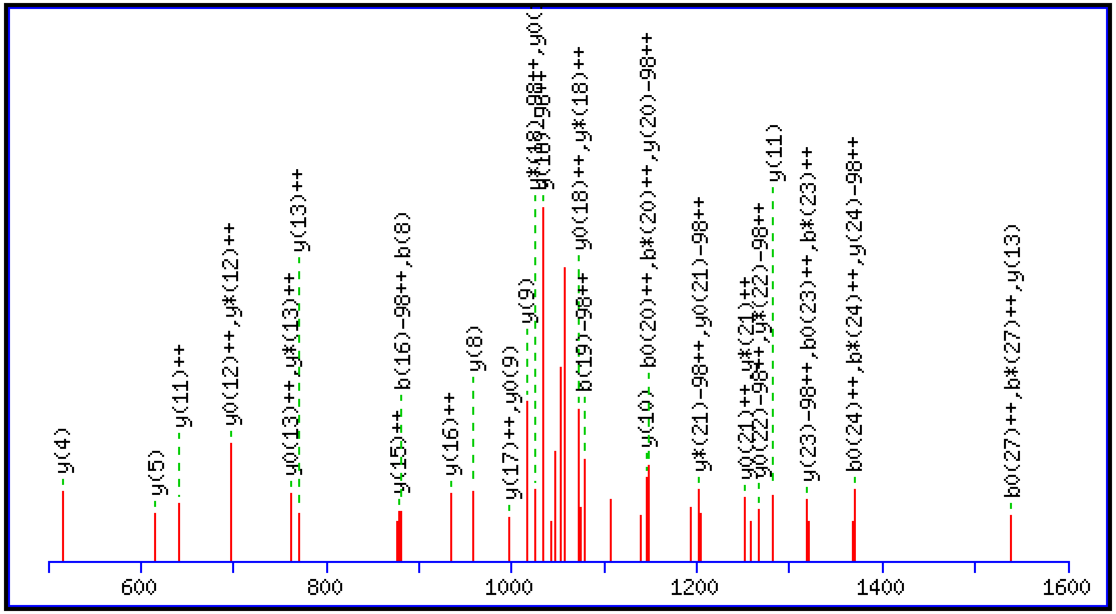

D(pS)FEEWEEAYK

MS/MS Fragmentation of **DSFEEWEEAYK**
Found in **AT1G68720.1**, | Symbols: TADA | TADA (TRNA ARGININE ADENOSINE DEAMINASE); catalytic/ hydrolase/ zinc ion binding | chr1:25804547-25808820 FORWARD

Match to Query 235: 1511.945448 from(756.980000,2+) intensity(77324.0000)
Title: Cmpd 57, +MSn(757.4), 19.3 min

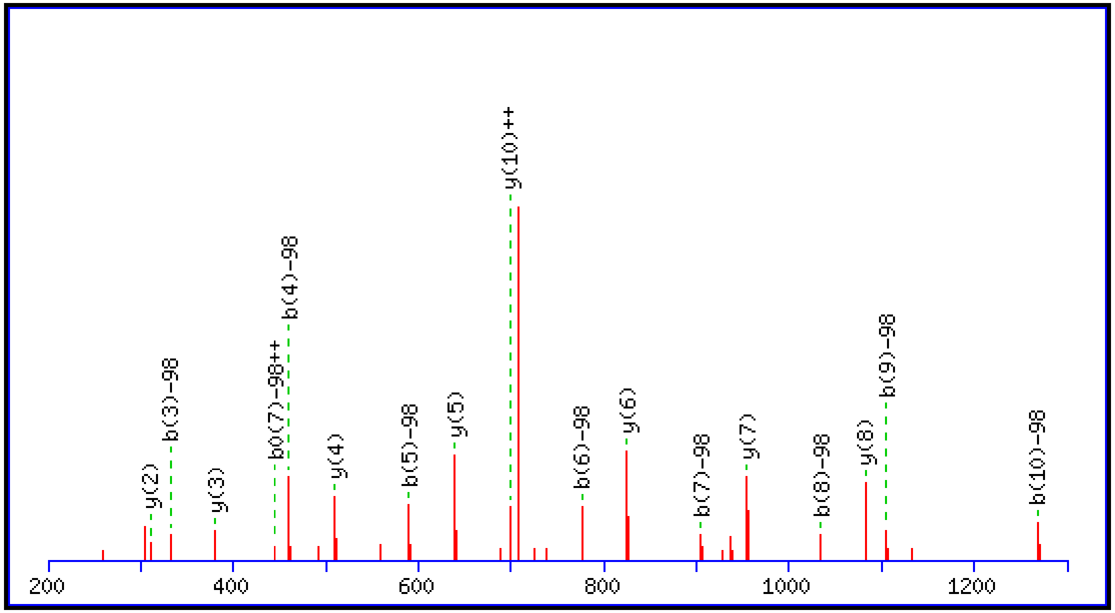

SLPHLIDNDVD(pS)ESVSEAGDIGDR

MS/MS Fragmentation of **SLPHLIDNDVDSESVSEAGDIGDR**

Found in **AT2G41705.1**, | Symbols: | camphor resistance CrcB family protein | chr2:17398083-17399910 FORWARD

Match to Query 399: 2619.608172 from(874.210000,3+) intensity(88136.0000)

Title: Cmpd 96, +MSn(874.9), 21.4 min

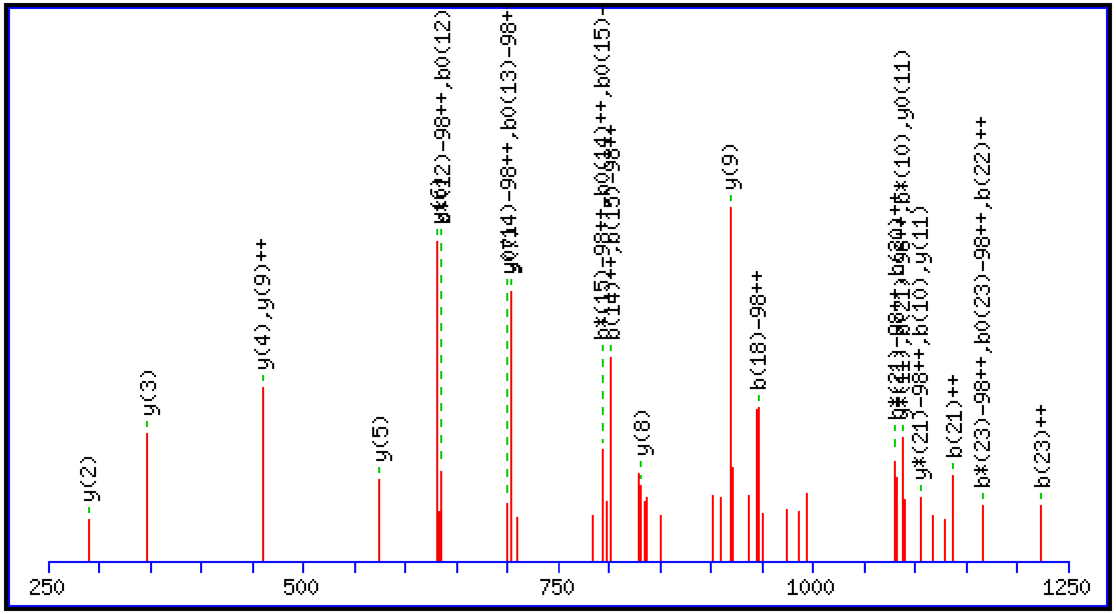

SLPHLIDNDVD(s)E(pS)V(s)EAGDIGDR

MS/MS Fragmentation of **SLPHLIDNDVDSESVSEAGDIGDR**
Found in **AT2G41705.1**, | Symbols: | camphor resistance CrcB family protein |
chr2:17398083-17399910 FORWARD

Match to Query 451: 2699.708172 from(900.910000,3+) intensity(204941.0000)
Title: Cmpd 45, +MSn(901.5), 20.3 min

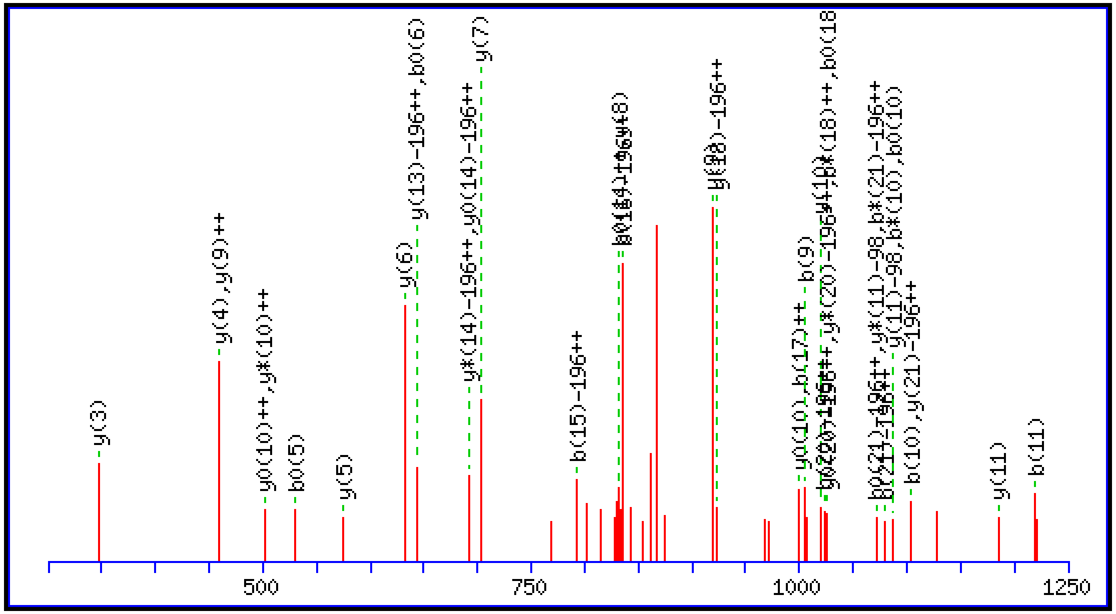

SLPHLIDNDVD(pS)E(pS)V(pS)EAGDIGDR

MS/MS Fragmentation of **SLPHLIDNDVDSESVSEAGDIGDR**

Found in **AT2G41705.1**, | Symbols: | camphor resistance CrcB family protein | chr2:17398083-17399910 FORWARD

Match to Query 531: 2780.588172 from(927.870000,3+) intensity(1777743.0000)

Title: Cmpd 47, +MSn(927.9), 20.7 min

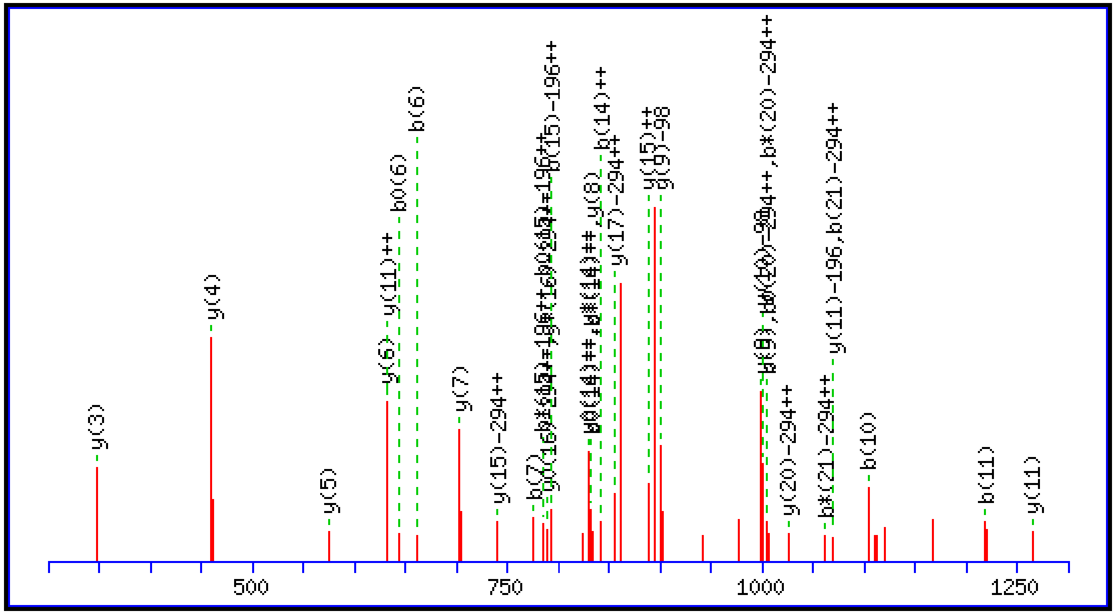

(pS)LPHLIDNDVD(pS)E(pS)V(pS)EAGDIGDR

MS/MS Fragmentation of **SLPHLIDNDVDSESVSEAGDIGDR**

Found in **AT2G41705.1**, | Symbols: | camphor resistance CrcB family protein | chr2:17398083-17399910 FORWARD

Match to Query 575: 2859.428172 from(954.150000,3+) intensity(166745.0000)

Title: Cmpd 45, +MSn(954.7), 19.3 min

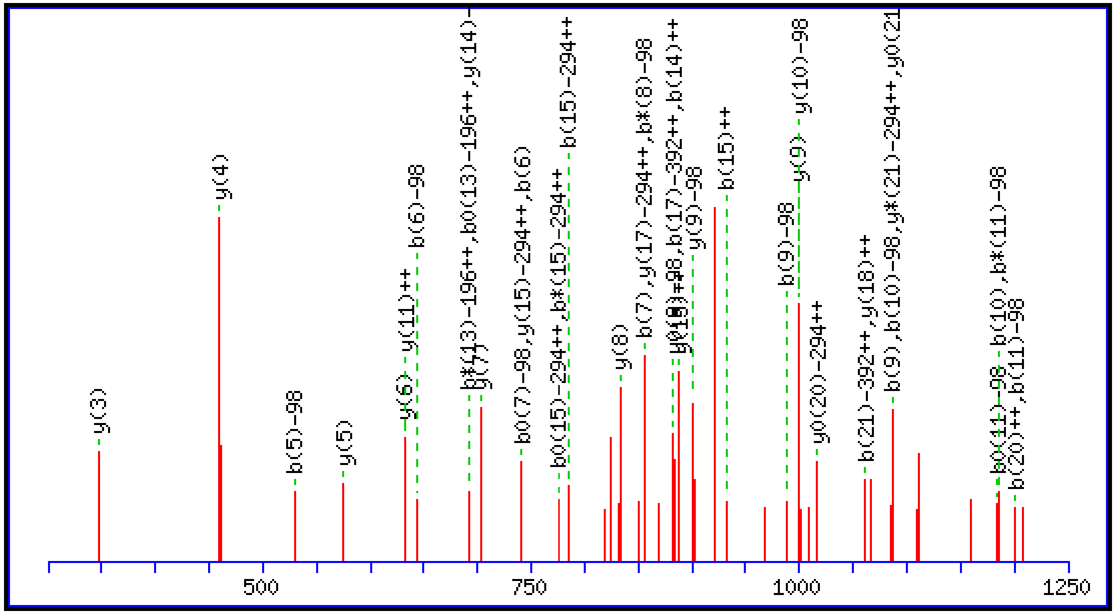

L(pS)ADDFIEQGTHDTSR

MS/MS Fragmentation of **LSADDFIEQGTHDTSR**
Found in **AT2G41705.1**, | Symbols: | camphor resistance CrcB family protein | chr2:17398083-17399910 FORWARD

Match to Query 545: 1871.145448 from(936.580000,2+) intensity(307569.0000)
Title: Cmpd 76, +MSn(936.6), 19.3 min

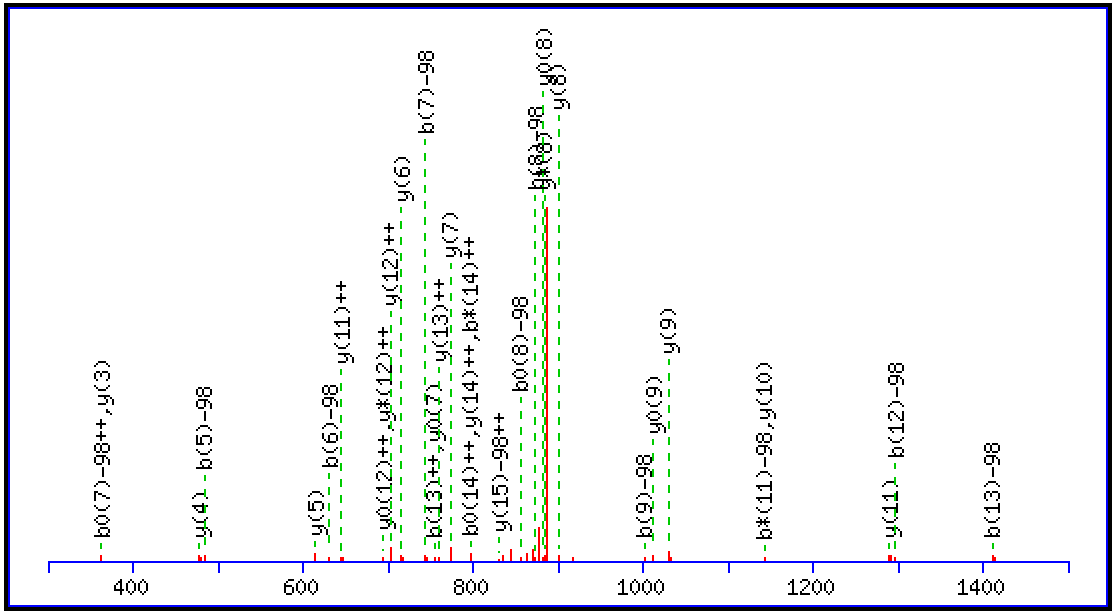

TLPEDITA(pS)PLPTK

MS/MS Fragmentation of **TLPEDITASPLPTK**
Found in **AT2G41705.1**, | Symbols: | camphor resistance CrcB family protein |
chr2:17398083-17399910 FORWARD

Match to Query 267: 1562.105448 from(782.060000,2+) intensity(81381.0000)
Title: Cmpd 75, +MSn(782.3), 19.2 min

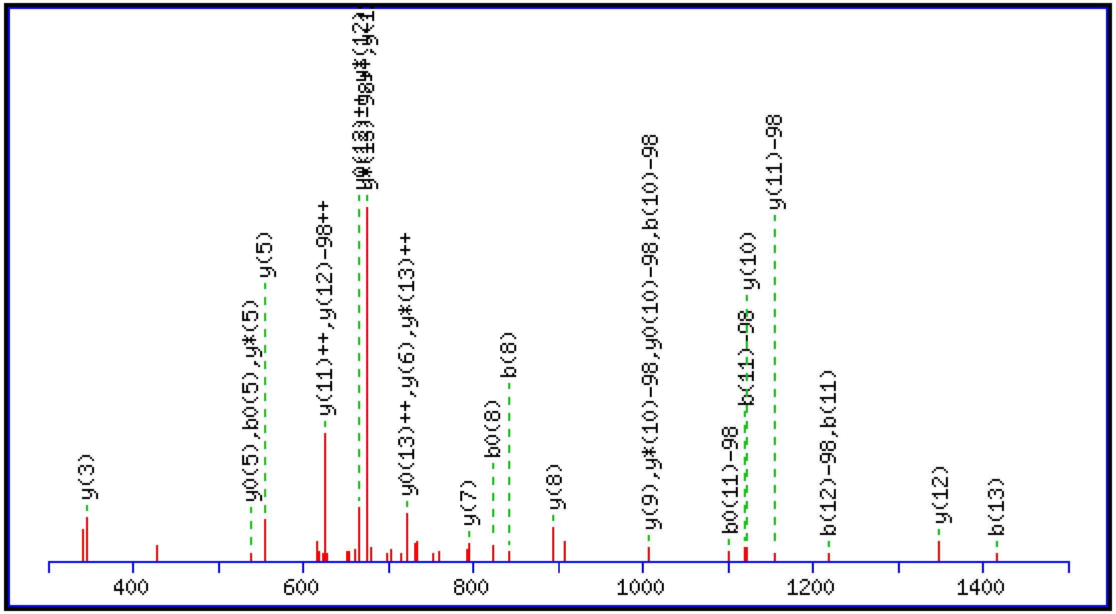

SLL(pS)PEINNSGK

MS/MS Fragmentation of **SLLSPEINNSGK**

Found in **AT2G41705.1**, | Symbols: | camphor resistance CrcB family protein | chr2:17398083-17399910 FORWARD

Match to Query 88: 1338.005448 from(670.010000,2+) intensity(1552408.0000)

Title: Cmpd 104, +MSn(670.3), 18.7 min

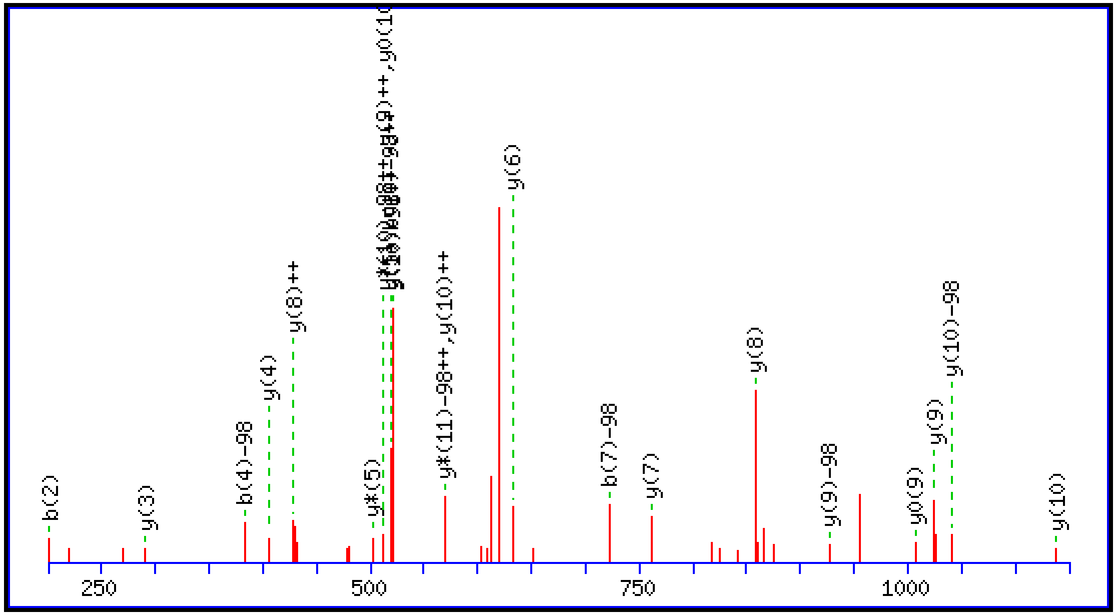

YELQEIYGIGN(pT)VEGDDDSADDANDPGK

MS/MS Fragmentation of **YELQEIYGIGNTVEGDDDSADDANDPGK**

Found in **AT3G09770.1**, | Symbols: | zinc finger (C3HC4-type RING finger) family protein | chr3:2996402-2997835 REVERSE

Match to Query 661: 3079.718172 from(1027.580000,3+) intensity(141776.0000)

Title: Cmpd 73, +MSn(1028.0), 21.6 min

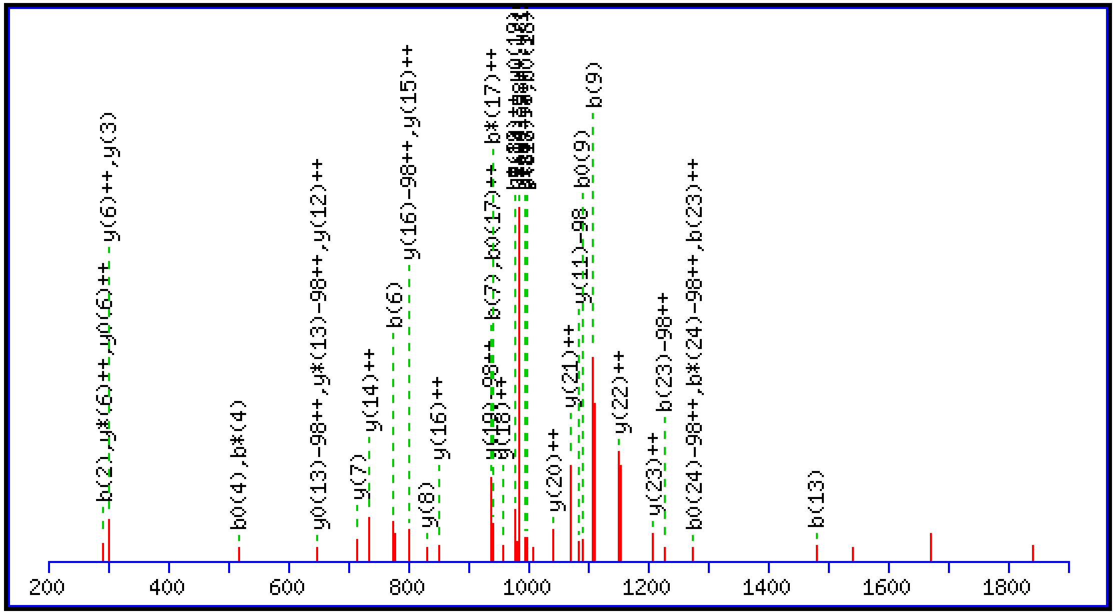

LLEDIGDESEAQAE(pS)EED

MS/MS Fragmentation of **LLEDIGDESEAQAESEED**

Found in **AT3G27530.1**, | Symbols: GC6 | GC6 (golgin candidate 6); binding / protein transporter | chr3:10193778-10199659 REVERSE

Match to Query 664: 2058.025448 from(1030.020000,2+) intensity(131615.0000)

Title: Cmpd 39, +MSn(1030.4), 16.1 min

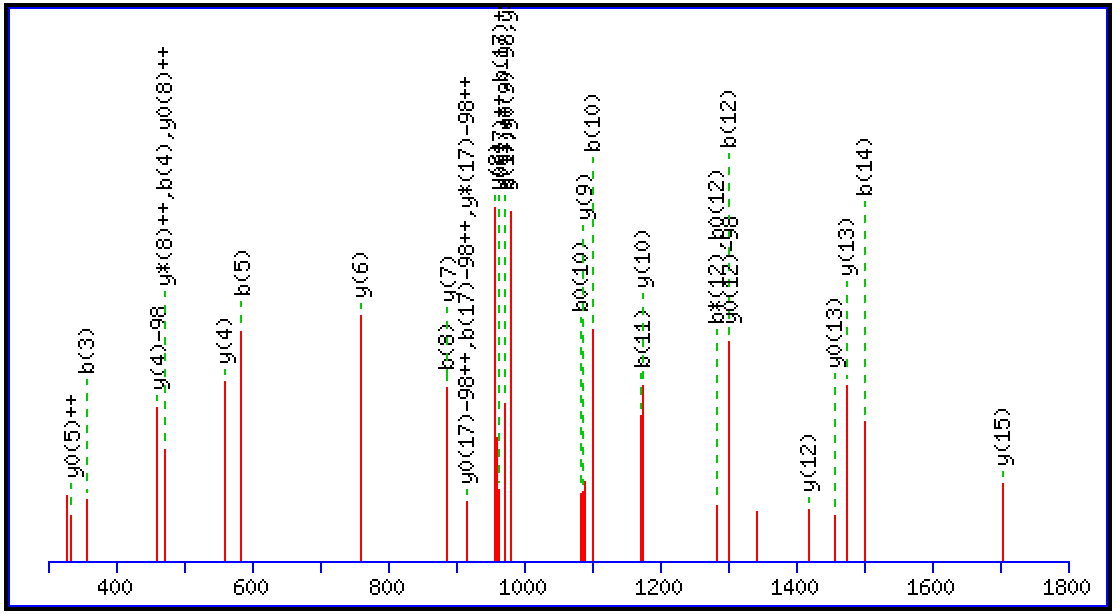

GY(pS)PPVDVQR

MS/MS Fragmentation of **GYSPPVDVQR**

Found in **AT3G28850.1**, | Symbols: | glutaredoxin family protein | chr3:10848669-10849955

FORWARD

Match to Query 63: 1196.705448 from(599.360000,2+) intensity(219272.0000)

Title: Cmpd 48, +MSn(599.6), 15.4 min

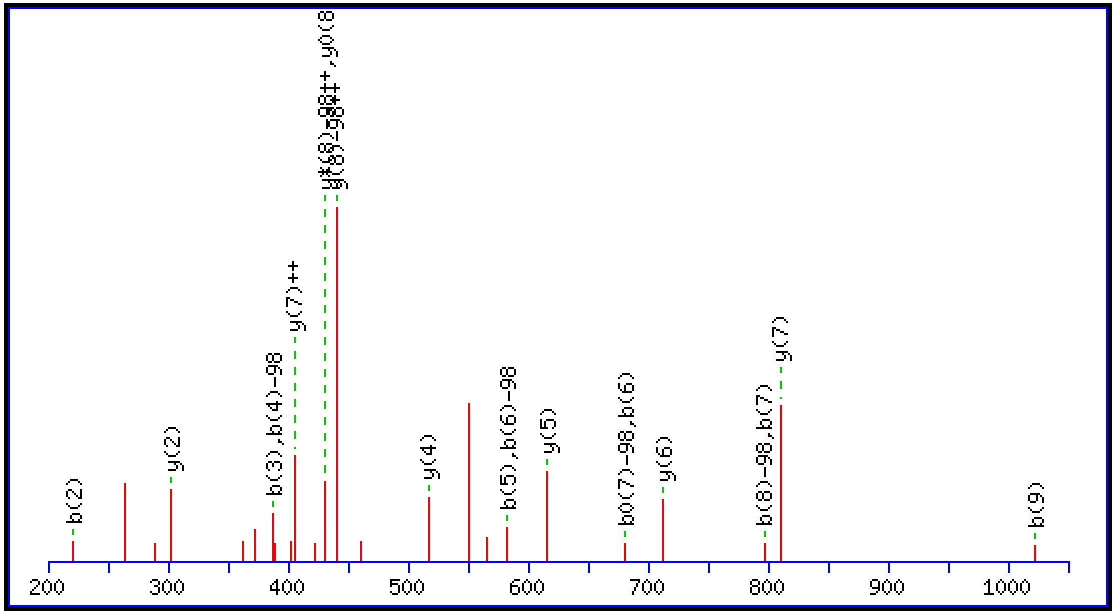

(pS)FSFDVGPNGGK

MS/MS Fragmentation of **SFSFDVGPNGGK**

Found in **AT3G28850.1**, | Symbols: | glutaredoxin family protein | chr3:10848669-10849955

FORWARD

Match to Query 89: 1290.765448 from(646.390000,2+) intensity(148030.0000)

Title: Cmpd 101, +MSn(646.7), 19.3 min

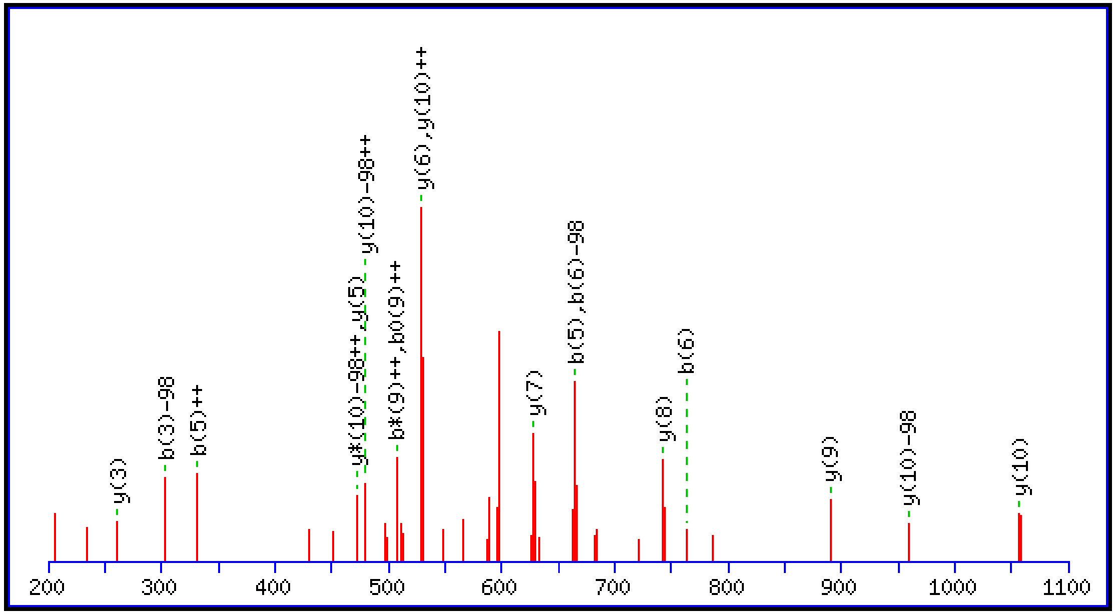

VYYEYEDDDDDDDEGDDDE(pS)VKEER

MS/MS Fragmentation of **VYYEYEDDDDDDDEGDDDESVKEER**

Found in **AT3G28850.1**, | Symbols: | glutaredoxin family protein | chr3:10848669-10849955

FORWARD

Match to Query 672: 3138.338172 from(1047.120000,3+) intensity(141928.0000)

Title: Cmpd 7, +MSn(1047.7), 16.0 min

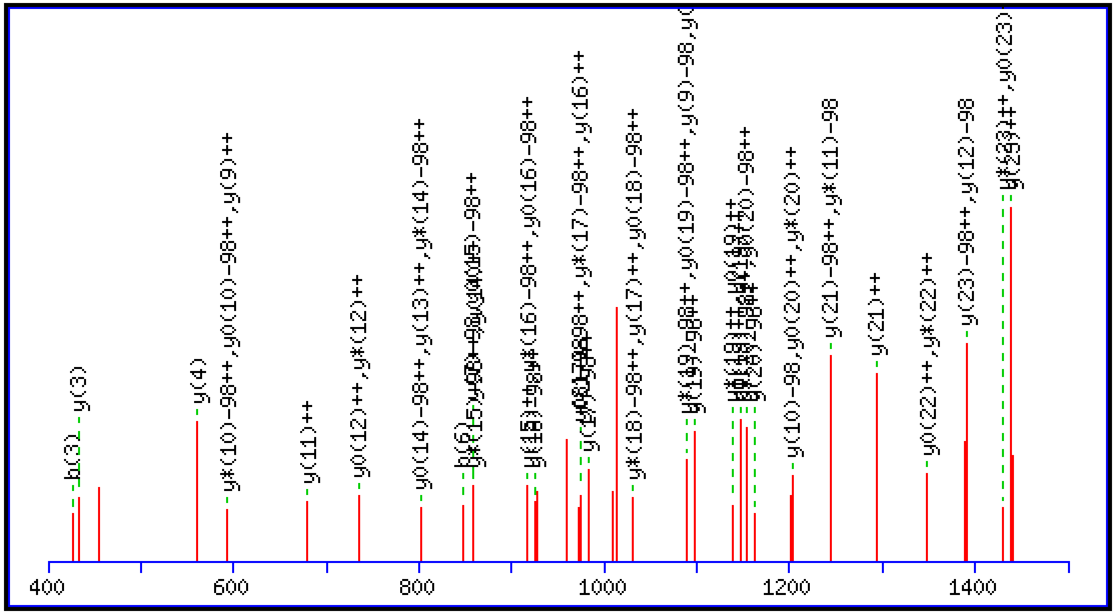

IPEPSVH(pS)EEEVFEDGEEIDGGVR

MS/MS Fragmentation of **IPEPSVHSEEEVFEDGEEIDGGVR**

Found in **AT4G18950.1**, | Symbols: | ankyrin protein kinase, putative | chr4:10375685-10378129 FORWARD

Match to Query 473: 2734.628172 from(912.550000,3+) intensity(200711.0000)

Title: Cmpd 104, +MSn(913.0), 21.7 min

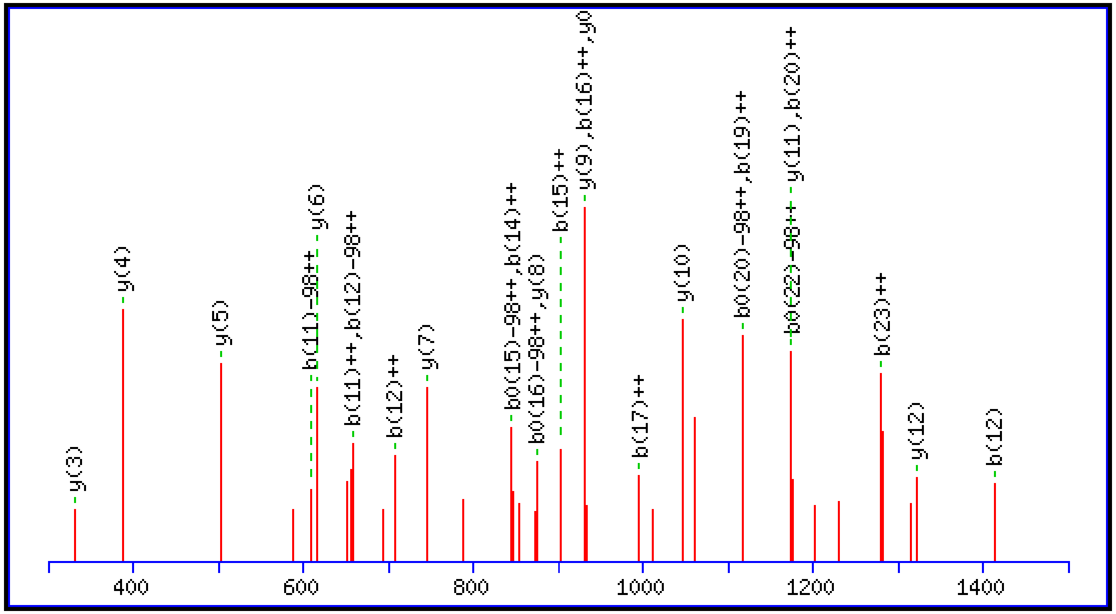

SFVVEE(pS)DDDMDETEEVKPK

MS/MS Fragmentation of **SFVVEESDDDMDETEEVKPK**

Found in **AT4G22670.1**, | Symbols: AtHip1 | AtHip1 (Arabidopsis thaliana Hsp70-interactin protein 1); binding | chr4:11918236-11920671 FORWARD

Match to Query 285: 2407.388172 from(803.470000,3+) intensity(109784.0000)

Title: Cmpd 25, +MSn(803.6), 17.4 min

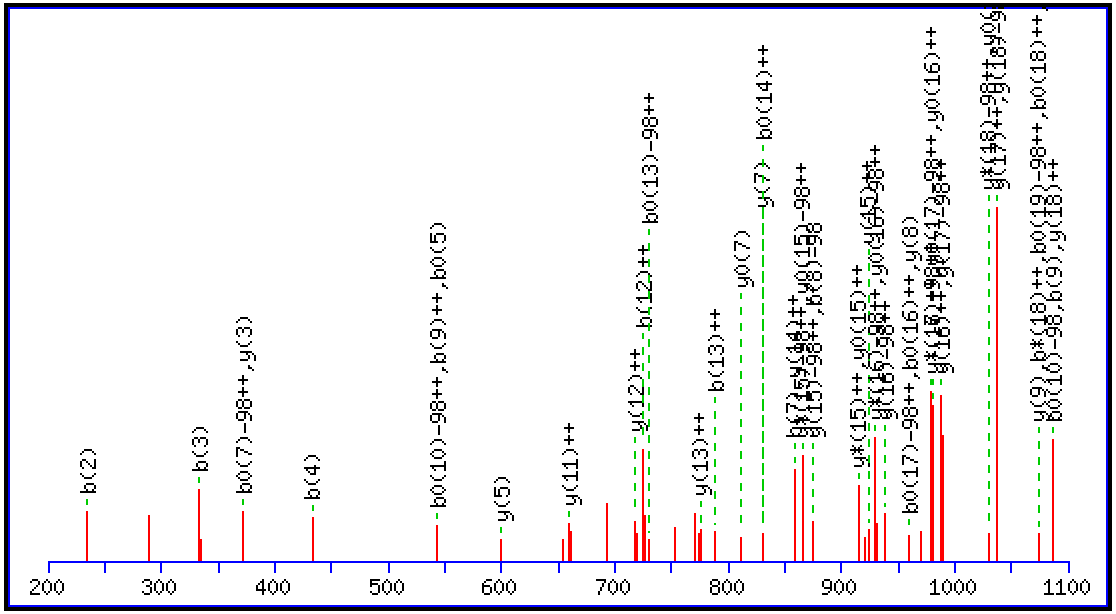

IGIWQYGDIE(pS)DDEDTGPAR

MS/MS Fragmentation of **IGIWQYGDIESDDEDTGPAR**

Found in **AT5G07350.1**, | Symbols: | tudor domain-containing protein / nuclease family prote:
| chr5:2320344-2324892 REVERSE

Match to Query 253: 2316.548172 from(773.190000,3+) intensity(65850.0000)

Title: Cmpd 108, +MSn(773.5), 22.2 min

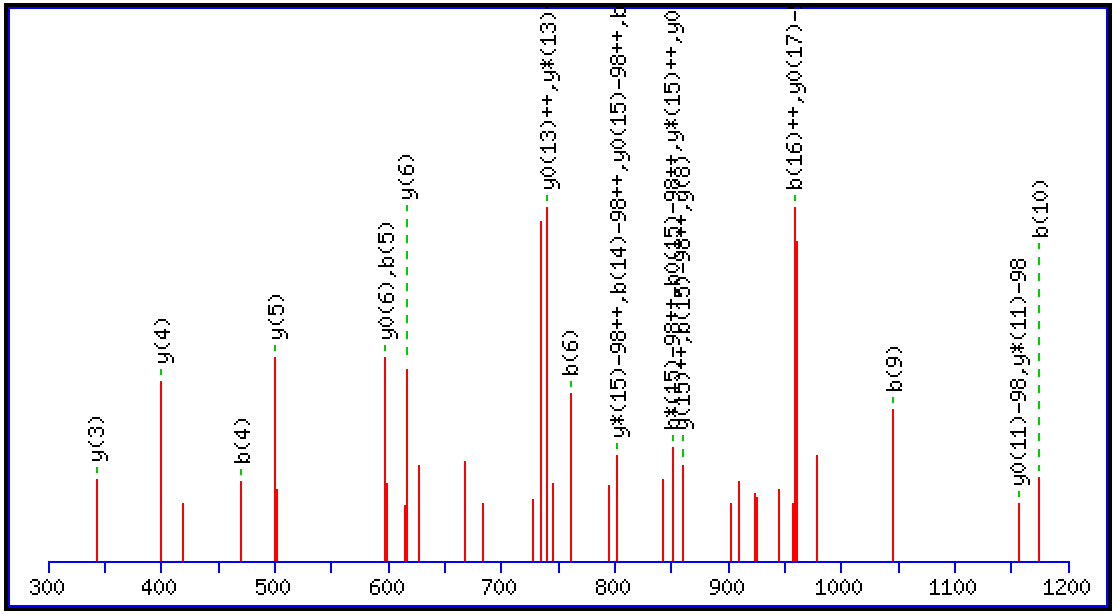

IAGDEENNGPDD(pS)DDELNIK

MS/MS Fragmentation of **IAGDEENNGPDDSDDELNIK**

Found in **AT5G44030.1**, | Symbols: CESA4, IRX5, NWS2 | CESA4 (CELLULOSE SYNTHASE A4); cellulose synthase/ transferase, transferring glycosyl groups | chr5:17714713-17719564 FORWARD

Match to Query 219: 2239.508172 from(747.510000,3+) intensity(162030.0000)

Title: Cmpd 15, +MSn(748.0), 15.3 min

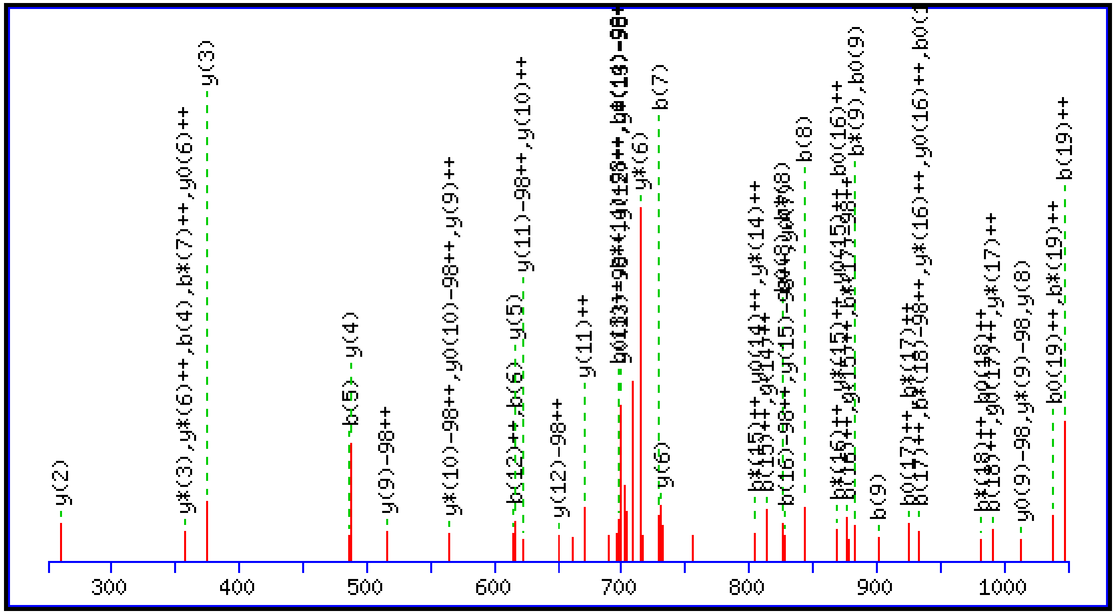

DPWGGPLEINTAD(pS)A(pT)DDDR

MS/MS Fragmentation of **DPWGGPLEINTADSATDDDR**
Found in **AT5G49720.1**, | Symbols: ATGH9A1, DEC, KOR, RSW2, IRX2, KOR1 | ATGH9A1 (ARABIDOPSIS THALIANA GLYCOSYL HYDROLASE 9A1); cellulase/ hydrolase, hydrolyzing O-glycosyl compounds | chr5:20197765-20200168 REVERSE

Match to Query 733: 2304.005448 from(1153.010000,2+) intensity(119670.0000)
Title: Cmpd 60, +MSn(1153.5), 20.4 min

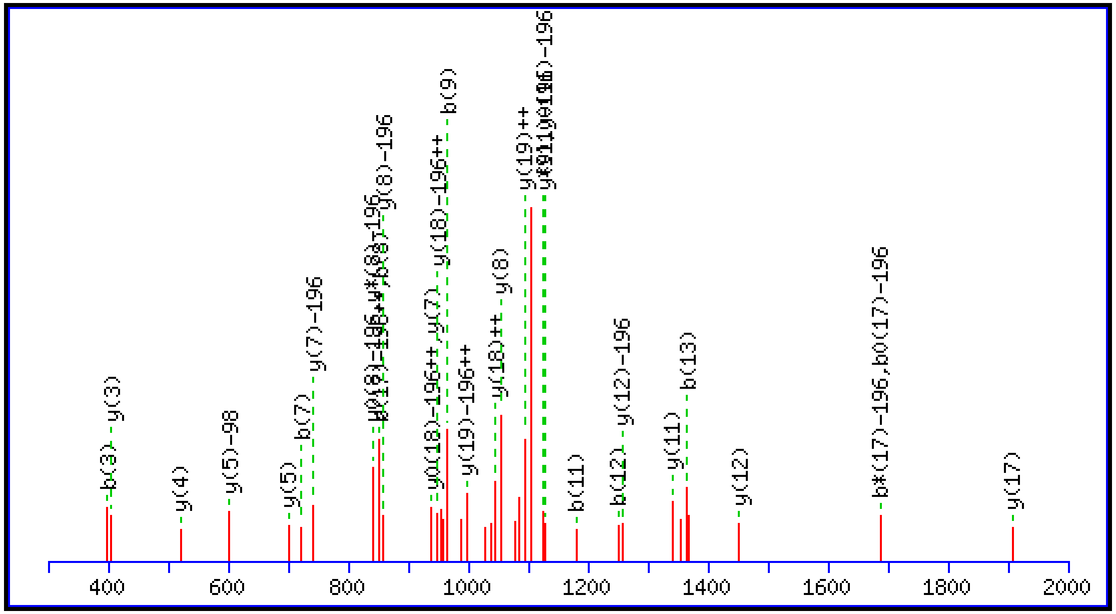

EIAEGVTQIVQMLE(pT)EEE

MS/MS Fragmentation of **EIAEGVTQIVQMLETEEE**
Found in **AT5G62390.1**, | Symbols: ATBAG7 | ATBAG7 (ARABIDOPSIS THALIANA BCL-2-ASSOCIATED ATHANOGENE 7); calmodulin binding | chr5:25052377-2505417 REVERSE

Match to Query 678: 2127.045448 from(1064.530000,2+) intensity(106352.0000)
Title: Cmpd 132, +MSn(1065.1), 27.5 min

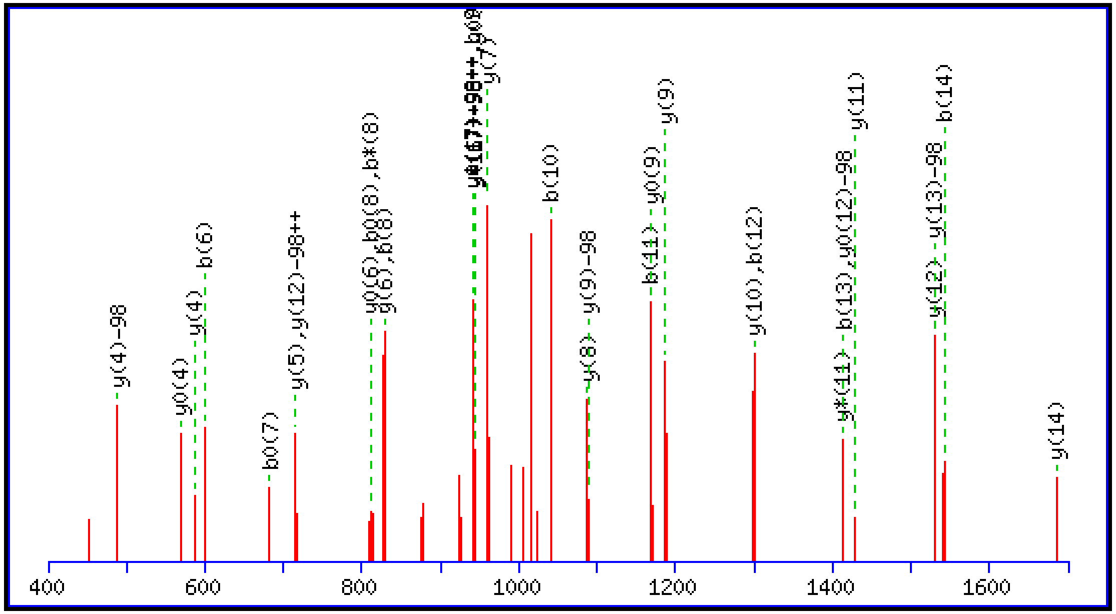

Unknown

TDSEVTSLAAS(pS)PAR

MS/MS Fragmentation of **TDSEVTSLAASSPAR**

Found in **AT1G45688.1**, | Symbols: | unknown protein | chr1:17191502-17192870 FORWARD

Match to Query 272: 1570.825448 from(786.420000,2+) intensity(135892.0000)

Title: Cmpd 35, +MSn(786.7), 14.0 min

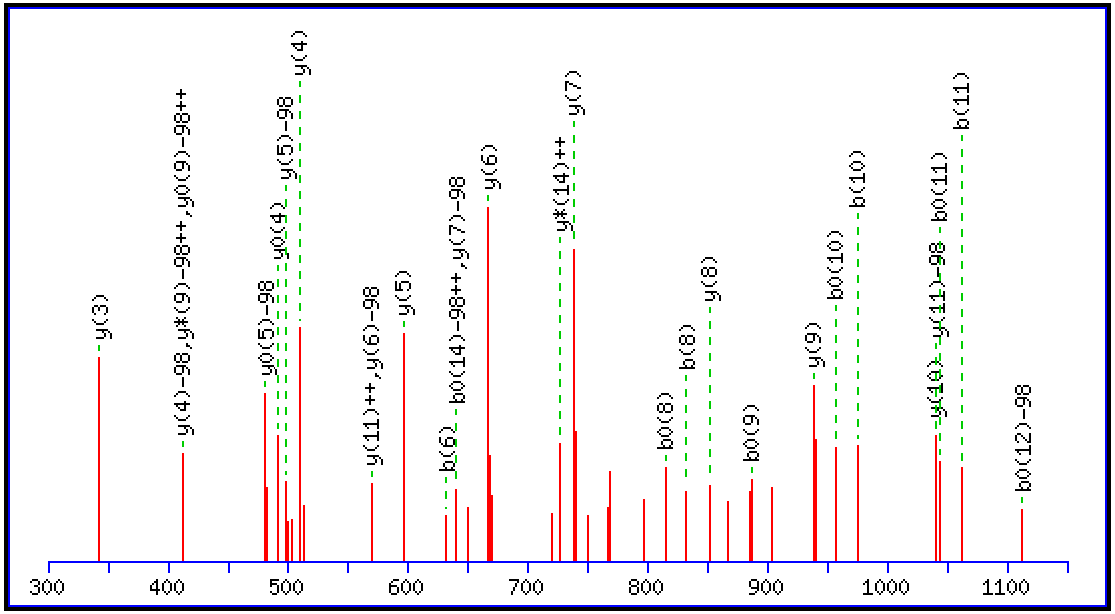

DNN(pS)AKDESITEPPAPVK

MS/MS Fragmentation of **DNNSAKDESITEPPAPVK**
Found in **AT3G27390.1**, | Symbols: | unknown protein | chr3:10133372-10136111 REVERSE

Match to Query 637: 1991.005448 from(996.510000,2+) intensity(250123.0000)
Title: Cmpd 48, +MSn(997.2), 14.9 min

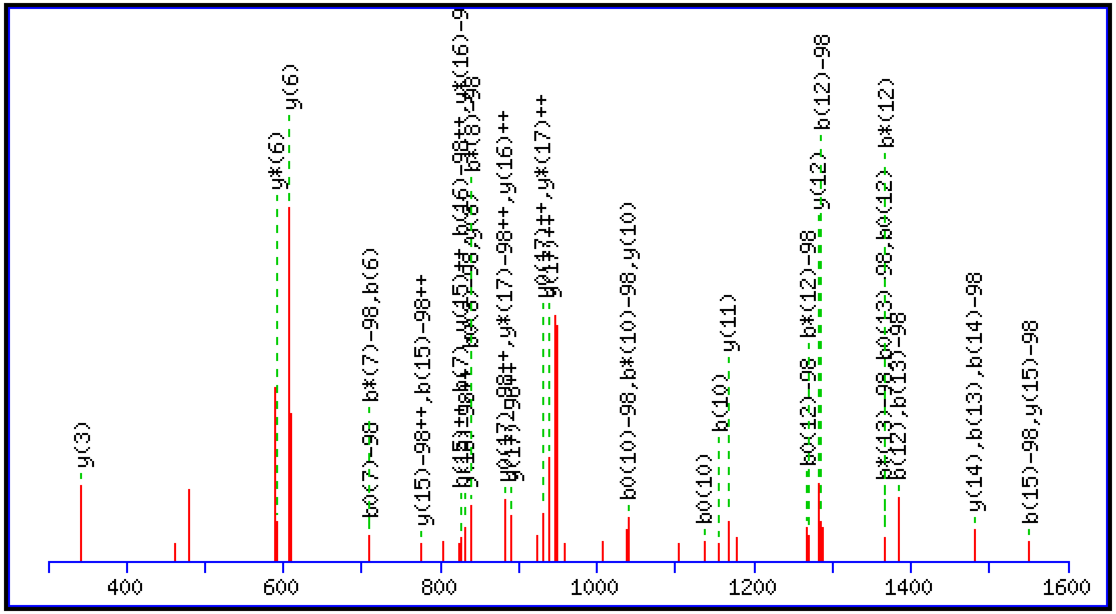

(pS)LEIEEDFDR

MS/MS Fragmentation of **SLEIEEDFDR**

Found in **AT5G64090.1**, | Symbols: | FUNCTIONS IN: molecular_function unknown; INVOLVED IN: biological_process unknown; LOCATED IN: plasma membrane; EXPRESSED IN: 23 plant structures; EXPRESSED DURING: 13 growth stages; CONTAINS InterPro DOMAIN/s: Hyccin (InterPro:IPR0186

Match to Query 108: 1331.925448 from(666.970000,2+) intensity(368723.0000)

Title: Cmpd 82, +MSn(667.4), 19.6 min

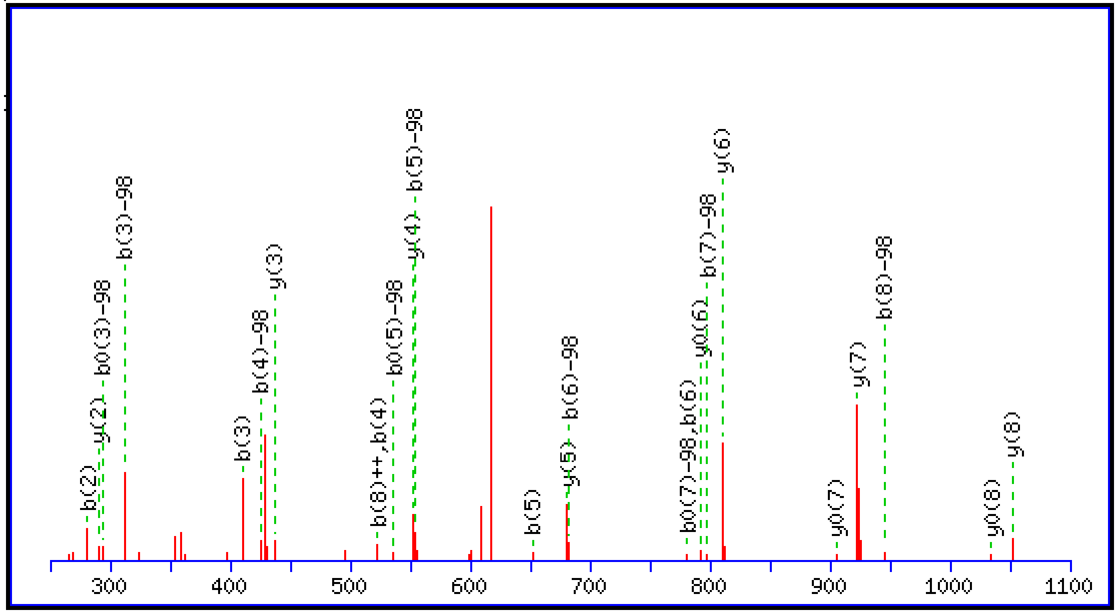

Supplement: Additional file 2 — Fragmentation data of novel and unambiguous phosphopeptides and sites. [file 1477-5956-10-62-S2.pdf]
