# Supplementary material for: Phosphoproteomic analysis reveals major default phosphorylation sites outside long intrinsically disordered regions of Arabidopsis plasma membrane proteins
Source: Proteome Sci. 2012 Oct 30;10:62. doi: 10.1186/1477-5956-10-62 (PMC3537754; doi:10.1186/1477-5956-10-62)
Supplement: Additional file 3 — Figure S2. Phosphohydrolase activities of the membrane fraction. [file 1477-5956-10-62-S3.pdf]

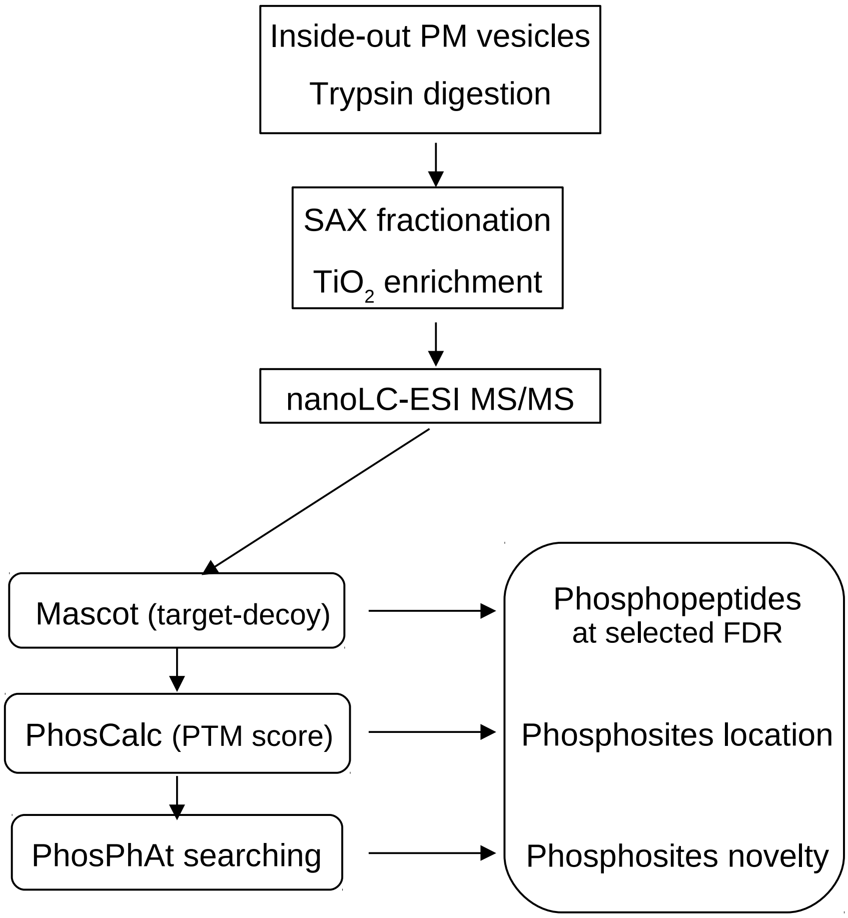

Supplementary Figure S2. **Workflow for the identification of novel phosphorylation sites in *Arabidopsis* plasma membrane.**
