# Supplementary material for: Phosphoproteomic analysis reveals major default phosphorylation sites outside long intrinsically disordered regions of Arabidopsis plasma membrane proteins
Source: Proteome Sci. 2012 Oct 30;10:62. doi: 10.1186/1477-5956-10-62 (PMC3537754; doi:10.1186/1477-5956-10-62)
Supplement: Additional file 4 — Phosphorylated proteins, peptides and sites from the bins 30 and 34 of the PhosPhAt data base. in/out, inside/outside long IDR according to IUPforest-L predictor. Only peptides containing at least one unambiguous phosphorylated residue are listed. [file 1477-5956-10-62-S4.pdf]

supplementary File F2 : phosphorylated proteins and peptides from PhosPhAt database

SIGNALING

accession	sequence	sites	bin	IDR
AT1G06840.1	G[T]PGYLDPEYFLTHQLTDK	1	30.2.8.1	out
AT1G06840.1	HHY[T]SMDVSGSDLVSGVAPSVAPR	1	30.2.8.1	out
AT1G06840.1	LAPVPDMEGI[S]PQHV[S]TVVK	2	30.2.8.1	out
AT1G09180.1	[Y]YLGLTNFTTGK	1	30.5	out
AT1G09180.1	YYLGL[T]NFTTGK	1	30.5	out
AT1G16120.1	EV[S]NELER	1	30.2.25	in
AT1G25320.1	RDG[S]E[S]P(s)(s)ENLEPQQDLVLLDK	2	30.2.3	out
AT1G25390.1	DN[S]KSDVEFSQVFFK	1	30.2.20	out
AT1G26150.1	MAFG[S]QNYSTDSLTR	1	30.2.22	out
AT1G26150.1	SD[S]ALLK	1	30.2.22	out
AT1G26150.1	TYLSQSEPGGFGQ[S]R	1	30.2.22	out
AT1G30570.1	G[S]FGYLDPEYFR	1	30.2.16	out
AT1G34210.1	DTHV[T][T]AVR	2	30.2.2	out
AT1G34210.1	LMD(y)KD(t)HV[T][T]AVR	2	30.2.2	out
AT1G34300.1	NGSLDNFLFTTD[S]AK	1	30.2.24	out
AT1G34300.1	SS[S]FSATR	1	30.2.24	out
AT1G34420.1	LEV[S]DN[S]LSGTIPEGIK	2	30.2.10	out
AT1G48480.1	A[S]AEVLGK	1	30.2.3	out
AT1G53730.1	NK[S]FDDEDSTR	1	30.2.5	in
AT1G53730.1	SFDDED[S]TRKPIAVK	1	30.2.5	in
AT1G55200.1	K[S]QANWVVLDK	1	30.2.22	out
AT1G55610.1	[S]GDVELFHYLK	1	30.2.10	out
AT1G56140.1	FDDTTAS[S]ISGFPLR	1	30.2.8.2	out
AT1G66150.1	FSGSE[S]SNAVVVHPR	1	30.2.9	out
AT1G66830.1	SDQISPMETS[S]PILSR	1	30.2.3	out
AT1G66880.1	NPTSTTISSSSNHSLLP[S]ISNLANR	1	30.2.20	out
AT1G66880.1	VLVK[S]PDVVDIECGGGDDVGLLR	1	30.2.20	out
AT1G68400.1	A[S]AEMLGK	1	30.2.3	out
AT1G70530.1	SDYITEGS[S]FFEPR	1	30.2.17	out
AT1G73080.1	ASLG[S]GK	1	30.2.11	out
AT2G01210.1	LANIAGGS[S]PTIQ[S]NR	2	30.2.3	in/out
AT2G01210.1	SV[S]SEFTAHSSSGSYYQAPETLK	1	30.2.3	in
AT2G01820.1	LLDV[S]NNDF[Y]GIPPKFR	2	30.2.9	out
AT2G01820.1	WQAFEGLSQTADDSG[S]SSSAYGSK	1	30.2.9	in
AT2G13790.1	LE[S]LVDAELEGK	1	30.2.2	out
AT2G14440.1	QGTK[Y]LIR	1	30.2.99	out
AT2G16250.1	GSWNAAIFG[S]WR	1	30.2.14	out
AT2G16250.1	RQ[S][S]EIVPEPAAYGVVEDNL	2	30.2.14	in
AT2G19190.1	[S]ILANGDIR	1	30.2.1	out
AT2G20300.1	GDLAPSDSSWWNL[T]PR	1	30.2.18	out
AT2G20850.1	ND[S]NGDNQYTGR	1	30.2.5	out
AT2G20850.1	SNASMLPPSN[T]FNK	1	30.2.5	out
AT2G23300.1	ELEVE[T]LLK	1	30.2.3	out
AT2G23770.1	AKIG[S]LGSAR	1	30.2.21	out
AT2G26730.1	GSEGQTPPGE[S]RTPPR	1	30.2.3	in

SIGNALING (continue)

accession	sequence	sites	bin	IDR
AT2G26730.1	SETTDDGLRQ[S][S]DDP[S]KG[S]EGQTPPGESR	4	30.2.3	in
AT2G26730.1	SNRL[S]GQIP[S]DFSNLTHLR	2	30.2.3	out
AT2G27030.1	ADQL[T]DDQISEFK	1	30.3	out
AT2G27060.1	IQN[S]PDNPTSR	1	30.2.3	in
AT2G36570.1	EI[S]FLKR	1	30.2.3	out
AT2G36570.1	NSM[S]PSLATTDG	1	30.2.3	out
AT2G36570.1	QFELDDLLKA[S]AEMLGK	1	30.2.3	out
AT2G36570.1	VEQ[S]PVGEDFDESR	1	30.2.3	out
AT2G37050.1	GGI[S]DEFSR	1	30.2.1	out
AT2G37050.1	T[S]AELTNRPLPIQR	1	30.2.1	out
AT2G37050.1	VS[S]TL[S]EAHGDAAHCFTLYEIEEATK	2	30.2.1	out
AT2G39360.1	NETGESLIFSS[S]K	1	30.2.16	out
AT2G43490.1	G[S]SSESVDLVSGR	1	30.5	in
AT2G48010.1	LD[S]MSESTTLVK	1	30.2.23	out
AT3G02880.1	A[S]AEVLGK	1	30.2.3	out
AT3G02880.1	ATG[S]ESGAVNK	1	30.2.3	out
AT3G02880.1	LIEEVSHSSG[S]PNPVSD	1	30.2.3	out
AT3G02880.1	NVEAPVAAATS[S]AAIPK	1	30.2.3	out
AT3G08680.1	AEEFG[S]GVQEAEK	1	30.2.3	in
AT3G08680.1	A[S]AEVLGK	1	30.2.3	out
AT3G08680.1	AS[S]PEMIR	1	30.2.3	in
AT3G08680.1	EEW[T]GEVFDVELIK	1	30.2.3	out
AT3G08680.1	LDALRII[S]LR	1	30.2.3	out
AT3G08680.1	SDNKAEEFG[S]GVQEAEK	1	30.2.3	in
AT3G14350.1	DGNLLNSGPAPPPPPG[T]PPISK	1	30.2.5	in
AT3G14350.1	GNR[S]NGDSSNSK	1	30.2.5	in
AT3G14350.1	HK[S]FDDDDSTMR	1	30.2.5	in
AT3G14350.1	KLDTSL[S]MNLRPPPSER	1	30.2.5	in
AT3G14350.1	V[T]QIKLP[S]LGLSGSLGFMLDK	2	30.2.5	out
AT3G14840.1	LLEASVNNEKDEE[S]VR	1	30.2.8.2	out
AT3G15354.1	SKLSSLCWNS[Y]IK	1	30.11	out
AT3G17840.1	AVEVN[S]SGMK	1	30.2.3	out
AT3G17840.1	SYVNEY[S]PSAVK	1	30.2.3	out
AT3G17840.1	VSDFGLAQLVSAS[S]TTPNR	1	30.2.3	out
AT3G19300.1	GD[S]GFQSGDIR	1	30.2.99	in
AT3G19300.1	GDSGFQ[S]GDIR	1	30.2.99	in
AT3G19300.1	SFLLETGSPH[S]PPNGLSF	1	30.2.99	in
AT3G21630.1	ADHASSTSLQ[S]GGLGGAGVSPGIAAISVDK	1	30.2.21	out
AT3G21630.1	VADFGLTKL[T]EVGGSATR	1	30.2.21	out
AT3G23750.1	GGFGVV[Y]AGELHDGTKTAVK	1	30.2.9	out
AT3G23750.1	GGFGVVYAGELHDG[T]KTAVK	1	30.2.9	out
AT3G24550.1	GILP[S]GKEVAVK	1	30.2.22	out
AT3G24660.1	SALYSPTETR[S]DAETPF	1	30.2.3	out
AT3G24660.1	SSIE[S]EDDLEEGDEEDEIGEK	1	30.2.3	in
AT3G28450.1	QGY[S]FSEQDDDFPLIFDTQENEKV	1	30.2.10	out
AT3G28450.1	[S]GLTEVGVSGLAQR	1	30.2.10	out
AT3G46290.1	FEES[S]IDDLSGVSMSK	1	30.2.16	out

SIGNALING (continue)

accession	sequence	sites	bin	IDR
AT3G46290.1	G[S]FGYLDPEYFR	1	30.2.16	out
AT3G46290.1	TGPELDQTHV[S]TAVK	1	30.2.16	out
AT3G46330.1	SLLVINL[S]GNK	1	30.2.99	out
AT3G47090.1	ELI[S]IR	1	30.2.12	out
AT3G47570.1	V[T]HLELGR	1	30.2.12	out
AT3G50230.1	A[S]AELLGR	1	30.2.3	out
AT3G56760.1	TAILKSS[T]EATK	1	30.3	out
AT3G57530.1	VIAEHL[S]DEEASGIR	1	30.3	out
AT3G59220.1	YKELS[S]LDIPR	1	30.5	out
AT4G02010.1	NLDAG[S]FGG[S]LPHPASTR	2	30.2.18	out
AT4G03100.1	[T]LITKTLAER	1	30.5	out
AT4G03390.1	VTVMPII[S]PERPVK	1	30.2.5	out
AT4G08850.1	QEFLNEIRAL[T]EIR	1	30.2.12	out
AT4G08850.1	QIEEH[T]D[S]ESGGETLSIFSFDGK	2	30.2.12	out
AT4G08850.1	VTEIAI[Y]DNLL[T]GPIPSSFGNLTK	2	30.2.12	out
AT4G10640.1	[S]SRNQSAGDDR	1	30.3	out
AT4G20940.1	ANHLNL[S]YNK	1	30.2.3	out
AT4G20940.1	KGSSSEIL[S]PDEK	1	30.2.3	out
AT4G20940.1	LAVATGF[S]PSK	1	30.2.3	out
AT4G23180.1	IFGLDQTEEN[T]SR	1	30.2.17	out
AT4G23250.1	IFGVDQTVAN[T]AR	1	30.2.17	out
AT4G23250.1	NGPGSNPSSQGMVPGQSS[S]K	1	30.2.17	in
AT4G23270.1	SKQEQAGPSID[S]STHCSVDEASITR	1	30.2.17	out
AT4G26540.1	NLT[S]ANVIGTGSSGVVYR	1	30.2.11	out
AT4G29810.1	AIPD[S]YLSAIFR	1	30.6	out
AT4G34220.1	STAPINPLTEKPNQ[T]GK[S]K	2	30.2.3	out
AT4G37590.1	GNV[S]ADVIGEALHAYAIK	1	30.11	out
AT5G01950.1	G[T]PGYLDPEYFLTHKLTDK	1	30.2.8.1	out
AT5G01950.1	VELASSSSVLSTSS[S]NVTR	1	30.2.8.1	out
AT5G02010.1	[T]NSKPATNTK	1	30.5	out
AT5G05160.1	CNHNFDLEDLLKA[S]AEVLGK	1	30.2.3	out
AT5G05160.1	KPQDFG[S]GVQDPEK	1	30.2.3	out
AT5G05160.1	MKIATG[T]SKAI[S][Y]LH[S]LK	4	30.2.3	out
AT5G08650.1	IPAPLD[T]AGKPLR	1	30.5	out
AT5G10020.1	FSDQPVMLDVY[S]PDR	1	30.2.3	out
AT5G10020.1	SS[S]GGSPLSSSPR	1	30.2.3	in
AT5G10290.1	LTDFE[S]PGGDAAFQR	1	30.2.2	out
AT5G13290.1	LGSLEGVG[S]PESSSSK	1	30.2.99	out
AT5G16000.1	INELMSS[S]DR	1	30.2.2	out
AT5G16590.1	A[S]AEVLGK	1	30.2.3	out
AT5G16590.1	LIEEVSRSPA[S]PGPL[S]D	2	30.2.3	out
AT5G16590.1	[S]FGEFDLDGLLK	1	30.2.3	out
AT5G24010.1	I[S]FAELQSGTNNFDR	1	30.2.16	out
AT5G24010.1	SSESTGW[T]PLR	1	30.2.16	out
AT5G24390.1	QRAPPTEDLLL[Y]VVAASVLQR	1	30.5	out
AT5G25930.1	TATEAYEAPLLVSL[S]GR	1	30.2.11	out
AT5G37450.1	ELENI[Y]GLIPK	1	30.2.8.1	out

SIGNALING (continue)

accession	sequence	sites	bin	IDR
AT5G38560.1	MAFG[S]QDYSSDFFDR	1	30.2.22	out
AT5G38560.1	SH[S]GSDYMYASSDSGMVSNQR	1	30.2.22	out
AT5G42120.1	MPGRL[S]LAEIK	1	30.2.19	out
AT5G47800.1	INNTT[Y]HLHR[S]CLVPK	2	30.11	out
AT5G49760.1	GSGDPYGSE[S]FQYSGNFPASK	1	30.2.8.1	in
AT5G49760.1	WDTSK[S]SIDAPQLMGAK	1	30.2.8.1	out
AT5G51350.1	SFG[S]PEPSEAVPASVSK	1	30.2.14	out
AT5G53320.1	EEW[T]GEVFDEELLR	1	30.2.3	out
AT5G53890.1	DL[S]VEELLK	1	30.2.10	out
AT5G53890.1	INDVDEETISGV[S]K	1	30.2.10	out
AT5G54380.1	GGVN[S]G[T]G[T]DDDAEDATTSAVFSQLVHPR	3	30.2.16	out
AT5G54380.1	SATASCI[S]LASTHLGR	1	30.2.16	out
AT5G54380.1	STSPQEGGNGHPWLPLPLYGL[S]QTLTK	1	30.2.16	out
AT5G54380.1	TGPSLDQTHV[S]TAVK	1	30.2.16	out
AT5G56890.1	GLSTSEMYTG[S]GR	1	30.2.18	in
AT5G58150.1	LSALH[Y]LNLSR	1	30.2.7	out
AT5G58150.1	VLDLSSNNLTGHVPML[S]VK	1	30.2.7	out
AT5G58300.1	QEFG[S]GVQEPEK	1	30.2.3	out
AT5G58300.1	SPVQSP[S]RDDMVDLPR	1	30.2.3	out
AT5G61350.1	INIGGDLI[S]PK	1	30.2.16	out
AT5G65700.1	DQPMTESAPE[S]ELSPK	1	30.2.11	in
AT5G65700.1	DQPMTESAPESEL[S]PK	1	30.2.11	in
AT5G65700.1	[S]GVQ[S]PPDLLNL	2	30.2.11	in
AT5G65700.1	[T]SLTGAGDDK	1	30.2.11	out
AT5G67200.1	A[S]AELLGR	1	30.2.3	out
AT5G67200.1	[S]QGMYTMEQLMR	1	30.2.3	out

TRANSPORT

accession	sequence	sites	bin	IDR
AT1G06970.1	RSVLMSY[T]WR	1	34.12	out
AT1G08090.1	EQSFAFSVQ[S]PIVHTDK	1	34.4	out
AT1G10420.1	FKLQLQ[S]MIGEVR	1	34.1	out
AT1G11260.1	GVDDV[S]QEFDDLVAASK	1	34.2	out
AT1G15210.1	DAIVGLPGVTGL[S]TEQRK	1	34.16	out
AT1G15690.1	QFN[T]IPGLMEGTAKPDYATCVK	1	34.30	out
AT1G15690.1	QFNTIPGLMEGTAKPDYA[T]CVK	1	34.30	out
AT1G17840.1	LAANAFL[S]G[T]VLLNGR	2	34.16	out
AT1G17840.1	NGTQNTTVAPDGLTQSP[S]LR	1	34.16	out
AT1G17840.1	SKWINL[S]VILSMIII[Y]R	2	34.16	out
AT1G22530.1	EF[T]APPPPPAPVK	1	34.99	in
AT1G47670.1	FL[S]PIG[T]PMKR	2	34.3	out
AT1G47670.1	V[S][T]PEILTPSGQR	2	34.3	in
AT1G48370.1	EDPPASPASPL[T]PR	1	34.13	out
AT1G48370.1	GGL[T]PDRDR	1	34.13	in
AT1G51500.1	IFGVQVTH[S]K	1	34.16	out

TRANSPORT (continue)

accession	sequence	sites	bin	IDR
AT1G57990.1	QTTAEGSANPEPDQIL[S]PR	1	34.10	out
AT1G59870.1	ISGYCEQ[T]DIHSPQVTVR	1	34.16	out
AT1G59870.1	M[S]NELAVPFDK	1	34.16	out
AT1G59870.1	NIEDIFSSG[S]RR	1	34.16	out
AT1G59870.1	SL[S]TADGNR	1	34.16	in
AT1G59870.1	TQ[S]VNDDEEALK	1	34.16	out
AT1G59870.1	WAAIEKLP[T]YSR	1	34.16	out
AT1G60160.1	I[S]MDFMRELGSTLGTIR	1	34.15	out
AT1G67940.1	VADDG[S]RILK	1	34.16	out
AT1G69870.1	IS[S]PGSILDAEK	1	34.13	out
AT1G69870.1	S[S]PSELDVVDPYKR	1	34.13	out
AT1G71880.1	[T]AGDLAGPSASVK	1	34.2.1	out
AT1G71880.1	DAAALETQ[S]PEDFDQPSPLR	1	34.2.1	out
AT1G77990.1	LGFLVDFL[S]HAALVGFMAGAAIVIGLQQLK	1	34.6	out
AT1G78900.1	GV[S]VPALDKDCLWEFQPNK	1	34.1	out
AT2G01980.1	[S]VSFGGIYNNK	1	34.14	out
AT2G01980.1	SV[S]FGGIYNNK	1	34.14	out
AT2G18960.1	[S]GLEDIKNETVDLEK	1	34.1.2	out
AT2G18960.1	[T]LHGLQPK	1	34.1.2	out
AT2G18960.1	DIDTAGHHY[T]V	1	34.1.2	out
AT2G18960.1	EDVNIFPEKG[S]YR	1	34.1.2	out
AT2G18960.1	EL[S]EIAEQAK	1	34.1.2	out
AT2G18960.1	GHVE[S]VAK	1	34.1.2	out
AT2G28070.1	MEDEAGGGDSINDATTTPVSP[S]LSK	1	34.16	in
AT2G28070.1	QPISFED[S]PEWEDTPDVDLR	1	34.16	out
AT2G28260.1	VF[S]EDLER	1	34.22	out
AT2G34660.1	LASLAEN[S]LNAVER	1	34.16	out
AT2G35060.1	LDQ[S]MDEEAGR	1	34.15	out
AT2G36910.1	NSVS[S]PIMTR	1	34.16	in
AT2G37170.1	SLG[S]FR[S]AANV	2	34.19.1	out
AT2G38940.1	AVMA[T]LCFFR	1	34.7	out
AT2G38940.1	S[T]CHGISAASGK	1	34.7	out
AT2G39010.1	[S]QLHELHA	1	34.19.1	out
AT2G39010.1	AYG[S]VRSQLHELHA	1	34.19.1	out
AT2G39010.1	TKDEL[T]EEE[S]LSGK	2	34.19.1	out
AT2G39480.1	GFQEPS[S]PK	1	34.16	out
AT2G39480.1	RQD[S]FEMR	1	34.16	out
AT2G39480.1	SNGSDPE[S]PI[S]PLLISDPQNER	2	34.16	in
AT2G40540.1	TQQEDDNAR[S]VQ[S]NE[S]SSESR	3	34.15	out
AT2G47000.1	ASE[S]GLNGDPNILEEVSETK	1	34.16	in
AT2G47000.1	H[S]FNMFGFPAGIDGNVVQDQEEDDTTQPK	1	34.16	in
AT2G47000.1	MSSIE[S]FK	1	34.16	in
AT2G47000.1	MSSIE[S]FKQS[S]LR	2	34.16	in
AT2G47800.1	[S]FLGSHIVEDGSK	1	34.16	out
AT2G47800.1	GITGLVTAE[T]NSPTKPSDAVSVEK	1	34.16	out
AT2G47800.1	SFLG[S]HIVEDGSK	1	34.16	out
AT2G47830.1	Q[T]ILQVEGVK	1	34.12	out

TRANSPORT (continue)

accession	sequence	sites	bin	IDR
AT3G05030.1	GFVPFVPG[S]PTER	1	34.14	out
AT3G06450.1	APF[S]PR	1	34.18	in
AT3G06450.1	VV[S]FQNPR	1	34.18	out
AT3G17630.1	MVEHPGITL[T]VYK	1	34.12	out
AT3G17650.1	QIVEHELQETGF[S]PETEK	1	34.13	in
AT3G24300.1	HGGFAYIYHDNDDE[S]HRVDPG[S]PFPR	2	34.5	out
AT3G24300.1	SA[T]PPRV	1	34.5	out
AT3G28860.1	LSHSL[S]TK	1	34.16	out
AT3G28860.1	NLSYSY[S]TGADGR	1	34.16	out
AT3G42640.1	L[S]VDK[S]LIEVFPK	2	34.1	out
AT3G42640.1	MITGDQLAIGIE[T]GRR	1	34.1	out
AT3G45060.1	KAMLADIP[T]PETG[S]PAHV	2	34.4	out
AT3G47780.1	RP[S]LQR	1	34.16	out
AT3G47950.1	GLDIETIQQAY[T]V	1	34.1	out
AT3G47960.1	KPLEVEPSTTTTNTDVVD[S]FEEEQR	1	34.13	out
AT3G47960.1	QQQLQDKN[S]V	1	34.13	out
AT3G53420.1	SLG[S]FR	1	34.19.1	out
AT3G53420.1	SLG[S]FR[S]AANV	2	34.19.1	out
AT3G54820.1	ALG[S]FR[S]QPHV	2	34.19.1	out
AT3G55320.1	RQD[S]FEMR	1	34.16	out
AT3G55320.1	SH[S]QTFSRPLSSPDDTK	1	34.16	in
AT3G55320.1	SNGSEPE[S]PV[S]PLLTSDPK	2	34.16	in
AT3G62150.1	MDSVIESEEGLKVD[S]PNRADAETSNSK	1	34.16	in
AT3G62150.1	QTEDSTDEQKL[S]ME[S]MKR	2	34.16	in
AT3G62700.1	[S]FLGSNIPEDGSR	1	34.16	out
AT3G62700.1	SI[S]IESPR	1	34.16	in
AT3G62700.1	SISIE[S]PRQPK	1	34.16	in
AT3G62700.1	TT[S]MESPR	1	34.16	in
AT4G05110.1	TVSADLAAAGIQNQ[S]DL[S]DDDSKNQR	1	34.14	out
AT4G13510.1	[S]PSPSGANTTPTPV	1	34.5	out
AT4G13510.1	HGGFAYMYFDDDE[S]HK	1	34.5	out
AT4G13510.1	ISSEDEMAGMDM[T]R	1	34.5	out
AT4G13510.1	RVEPR[S]PSP[S]GAN[T]TPTPV	3	34.5	out
AT4G13510.1	SP[S]PSGANTTPTPV	1	34.5	out
AT4G18910.1	SG[S]FLK	1	34.19.3	out
AT4G23640.1	SI[S]EANIAGSSR	1	34.15	out
AT4G26180.1	QLF[S]GLSINYLK	1	34.9	out
AT4G30190.1	[T]LHGLQPK	1	34.1.2	out
AT4G30190.1	EAVNIFPEKG[S]YR	1	34.1.2	out
AT4G30190.1	EL[S]EIAEQAK	1	34.1.2	out
AT4G30190.1	GLDIETPSH[Y]TV	1	34.1.2	out
AT4G30190.1	GLDIETPSHY[T]V	1	34.1.2	out
AT4G35100.1	ALG[S]FR	1	34.19.1	out
AT4G35100.1	ALG[S]FR[S]NATN	2	34.19.1	out
AT4G35100.1	ALGSFR[S]NA[T]N	2	34.19.1	out
AT4G35100.1	EV[S]EEGK	1	34.19.1	out
AT4G35300.1	GG[S]TMSVLSR	1	34.2	out

TRANSPORT (continue)

accession	sequence	sites	bin	IDR
AT4G39850.1	LIGL[S]VLQSGASSIIAPSLRHLTQR	1	34.16	out
AT5G01490.1	R[T]V[S]ASSLIR	2	34.21	in
AT5G04930.1	VRVDPVLLQL[T]K	1	34.1	out
AT5G06530.1	LELEEVSSGAAL[S]R	1	34.16	out
AT5G06530.1	TR[S]EQLYETVAADIR	1	34.16	out
AT5G09400.1	EKLEN[S]LILK	1	34.15	out
AT5G17010.1	SSGEI[S]PEREPLIK	1	34.2	in
AT5G26340.1	FMDDHNDHEFVNGEK[S]NGK	1	34.2	out
AT5G27150.1	GFVPFVPG[S]PTERNPPDLSKA	1	34.14	out
AT5G41800.1	LD[S]DAGALFVLQSK	1	34.3	out
AT5G45380.1	VVEAYA[S]GDEDVDVPAEELREEK	1	34.14	out
AT5G49890.1	I[S]GILDDGSVGFR	1	34.18	out
AT5G49890.1	TTFG[S]QILR	1	34.18	out
AT5G57110.1	GGDVESGKSEHADSDSD[T]FYIPSK	1	34.21	in
AT5G57110.1	SEHAD[S]DSD[T]FYIPSK	1	34.21	in
AT5G57110.1	[S]EHADSDSDTFYIPSK	1	34.21	in
AT5G57110.1	AIALECGIL[S]SDADLSEPTLIEGK	1	34.21	out
AT5G57110.1	FMDMGRE[S]GVEK	1	34.21	out
AT5G57110.1	SEHADSD[S]DTFYIPSK	1	34.21	in
AT5G57350.1	[T]LHGLQNTETANVVPER	1	34.1.2	out
AT5G57350.1	A[S]GLEDIVNENVDLEK	1	34.1.2	out
AT5G57350.1	GLDIETAGHY[T]V	1	34.1.2	out
AT5G59250.1	LVDDAYL[S]VK	1	34.2	out
AT5G60660.1	ALG[S]FG[S]FGSFR	2	34.19.1	out
AT5G60660.1	ALGSFGSFG[S]FR	1	34.19.1	out
AT5G61690.1	L[S]VAVALIGDPK	1	34.16	out
AT5G61740.1	QN[S]ENVLIDMEK	1	34.16	out
AT5G62670.1	[T]LHGLQAPDAK	1	34.1	out
AT5G62670.1	LKGLDIETIQQAY[T]V	1	34.1	out
